# Data distribution impacts the performance and generalisability of contrastive learning-based foundation models of electrocardiograms

**DOI:** 10.1371/journal.pdig.0001623

**Published:** 2026-09-02

**Authors:** Gul Rukh Khattak, Konstantinos Patlatzoglou, Joseph Barker, Libor Pastika, Boroumand Zeidaabadi, Aidan R. Birdi, Jiayu Huo, Ahmed El-Medany, Hesham Aggour, Yixiu Liang, Antonio H. Ribeiro, Jeffrey Annis, Antonio Luiz Pinho Ribeiro, Junbo Ge, Daniel B. Kramer, Jonathan W. Waks, Evan Brittain, Nicholas Peters, Fu Siong Ng, Arunashis Sau

**Affiliations:** 1 National Heart and Lung Institute, Imperial College London, London, United Kingdom; 2 Department of Cardiology, Chelsea and Westminster Hospital NHS Foundation Trust, London, United Kingdom; 3 Department of Cardiology, Zhongshan Hospital of Fudan University, Shanghai Institute of Cardiovascular Diseases, National Clinical Research Centre for Interventional Medicine, Shanghai, China; 4 Department of Information Technology, Uppsala University, Uppsala, Sweden; 5 Vanderbilt Institute for Clinical and Translational Research, Vanderbilt University Medical Center, Nashville, Tennessee, United States of America; 6 Center for Digital Genomic Medicine, Department of Medicine, Vanderbilt University Medical Center, Nashville, Tennessee, United States of America; 7 Division of Cardiovascular Medicine, Vanderbilt University Medical Center, Nashville, Tennessee, United States of America; 8 Department of Internal Medicine, Faculdade de Medicina, and Telehealth Center and Cardiology Service, Hospital das Clínicas, Universidade Federal de Minas Gerais, Belo Horizonte, Brazil; 9 Richard A. and Susan F. Smith Center for Outcomes Research in Cardiology, Beth Israel Deaconess Medical Center, Harvard Medical School, Boston, Massachusetts, United States of America; 10 Harvard-Thorndike Electrophysiology Institute, Beth Israel Deaconess Medical Center, Harvard Medical School, Boston, Massachusetts, United States of America; 11 Department of Cardiology, Imperial College Healthcare NHS Trust, London, United Kingdom; Shanghai United Imaging Intelligence Co Ltd, CHINA

## Abstract

Contrastive learning is a widely adopted self-supervised pretraining strategy, yet its dependence on cohort composition remains underexplored. We present Contrasting by Augmented Patient Electrocardiograms (CAPE) foundation model and pretrain on four cohorts (n = 5,203,269), from diverse populations across three continents (North America, South America, Asia). We systematically assess how cohort demographics, health status, and population diversity influence the downstream performance for prediction tasks also including two additional cohorts from another continent (Europe). We find that downstream performance depends on the distributional properties of the pretraining cohort, including demographics and health status. Moreover, while pretraining with a multi-centre, demographically diverse cohort improves in-distribution accuracy, it reduces out-of-distribution (OOD) generalisation of our contrastive approach by encoding cohort-specific artifacts. To address this, we propose the In-Distribution Batch (IDB) strategy, which preserves intra-cohort consistency during pretraining, discourages learning of spurious cohort-specific features, and instead promotes clinically meaningful variability within cohorts. This leads to improved out-of-distribution robustness, with gains of 9–40% in downstream label prediction performance. This work provides insights into pretraining strategies for more clinically deployable and generalisable foundation models.

## Author summary

Artificial intelligence (AI) can learn from large collections of electrocardiograms (ECGs) and support the development of new diagnostic tools without relying on extensive manual annotation. However, it remains unclear how the data used to pretrain these models influences their ability to generalise across different patient populations.

We developed the Contrasting by Augmented Patient Electrocardiograms (CAPE) foundation model and explored how the choice of training data influences downstream performance. Using over five million ECGs from diverse healthcare cohorts, we found that the learned representations capture not only clinically relevant cardiac information but also characteristics of the populations and healthcare systems from which the data originated. While increasing dataset size and diversity improved performance on similar populations, it could reduce accuracy when models were applied to unseen cohorts.

We also developed an In-Distribution Batch (IDB) approach to help models focus on meaningful ECG patterns rather than differences specific to individual cohorts. This improved model performance on unseen populations by 9–40%. These findings highlight that the composition of pretraining data is a key determinant of model generalisation and provide a strategy for developing ECG foundation models that are more reliable across diverse patient populations.

## Introduction

Electrocardiograms (ECGs) provide a non-invasive and widely accessible means of recording the heart’s electrical activity, capturing myocardial depolarisation and repolarisation across multiple leads positioned on the skin. Despite the potential to reveal important physiological insights, the clinical utility of ECGs remains in part constrained by the need for expert interpretation. Additionally, recent studies have demonstrated the potential for artificial intelligence-enhanced ECG (AI-ECG) analysis to identify undiagnosed disease and predict risk of adverse events better than human experts [1–4].

Foundation models learn general patterns from large datasets, which enhances performance, accelerates learning, and reduces the need for extensive task-specific annotations [5]. While they can be pretrained with supervised learning [6], self-supervised learning (SSL) has become the predominant paradigm, with common approaches including contrastive learning [7] and masked modeling [8]. These SSL methods enable models to learn rich, transferable representations that generalise across a wide range of tasks and domains. The abundance of unlabelled ECG data, coupled with the high cost of expert annotation, highlights the promise of SSL as an effective pretraining strategy for ECG analysis. Among SSL approaches, contrastive learning has gained prominence in the medical domain [9], for its ability to learn meaningful representation (features) by promoting similarity between contextually positive pairs and dissimilarity from negatives [7]. It has been applied to ECGs with various definitions of positive and negative pairs, involving contrasting different signals [10–12] or applying a range of signal transformations (augmentations) to the same signal [13–15].

A key question is whether the characteristics of the pretraining data significantly affect foundation model performance. In computer vision, prior work has shown that dataset composition can affect representations learned through contrastive self-supervised learning, while also demonstrating a degree of robustness across different dataset types [16]. Similarly, in the ECG domain, a prior study using self-supervised pretraining across medium-sized secondary-care datasets reported comparable out-of-distribution performance despite substantial distributional differences between cohorts [17]. However, it remains unclear whether these observations generalise to datasets derived from different healthcare levels with varying diversity and size. This question is particularly relevant in ECG analysis, where signals from healthy individuals are often relatively homogeneous, while pathological conditions introduce structured but heterogeneous deviations. A large-scale systematic investigation of ECG data is therefore needed.

Deep learning models often struggle with generalisation, particularly when evaluated on out-of-distribution (OOD) data [18,19]. This is complicated further by the ability to learn complex patterns beyond human capabilities, as demonstrated by the ability to predict patient age or sex from ECG data [4]. However, this strength also makes them prone to overfitting the training data. Performance has been shown to vary with patient health status [20] and may be influenced by demographic factors such as ethnicity, which have been associated with identifiable signatures in ECG signals [21]. This challenge is further compounded by significant heterogeneity across cohorts, stemming from differences in age distribution, clinical profiles, recording devices, and institutional practices [20,22]. The ECG-based models experience degraded performance when tested on data from different sources, proving that domain shifts across the ECG cohorts can affect model generalisation [23].

Foundation models must overcome domain shift to be clinically viable, a challenge observed across multiple medical modalities. For example, a retinal image-based foundation model trained on British patient data demonstrated poor transferability to Chinese cohorts [24]. In parallel, evidence of socio-demographic bias has also been reported in foundation models used for medical diagnosis based on large language models [23], highlighting broader concerns regarding fairness and robustness across diverse populations. Together, these findings underscore the critical need for robust generalisation across diverse populations to ensure the practical utility of foundation models in biomedical applications.

Several approaches have been proposed to improve domain generalisation [25] across diverse domains. Uncertainty-aware and ensemble-based foundation models [26] improve external validation performance in glioblastoma classification by leveraging multiple models and uncertainty estimation to better distinguish tumours from their mimics. Contrastive learning with fairness objectives [27] enhances fairness in pathology AI systems by incorporating fairness constraints into the learning objective. For ECG tasks, self-supervised contrastive learning [17] and aggregation of features from multiple intermediate layers [28] have been crucial for improving generalisation, collectively highlighting the need for models that generalise across diverse clinical environments.

Contrastive learning performance inherently depends on how positive and negative samples are defined, so we hypothesise that cohort distribution can significantly influence the quality of the learned features. We explore the generalisation and robustness of our Contrasting by Augmented Patient Electrocardiograms (CAPE) foundation model [29], which integrates within-patient contrastive strategy [11] with signal-preserving augmentations such as random cropping and zero masking. Using a multi-centre cohort of over five million ECGs, we investigate how data diversity, label distribution, clinical setting, and cohort composition during pretraining affect the robustness and generalisability of ECG representations. To address the challenges introduced by heterogeneous data sources, we further propose the **In-Distribution Batch (IDB)** approach, which constrains contrastive learning batches to single cohorts to reduce distributional noise. This work aims to guide the development of clinically meaningful foundation models that can generalise effectively across diverse patient populations and healthcare environments.

## Results

### Cohorts

This study utilises a diverse set of large-scale electrocardiogram (ECG) cohorts from five countries across four continents: the Beth Israel Deaconess Medical Centre (BIDMC) [3] and Vanderbilt University Medical Centre (VUMC) [30] from the United States, the Clinical Outcomes in Digital Electrocardiography (CODE) cohort [31] from Brazil, the Shanghai Zhongshan Hospital (SHZS) cohort from China, the UK Biobank (UKB) [32] from the United Kingdom, and the Physikalisch-Technische Bundesanstalt (PTB-XL) dataset [22] from Germany. Among these, BIDMC, PTB-XL, and VUMC originate from secondary care settings and are characterized by a higher prevalence of cardiovascular disease.

Table 1 summarises the characteristics of the unlabelled ECG data from patients with multiple recordings used in contrastive pretraining from BIDMC, CODE, SHZS, and VUMC cohorts. The combined BCSV cohort includes over five million unlabelled ECGs drawn from BIDMC (127,041 patients; 1,106,886 ECGs), CODE (424,577 patients; 1,123,903 ECGs), SHZS (420,956 patients; 1,560,551 ECGs), and VUMC (252,294 patients; 1,411,929 ECGs). Mean patient ages are 64.46 years (BIDMC), 56.57 years (CODE), 54.15 years (SHZS), and 60.21 (VUMC); the percentages of male subjects are 52% (BIDMC), 39% (CODE), 56% (SHZS), and 54% (VUMC). The 5-year mortality (among patients with 5-year follow-up) for the secondary care cohorts, BIDMC and VUMC, is 35% and 32%, respectively, compared with15% for the CODE cohort.

**Table 1:** Baseline characteristics of the pretraining cohorts included in this study (p < 0.0001 for all pairwise comparisons of age, sex, and mortality).

	BIDMC^*^	CODE^*^	SHZS^*^	VUMC^*^
Location	US	Brazil	China	US
Care level	secondary	primary	primary	secondary
Patients^*^	127,041	424,577	420,956	252,294
ECGs	1,106,886	1,123,903	1,560,551	1,411,929
Age mean	64.46	56.57	54.15	60.21
Age IQR	21.09	24.00	26.00	22.24
Male	571,944 (52%)	437,188 (39%)	874,969 (56%)	751,040 (54%)
Mortality (5y)**	236,294 (35%)	5,125 (16%)	_	89,900 (32%)
Hispanic	7,077	−	−	−
White	84,265	−	−	−
Black	17,778	−	−	−
Asian	5,315	−	−	−
Other	12,606	−	−	−

* Pretraining cohort with patients having more than one ECG

** Includes only ECGs with five-year follow-up

The downstream prediction tasks employ BIDMC, CODE, SHZS and together with additional cohorts including UKB, a large volunteer cohort comprising 70,655 ECGs from 66,402 patients (mean age 65.31 years; 8% male), and PTB-XL, an open-access secondary care dataset containing 21,799 ECGs from 18,869 patients (mean age 62.77 years; 52% male). The demographic and health characteristics of the cohorts used for label prediction are presented (Table 2). The 5-year mortality among patients with five-year follow-up in the labelled cohort is 35% for BIDMC, 26% for VUMC, 9% for CODE, and 0.4% for the UKB.

**Table 2:** Baseline characteristics of the cohorts explored for the downstream supervised tasks (BIDMC, CODE, SHZS, UKB, and PTB-XL). p < 0.0001 for all pairwise comparisons of age, sex, and mortality.

	BIDMC	CODE	SHZS	VUMC	UKB	PTB-XL
Location	US	Brazil	China	US	UK	Germany
Care level	secondary	primary	primary	secondary	primary	secondary
Patients^*^	189,542	1,503,229	1,117,197	526,017	66,402	18,869
ECGs	1,169,387	2,202,555	2,257,485	1,685,652	70,655	21, 799
Age mean	63.73	53.54	53.62	58.76	65.31	62.77
Age IQR	21.67	25.00	26.00	23.73	12.00	22.00
Male	599,731 (51%)	878,668 (40%)	1,235,353 (55%)	876,790 (53%)	34,265 (49%)	11,354 (52%)
Mortality (5y)*	242,085 (35%)	12,575 (9%)	–	104,130 (26%)	68 (0.4%)	–
Hispanic	10,248	−	−		−	−
White	123,063	−	−		−	−
Black	24,251	−	−		−	−
Asian	8,924	−	−		−	−
Other	23,056	−	−		−	−

* Includes only ECGs with five-year follow-up.

### Training

The training pipeline consists of two stages: contrastive pretraining followed by supervised learning for prediction tasks. Initially, CAPE models are pretrained to learn generic ECG features, which are then generated for each labelled cohort. Supervised learning is performed in a manner similar to a linear probe, except that a non-linear multi-layer perceptron (MLP) prediction head is used. Linear probing is a standard methodology to compare different pretraining approaches and assess the quality of learned representations without any bias introduced by downstream optimisation (e.g., SimCLR [7]. In addition, representations learned by foundation models are often adapted to downstream applications using lightweight prediction heads rather than computationally expensive end-to-end fine-tuning. In our experiments, leveraging precomputed features substantially reduced training time, which was essential given the scale of the study.

Supervised tasks implemented in the current work include predicting age and sex, as well as reduced left ventricular ejection fraction and five-year mortality. Demographic labels such as age and sex are available across all cohorts and have been shown to be implicitly encoded in ECG signals [4,20], enabling broader comparison while remaining sufficiently challenging and representative. In contrast, clinical labels are heterogeneous across datasets and not uniformly available; thus, we use these tasks to further corroborate the primary findings from age and sex.

### Composition of pretraining cohort affects performance

**Fig 1:**
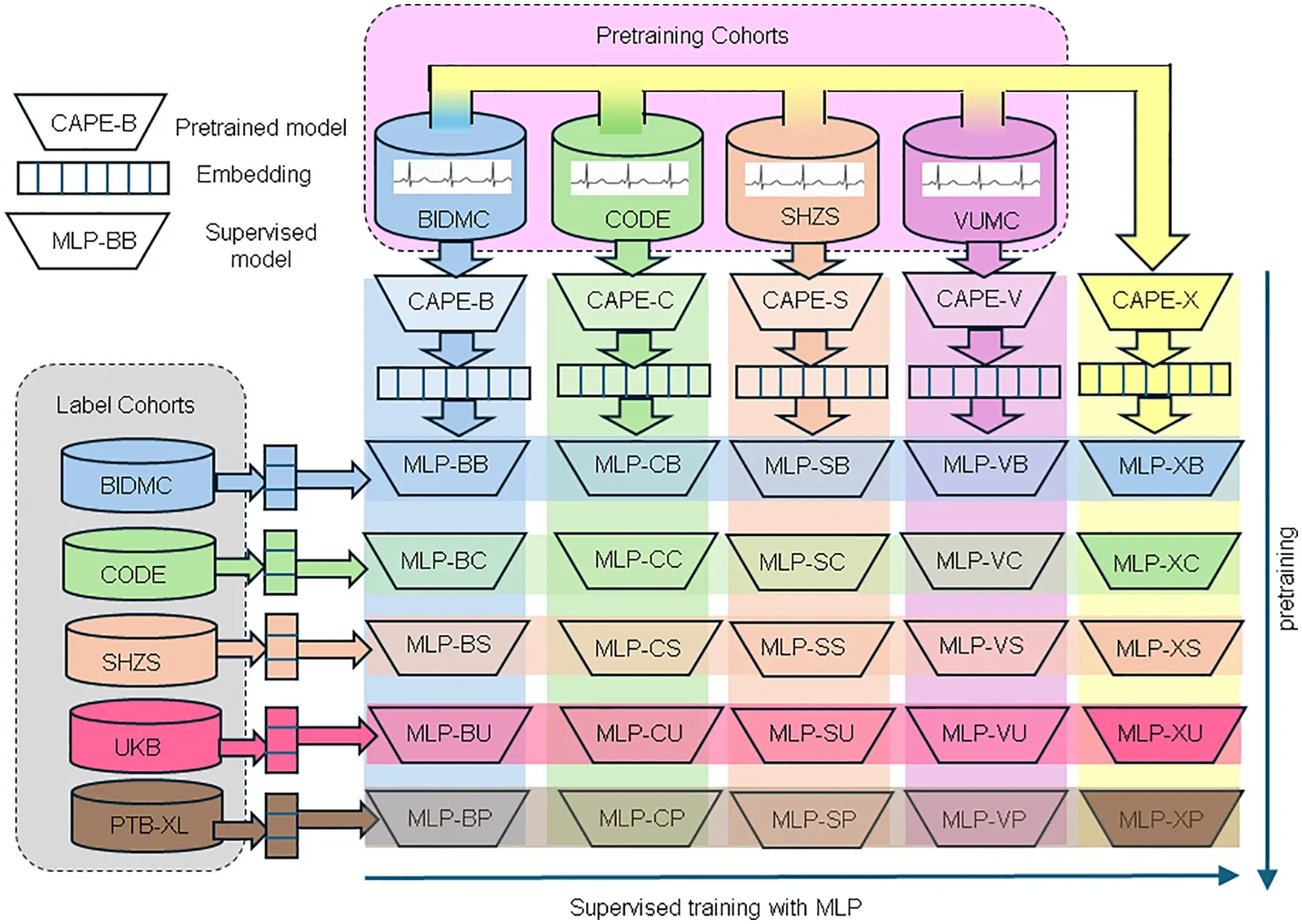
Experimental configuration for assessing the impact of cohort health and demographics on model performance. Five CAPE models are pretrained on distinct datasets: BIDMC, CODE, SHZS, VUMC, and BCSV. For each pretrained model, supervised prediction heads are trained on 10,000 randomly sampled instances and evaluated on 2,000 random test samples, resulting in 25 distinct downstream evaluations. Pretrained models are denoted as CAPE-α, where α refers to the pretraining dataset. The corresponding multilayer perceptron (MLP) models used for supervised prediction are denoted as MLP-αβ, where α indicates the pretraining dataset and β represents the labelled dataset used for training the prediction head.

We explored how the demographic composition of pretraining datasets influences the quality of learned ECG representational features and the accuracy of downstream predictive models. Five CAPE models were pretrained on distinct cohorts with varied characteristics: BIDMC (CAPE-B), CODE (CAPE-C), SHZS (CAPE-S), VUMC (CAPE-V), and the multicentre BCSV dataset (CAPE-X) (Fig 1). Downstream tasks were implemented for a range of labelled cohorts, including those included in the pretraining, as well as additional cohorts such as PTB-XL and UKB. Each CAPE model extracted features from the label cohorts, which were then used to train multi-layer perceptron networks (MLP) for age and sex prediction, yielding twenty-five models per task. For all cohorts, we applied a consistent split of 10,000 samples for training, 2,000 for validation, and 2,000 for testing. These sizes were chosen to ensure that every cohort could provide sufficient samples, so that training, validation, and testing were all performed on the same number of examples. This approach eliminates biases due to sample size and enables a fair comparison.

**Fig 2:**
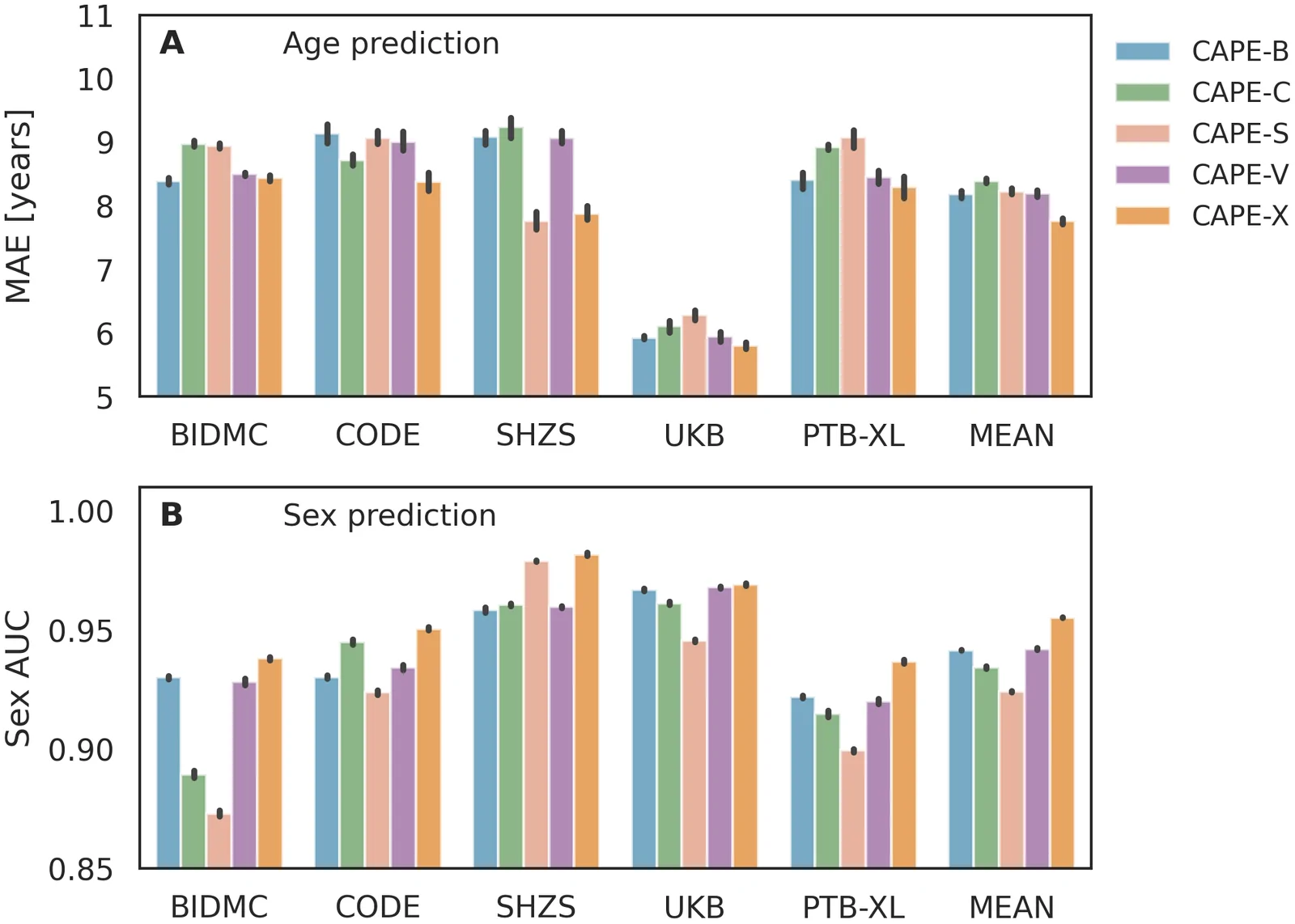
Performance comparison across different pretraining and labelled datasets. (A) Age prediction and (B) sex prediction results are shown. Colors correspond to CAPE models pretrained with distinct cohorts. The x-axis represents the labelled dataset used for supervised training, while the y-axis indicates the performance metric under evaluation. Notably, the CAPE model pretrained on BCSV consistently achieves the highest performance across all labelled datasets.

Performance is reported as mean absolute error (MAE) for age regression (Fig 2A) and area under the ROC curve (AUROC) for sex classification (Fig 2B), averaged over six runs. Models pretrained on the multi-centre BCSV dataset (CAPE-X) achieve the best overall performance, demonstrating the benefit of pretraining on a large and diverse dataset. Performance varies across individual label cohorts; therefore, we report the mean across all label cohorts. CAPE-X achieve the lowest mean MAE for age (7.86, 95% CI: 7.70–7.82), with overall differences to other models being significant (Kruskal–Wallis, *p* = 0.0002). Post hoc Holm–Bonferroni-corrected comparisons (S1 Text) indicated that mean MAE from CAPE-B, CAPE-S, and CAPE-V does not differ significantly, whereas all other pairwise differences were significant (*p*_*adj*_ = 0.0395). For sex prediction, CAPE-X achieve the highest mean AUROC (0.955, 95% CI: 0.955–0.956), with overall differences between models being significant (Kruskal–Wallis, *p* < 0.0001). Post hoc comparisons showed no significant difference between CAPE-B and CAPE-V, whereas all other pairwise differences were significant (*p*_*adj*_ = 0.0395). Among CAPE models pretrained on single cohorts, those trained on secondary care datasets such as BIDMC (age MAE: 8.19, 95% CI8.10–8.28; sex AUROC: 0.942, 95% CI: 0.941–0.942) and VUMC (age MAE: 8.20, 95% CI: 8.12–08.27; sex AUROC: 0.942, 95% CI: 0.942–0.943) outperform those trained on healthier population-based cohorts such as CODE (age MAE: 8.40, 95% CI: 8.35–8.45; sex AUROC: 0.934, 95% CI: 0.934–0.935) and SHZS (age MAE:8.23, 95% CI: 8.16–8.30; sex AUROC: 0.924, 95% CI: 0.924–0.925, with differences significant at *p*_*adj*_  < 0.05.

### Out-of-distribution performance decline in multi-cohort contrastive pretraining

**Fig 3:**
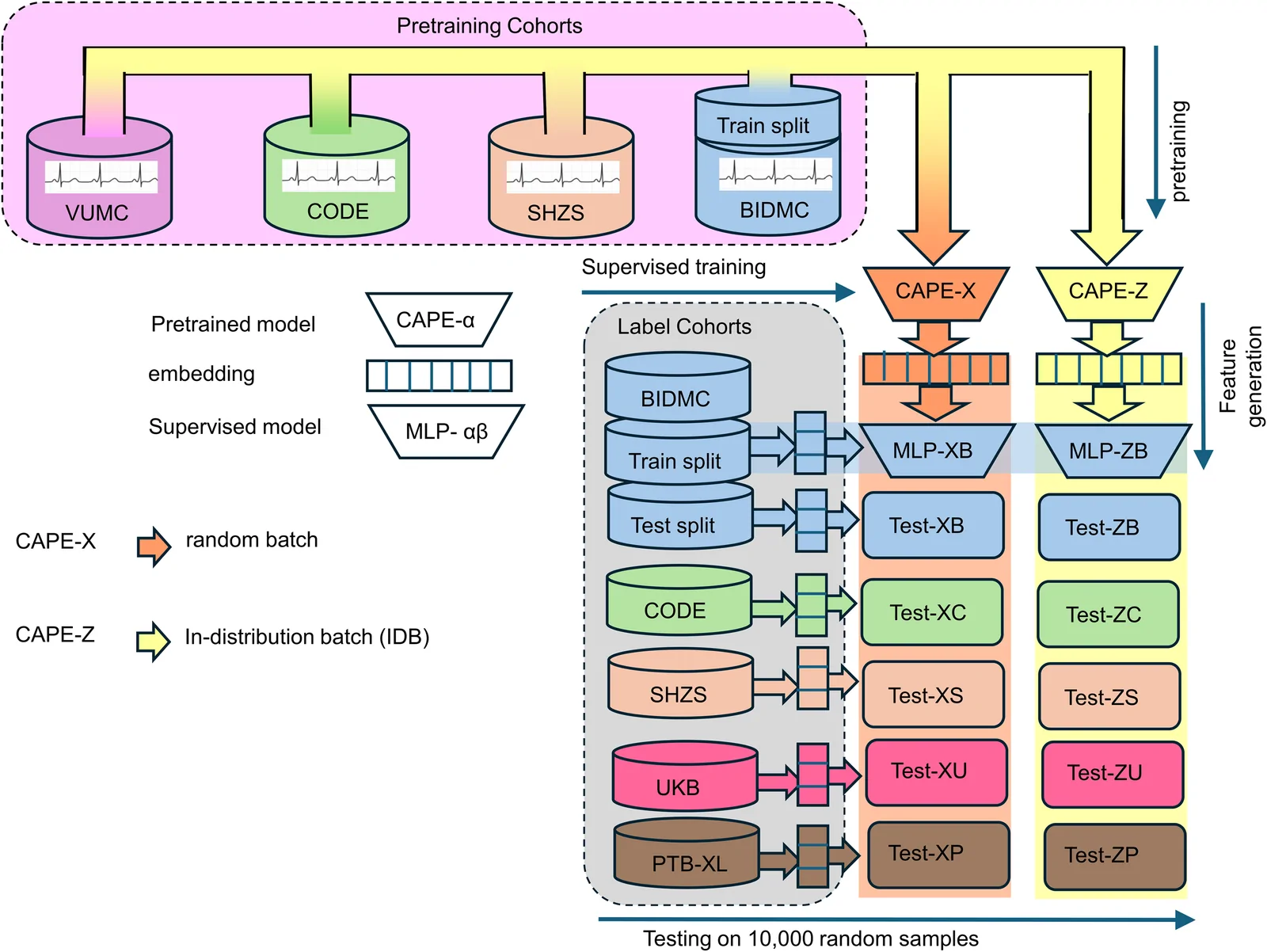
Experimental configuration for evaluating out-of-distribution (OOD) performance. The experiment involves CAPE models pretrained on the combined BCSV dataset, using either random training batches (CAPE-X) or in-distribution (ID) batches (CAPE-Z). Downstream MLP supervised heads are trained on BIDMC labels across ten independent runs. Models are evaluated on 10,000 random samples drawn from each cohort. Pretrained models are denoted as CAPE-α, where α corresponds to the first letter of the pretraining dataset. The MLP models are denoted as MLP-αβ, with α indicating the pretrained model and β indicating the labelled training cohort. For the test results, Test-αβ denotes evaluation of the model pretrained on α and trained on β.

We evaluated out-of-distribution (OOD) generalisation of supervised prediction models trained on representations extracted from a single cohort and tested on independent external cohorts (Fig 3). While prior analyses confirmed that CAPE-X achieved the highest average performance when models were trained and tested on the same dataset, here we explore the robustness of these representations under the distributional shift by conducting supervised training on one cohort and testing on others.

The CAPE-X model was pretrained on the combined BCSV dataset, with batches randomly sampled across multiple source cohorts. Following pretraining, feature representations were extracted for the BIDMC cohort (n = 1,169,387), and a separate multilayer perceptron classifier, designated MLP-XB, was trained for each prediction task. The BIDMC features were partitioned into training, validation, and test sets comprising 50 percent, 10 percent, and 40 percent of the data, respectively. For this test we excluded the BIDMC test split from the pretraining to further ensure an unbiased evaluation. Supervised training was repeated across ten independent runs using the predefined splits to ensure robust performance estimates.

To assess OOD generalisation, each trained MLP-XB model was evaluated on randomly sampled subsets of 10,000 instances from each of the external cohorts using the same seed. This sampling strategy enables assessment of performance variability across independent runs while accounting for differences in cohort sizes and distributions, which is essential given the heterogeneity in cohort size and demographic composition.

Quantitative evaluation (Table 3) reveals a marked performance degradation of the MLP-XB for CAPE-X features when applied to external cohorts, particularly CODE (age MAE: 24.00, 95% CI 18.45–29.55 & Sex AUROC: 0.598, 95% CI 0.567–0.630) and SHZS (age MAE: 14.47, 95% CI 10.63–18.32 & Sex AUROC: 0.652, 95% CI 0.564–0.740), despite the broad and diverse pretraining base of CAPE-X.

**Table 3:** Performance comparison of foundation models pretrained using random batch versus in-distribution batch (IDB) strategies. The prediction head was trained on BIDMC labels across ten independent runs and evaluated on 10,000 randomly sampled test ECGs from external cohorts. Performance metrics are reported as mean values with standard errors (SE) in parentheses; for example, a mean absolute error (MAE) of 7.880 with an SE of 0.0028 is reported as 7.880(028), and an area under the curve (AUC) of 0.939 with an SE of 0.0006 as 0.939(06).

No.	Model	BIDMC	CODE	SHZS	UKB	PTB-XL	mean
Age prediction in years (MAE)							
1	CAPE-X	**7.880(028)**	24.232(*)	14.326(*)	5.786(018)	7.786(019)	12.00
2	CAPE-Z	7.833(031)	**7.962(031)**	**7.378(020)**	**5.875(017)**	**7.681(017)**	**7.346**
3	p-value^!^	0.004	0.002	0.002	0.002	0.010	–
Sex prediction (AUC)							
4	CAPE-X	**0.939(06)**	0.600(*)	0.659(*)	0.974(04)	0.937(05)	0.822
5	CAPE-Z	0.937(08)	**0.935(08)**	**0.942(07)**	**0.977(03)**	**0.947(06)**	**0.948**
6	p-value^$^	<0.0001	<0.0001	<0.0001	<0.0001	<0.0001	**–**
LVEF < 40 (AUC)							
7	CAPE-X	0.894(017)	–	0.581(211)	**0.874(083)**	–	0.783
8	CAPE-Z	**0.896(017)**	–	**0.965(020)**	0.867(107)	–	**0.910**
9	p-value^$^	0.0217	–	<0.0001	0.326	–	–
5 year mortality (AUC)							
10	CAPE-X	**0.793(022)**	0.650(230)	–	0.662	–	0.702
11	CAPE-Z	0.792(023)	**0.819(018)**	–	**0.676**	–	**0.762**
12	p-value^$^	0.235	<0.0001	–	<0.0001	–	–

* Out of range with SE(MAE) ≥ 1.0 or SE(AUC) ≥ 0.01.

! Wilcoxon signed-rank test

$ DeLong test

### Pretraining with in-distribution batches improves generalisation

**Fig 4:**
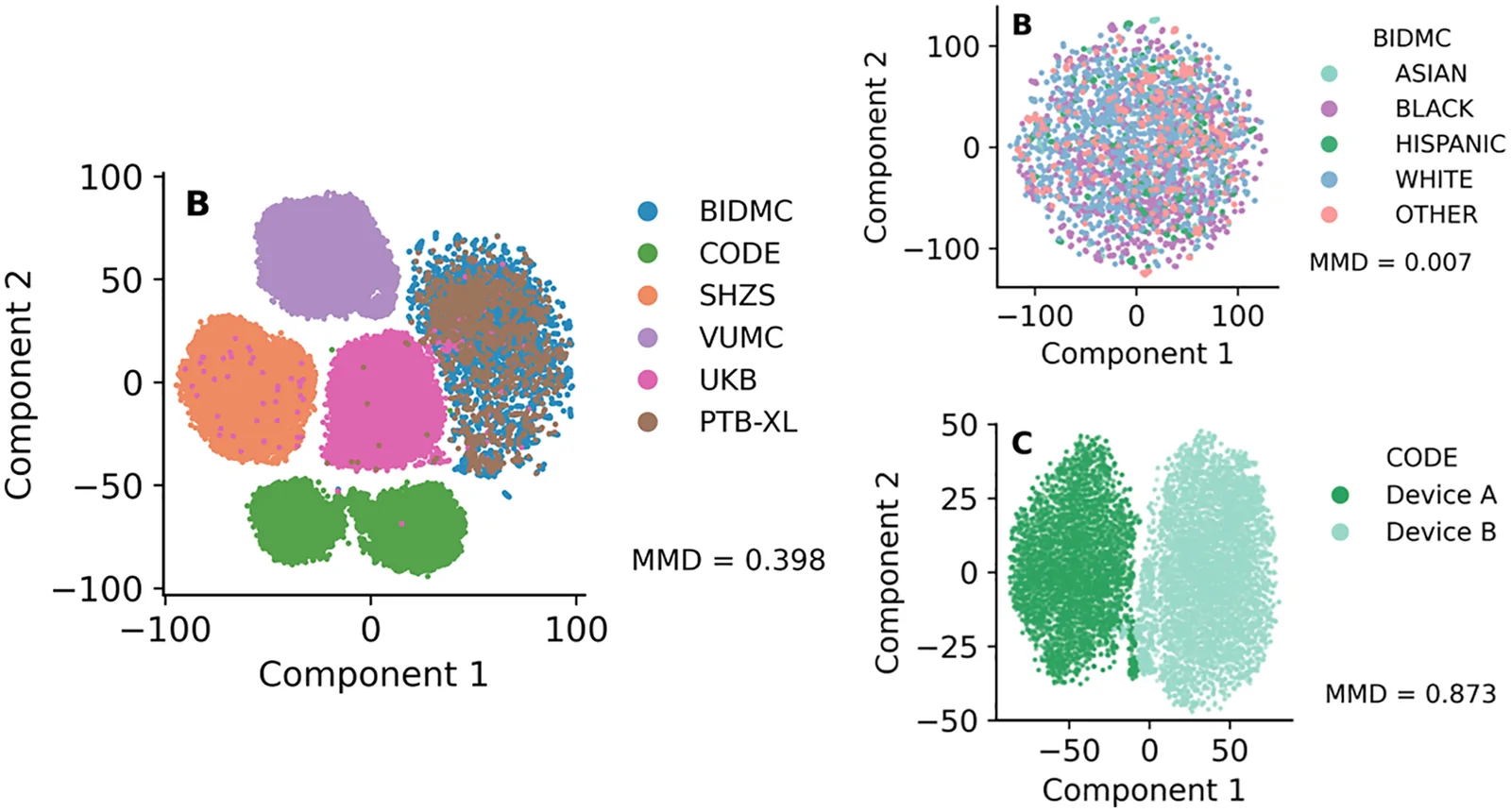
t-SNE visualization of learned embeddings from the CAPE-X model form clusters across cohorts. The CAPE-X model was pretrained on the combined BCSV dataset using random batches. (A) The learned feature representation, projected onto the first two components, shows distinct clustering by cohort, with overlap observed between BIDMC and PTB-XL. (B) Within the BIDMC cohort, features from different ethnic groups exhibit substantial overlap, with low distributional separation in the component space (MMD  =  0.007). (C) In contrast, features from the CODE dataset form two clearly separated clusters corresponding to recording device types, indicating strong distributional shift in the component space (MMD  =  0.873).

**Fig 5:**
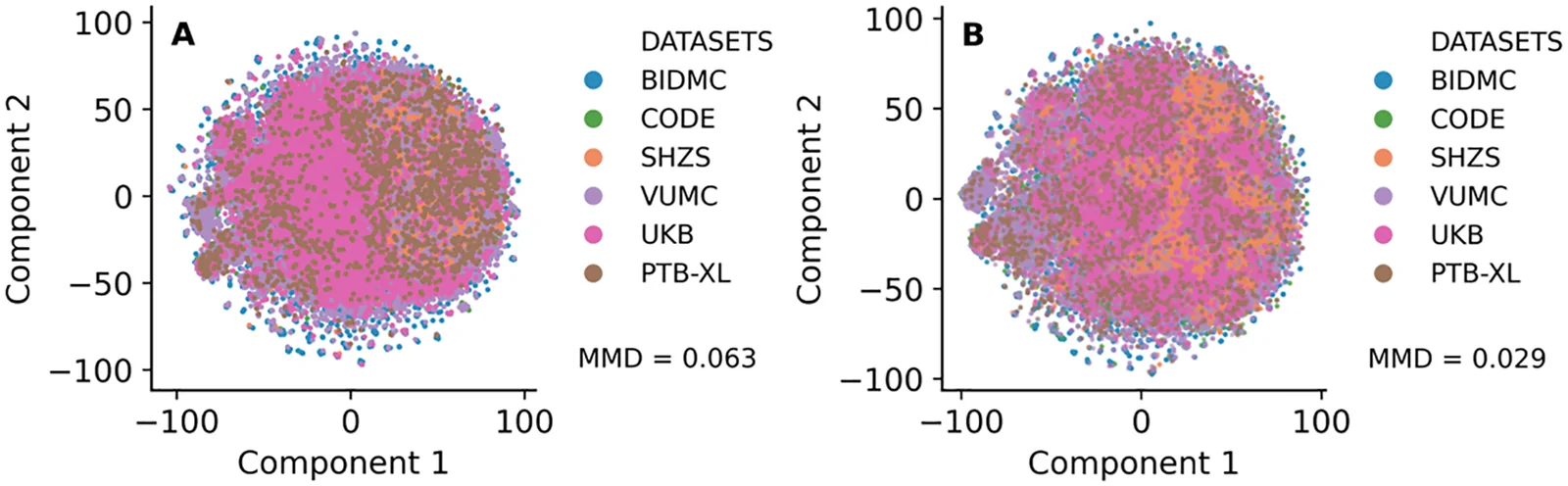
t-SNE visualization of learned embeddings. Feature embeddings overlap across cohorts without forming distinct clusters. A) CAPE-B, pretrained on BIDMC alone, shows no clear clustering, although a weak cohort-associated gradient is apparent. B) CAPE-Z, pretrained on the combined BCSV dataset using the in-distribution batch (IDB) strategy, likewise shows no apparent cohort-specific clustering.

To further investigate this degradation, we conducted a qualitative analysis using t-distributed stochastic neighbour embedding (t-SNE), a nonlinear dimensionality reduction technique for visualizing high-dimensional representations [33]. We quantify distributional differences between groups using Maximum Mean Discrepancy (MMD), a non-parametric metric that measures the distance between two probability distributions in a reproducing kernel Hilbert space. Higher MMD values indicate greater separation between group feature distributions, while lower values suggest substantial overlap. The t-SNE projections of CAPE-X features (Fig 4A) exhibit distinct clustering by cohort (MMD = 0.398), indicating that the model encodes cohort-specific information. Visualisations of the first two t-SNE components show that within BIDMC, ethnicity distributions (Fig 4B) exhibit minimal separation (MMD = 0.007), while within CODE, device types (Fig 4C) show strong separation (MMD = 0.873). This suggests that technical factors may contribute more strongly than patient demographics to the dominant structure observed in the t-SNE representation. By contrast, features generated by the CAPE-B model (described in previous section) display a continuous and more homogeneous distribution across cohorts (Fig 5A), indicating a reduced in cohort specificity (MMD = 0.089). These findings suggest that even with standardized preprocessing, technical variation introduced by different acquisition devices or pipeline artifacts can bias the learned representations and reduce their ability to generalise across settings.

To address this challenge, we introduce a novel training approach termed In Distribution Batching (IDB), implemented in the CAPE-Z model (Fig 3). In this strategy, each training batch is composed exclusively of samples from a single cohort, ensuring that contrastive negative pairs originate from the same distribution. This design discourages the model from learning spurious cohort-distinguishing features and instead promotes the identification of clinically meaningful variability within each cohort. Features produced by CAPE-Z (Fig 5B) exhibit further reduced clustering in t-SNE space (MMD = 0.029), suggesting improved robustness to distributional differences. Table 3 compares performance and, consistent with these observations, shows that CAPE-Z maintains stable performance across cohorts. In the CODE cohort, CAPE-Z achieves an age prediction MAE of 7.93 (95% CI: 7.60–8.26) and a sex classification AUROC of 0.927 (95% CI: 0.913–0.941), both statistically significant compared to CAPE-X (p < 0.001). Similarly, in the SHZS cohort, the age MAE is reduced to 7.24 (95% CI: 7.04–7.44), and the AUROC for sex classification reaches 0.935 (95% CI: 0.924–0.946), with highly significant improvements over CAPE-X (p < 0.001). We further evaluated fine-tuned models (S5 Table), in which the CAPE feature-generating backbone was also updated during training. The performance deterioration observed for CAPE-X in external validation persisted after fine-tuning, whereas CAPE-Z demonstrated robust generalisation across cohorts.

To assess whether age and sex serve as representative benchmarks for cardiac prediction tasks, we further evaluated two clinically relevant outcomes: left ventricular ejection fraction (LVEF) <40% and five-year mortality. Classifiers were trained on the BIDMC cohort and externally validated on the cohorts where these labels were available. We observed significant improvements for LVEF <40% in the SHZS cohort with AUROC of 0.581 (95% CI: 0.534–0.629) for CAPE-X vs 0.965 (95% CI: 0.960–0.970) for CAPE-Z (p < 0.0001). Similarly, five-year mortality in the CODE cohort has AUROC of 0.650 (95% CI: 0.598–0.702) for CAPE-X vs 0.819 (95% CI: 0.815–0.823) for CAPE-Z (p < 0.0001) (p < 0.0001). In addition, the area under the precision–recall gain curve (AUPRG), a metric particularly suited to imbalanced data, shows consistent performance trends (S1 Table). Sex-stratified analyses of five-year mortality likewise reveal no consistent performance differences between models across cohorts, with observed performance disparities primarily attributable to cohort-level variation rather than model architecture (S2 Table).

## Discussion

To our knowledge, this is the first systematic exploration of how different pretraining cohorts influence learned feature representations, and how varying downstream cohorts affect predictive performance on target labels. Within our contrastive learning framework, we find that combining multiple pretraining cohorts markedly reduces the generalisation ability of downstream models to out-of-distribution (OOD) data. To mitigate this effect, we introduce an In-Distribution Batch (IDB) strategy, which explicitly aligns pretraining distributions with the intended downstream application.

Foundation models leverage large-scale cohorts to learn rich and generalisable feature representations. This capability is particularly valuable in medical applications, where annotated datasets are typically limited in size and exhibit significant class imbalance or skewed distributions. The role of foundation models in medicine is rapidly expanding [34], demonstrating enhanced performance in diagnostic tasks across modalities such as X-rays [35], CT scans [36], and electrocardiograms [14]. Despite these advances, a fundamental challenge remains achieving robust generalisation to real-world clinical data, which frequently exhibit substantial distributional shifts [37]. Models trained on medical data from one cohort could not be generalised to another population from another continent across diverse modalities [23,24]. Such limitations have motivated approaches to improve generalisation, including the use of intermediate-layer features from residual networks [28], ensemble-based foundation models [25], and fairness-aware contrastive learning [26]. Although the current work focuses on contrastive learning, cohort-dependent effects on representation learning and downstream generalisation may also extend to alternative foundation model paradigms, including masked autoencoders and generative pretraining approaches.

### Pretraining data distribution affects the quality of the learned representations

The influence of health status and demographic factors on learned representations is of critical importance for successful clinical translation. Within supervised learning paradigms, model performance has been observed to differ significantly between healthy and unhealthy populations, with models typically yielding superior results for healthy subjects [20]. Prior work has demonstrated the demographic sensitivity of foundation models, particularly in vision-language tasks involving chest X-ray classification [38], as well as in large language models applied to medical decision-making [23]. Distributional differences were found between the pretraining cohorts, namely PTB-XL (n = 21,837) and Chapman (n = 10,646) for self-supervised learning; however, downstream model performance was not substantially affected by the choice of the pretraining cohort [17]. Previous studies have primarily focused on interpreting results within specific population subsets [20], applying transfer-learning for model pretrained on one cohort to supervised tasks in another cohort [24,28,38], or testing pretrained models on external cohorts [28]. Comparisons between pretraining cohorts have been limited to just two small datasets, both sourced from secondary care settings [17]. However, to date, there has been no comprehensive investigation into how the characteristics of pretraining cohorts influence the quality of learned representations in medical datasets.

A central contribution of our study lies in its novel comparative analysis of CAPE models pretrained on distinct population cohorts. Our findings reveal that along with the cohort size and the pretraining methodology, the health composition and demographic diversity of the training cohort emerge as pivotal determinants for learning robust features. We assume that the primary-care and volunteer cohorts comprise relatively healthier individuals compared with secondary-care patients, a notion further supported by their higher five-year mortality burden (Table 2). The CAPE models pretrained in secondary care cohorts outperform those pretrained in larger but healthier populations. A plausible explanation for this is that healthier populations tend to exhibit reduced variability, whereas secondary care cohorts encompass a diverse range of cardiac signal patterns. Our contrastive pretraining uses a single pair of ECGs from a patient in each epoch randomly sampled at the training time yet the performance is not solely attributable to the number of patients but also to more ECGs per patient. For example, BIDMC has a smaller number of patients as compared to VUMC but more ECGs per patient, displays a similar performance. This suggests that random sampling of patient ECGs during training can effectively exploit intra-patient ECG diversity. This was further supported by comparison with a strategy that used the same ECG pair across all epochs, which resulted in inferior performance (S4 Table). Pretraining our CAPE foundation model [29] on the large composite BCSV cohort, consistently outperforms models pretrained on individual cohorts (Fig 2). Therefore, we conclude that the effectiveness of contrastive pretraining depends heavily on the diversity, size, and health characteristics of the underlying population.

### Labelled data distribution affects performance evaluation

Next, we address the performance evaluation for comparison between pretraining models. In a number of prior works [14], we find that a task-based comparison is undertaken on diverse cohorts. We observe that the same task across diverse cohort is not comparable as the task complexity greatly depends upon the cohort composition. To further illustrate how the performance is affected by cohort distribution, we examine the UKB cohort, which consistently achieves the highest performance across age and sex prediction for all CAPE models (Fig 2). The UKB stands out due to its narrow interquartile age range (IQR) and its composition as a cohort of healthy volunteers with low five-year mortality (Table 2). Prior studies suggest that models generally perform better on normal ECGs typical of healthy individuals, and struggle more with learning mappings from abnormal cardiac waveforms [20]. Factors such as the cohort’s overall health status, limited age variability, and high data quality likely contribute to the superior model performance observed in this setting. Therefore, performance comparison is only meaningful when evaluated on the same data, ideally using identical train/test splits.

### Combining diverse cohorts for a more effective pretraining of a foundation model with improved generalisation

We explore the impact of pretraining data composition on OOD test performance. Prior work on ECG domain generalisation has explicitly treated data from different hospitals or sources as distinct domains, demonstrating the presence of substantial distributional shifts and corresponding performance degradation under such shifts [28]. Building on this, we evaluate downstream generalisation for our CAPE model trained on diverse cohorts. We trained lightweight MLP networks for target tasks on BIDMC and then externally validated on the rest of cohorts. We observed that combining diverse cohorts for contrastive pretraining significantly reduced the ability of downstream models to generalise to other cohorts (Table 3). We used explainability techniques to understand how the CAPE features map to different cohorts and identified cohort-related clustering (Fig 4A). These patterns aligned more strongly with the ECG recording device than with subject ethnicity (Figs 4B and 4C). Further analysis of ECGs acquired from two devices in CODE (S1–S4 Figs) revealed subtle differences in their frequency spectra that were not visually apparent, and aligning the spectra reduced the observed clustering.

Our in-distribution batch (IDB) strategy rejects learning spurious cohort-specific features but learns more robust features that retain the performance when tested on external cohort. The CAPE model, when trained with batches formed from multiple cohorts, may inadvertently learn cohort-specific signals such as device characteristics, acquisition settings, or preprocessing artefacts. Although not clinically meaningful, these can be exploited during contrastive learning, leading to intra-batch distribution confusion where multiple underlying distributions coexist within a batch and the model relies on spurious correlation features rather than robust physiological patterns, potentially harming generalisation to unseen cohorts. In contrast, CAPE-Z enforces cohort-level consistency within each batch, reducing distributional heterogeneity and limiting the use of cohort-specific artefacts as discriminative shortcuts, thereby promoting more stable and clinically relevant representations. The performance improvement is most pronounced in datasets belonging to the pretraining cohorts, which strengthens the hypothesis that patient contrastive pretraining captures confounding distributional signals. However, a significant though smaller performance gain is also observed in other cohorts. A similar pattern is also seen in fine-tuned models (S5 Table).

The demographic labels of age and sex were selected based on their availability across all cohorts for performance assessment. We further evaluated performance using cardiac labels, including reduced ejection fraction and five-year mortality, in cohorts with available data. The IDB approach outperformed in external validation, and the same performance trends observed for age and sex were also present for these cardiac labels. We also explored sex-stratified performance for five-year mortality and did not observe consistent subgroup-specific differences between CAPE-X and CAPE-Z. This suggests that the improvements associated with the IDB strategy are not driven by demographic subgroup effects but are more likely related to improved robustness to cohort-level distribution shifts. The observed variability across cohorts appears to be influenced by factors such as outcome prevalence and dataset-specific characteristics rather than model bias towards a particular subgroup.

**Fig 6:**
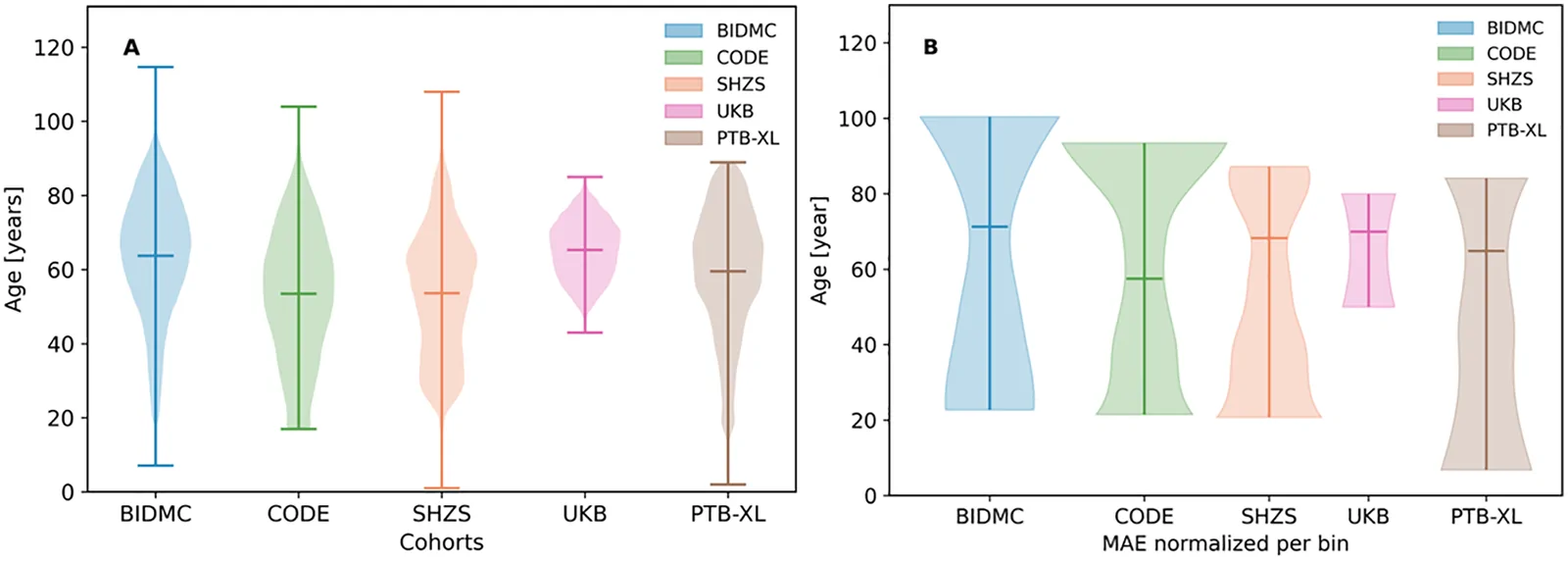
Impact of age distribution on prediction error for CAPE-Z embeddings with a prediction head trained on BIDMC and tested on external cohorts. **A)** Age distributions across different datasets, illustrating mean age and range. **B)** Mean absolute error (MAE) normalized within each age bin, highlighting the age with minimum MAE and the overall age range. The distribution of labels significantly influences the aggregated performance metrics, as error varies across the age spectrum. Notably, the UK Biobank dataset exhibits a narrower age range with distinct peaks corresponding to ages associated with the lowest MAE.

We observe that the IDB demonstrates robust performance for external cohorts (Table 3) but the performance for UKB is still higher than other cohorts. We examine the age distributions across cohorts (Fig 6A) and the mean absolute error (MAE) for age prediction normalised per age bin (Fig 6B). The UKB is not only limited to individuals aged 65–85 but has a skewed distribution with most subjects between 64 and 66 years, interestingly, this also corresponds to the region with the lowest MAE (Fig 6B). These findings suggest that the superior performance of UKB is not solely due to the health status of its participants (as reflected by very low five-year mortality), but also to the over-representation of individuals in the age associated with minimum MAE. This may explain why CODE and SHZS, which also consist of relatively healthy subjects, do not achieve similar performance due to wider age distributions (Fig 5A). By contrast, all-cause mortality prediction in the UKB yields a lower AUC, likely due to high imbalance due to low number of cardiovascular deaths.

In this study, we investigated several critical and compelling questions concerning the generalisation capabilities of our CAPE foundational model. Specifically, we examined the role of cohort composition in shaping the robustness of the pretraining process, the performance of downstream supervised models, and their ability to generalise across diverse settings. Building on these insights, we also propose the IDB strategy aimed at enhancing downstream generalisation. We envision that future research can build upon this direction to further advance the understanding of unbiased performance evaluation and the development of more robust foundation models.

## Limitations

This study aims to investigate how the performance of our contrastive learning methodology is affected by the distributions of the pretraining and label cohorts. While the insights gained are expected to be relevant to other contrastive learning settings, further experimentation is required to validate these findings across different learning paradigms, including supervised learning and alternative foundation model approaches such as masked autoencoders and generative pretraining methods.

Our current work explores pretraining cohorts that differ across multiple complex factors, including ethnicity, socio-economic status, health status, hospital systems, and recording devices. We suggest that the individual contribution of each factor should be systematically investigated in future studies. Our analysis is limited to pretraining strategies, while performance remains influenced by bias in downstream datasets, which could be addressed through more detailed subgroup analyses and improved optimisation of downstream supervised training. We further acknowledge that preprocessing-based strategies may offer a means to mitigate device-specific artefacts. However, exploring such approaches lies beyond the scope of the present study and represents an important avenue for future investigation. Notably, these methods typically require explicit identification of the underlying sources of variation being encoded, which may extend beyond device-related factors. In contrast, IDB does not rely on prior knowledge of these sources.

This study is not intended to provide state-of-the-art label prediction performance across cohorts, but rather to evaluate how supervised training behaves when using features from different pretraining methodologies while keeping labelled dataset sizes comparable. We therefore use random splits of equal size from each cohort for training and validation, based on features extracted from a frozen backbone, to ensure fair comparisons. Training the prediction head on the full cohort was not directly aligned with our primary research objective and future work may establish state-of-the-art performance for each cohort. Another limitation of the current work is that several cohorts used in the study are private datasets and therefore cannot be accessed as open-source resources. While we provide sufficient methodological detail to support replication, the trained model cannot be shared at present owing to the use of private data and ongoing related work.

## Conclusions

This study emphasises the central importance of generalisation in contrastive learning for clinical applications. We find that the ability of a model to perform well across diverse patient cohorts depends critically on the composition of the pretraining data. Specifically, demographic and clinical heterogeneity in the pretraining cohort significantly influences the learned representations and downstream performance. Combining multiple cohorts during contrastive pretraining improves in-distribution performance but reduces out-of-distribution generalisation for our foundation model. We propose In Distribution (ID batch) method to effectively mitigate this degradation. These findings highlight that careful cohort design and evaluation protocols are essential for building foundation models that generalise reliably across diverse clinical populations, ultimately advancing the development of robust machine learning systems in healthcare.

## Materials and methods

### Ethical approvals

All relevant ethical permissions have been obtained for all cohorts explored in the current study. The BIDMC cohort approval is provided by the Beth Israel Deaconess Medical Centre Committee on Clinical Investigations, IRB protocol # 2023P000042. The CODE study is approved by the Research Ethics Committee of the Universidade Federal de Minas Gerais, protocol 49368496317.7.0000.5149. The Institutional Research Board of Zhongshan Hospital (No. 2023-253R) approved the use of SHZS data with a waiver of patient consent. The Vanderbilt component of this study was reviewed and approved by the Institutional Review Board (#212147). The UKB has approval from the Northwest Multi-Centre Research Ethics Committee (application ID 48666). For PTB-XL, the Institutional Ethics Committee approved the publication of the anonymous data in an open-access database (PTB-2020–1).

### Preprocessing

We used leads (I, II, V1-V6), as the remaining four leads (III, aVR, aVF, aVL) do not impart additional information [39]. We applied a bandpass filter (0.5 to 100 Hz) and a notch filter relevant to the mains frequency, re-sampled ECGs from different sources to a standard sampling frequency of 400 Hz on a millivolt scale. The final input to the contrastive learning model was a 7-s signal with dimensions of 2800 × 8. Preprocessing was standardised across all cohorts included in the study.

### Pretraining

#### Architecture.

**Fig 7:**
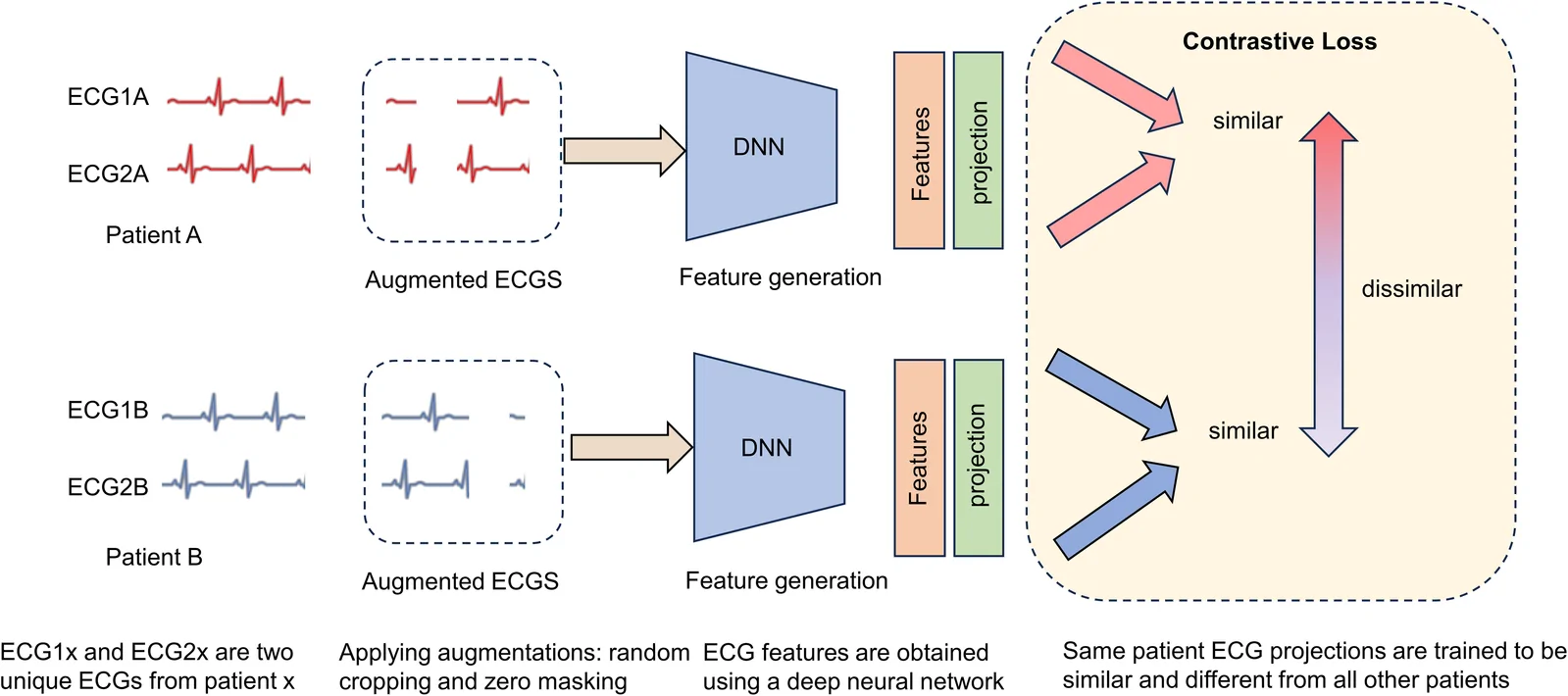
Overview of the CAPE pretraining framework. Patient A has two ECG recordings (ECG1A and ECG2A), and Patient B, another patient in the same batch, has ECG1B and ECG2B. Each ECG undergoes temporal augmentations before being encoded by the network into a 256-dimensional feature vector. The contrastive loss encourages embeddings from the same patient’s ECGs to be closer together while pushing apart embeddings from different patients within the batch.

Fig 7 presents an overview of the CAPE approach. We follow the patient contrastive learning (PCLR) [11], where ECGs from the same patient are treated as positive pairs, while all other ECGs in the batch are treated as negatives. We used a four-layer ResNet (Residual Network) architecture from prior works [11,40] as feature generating backbone. The contrastive loss was applied to the non-linear projections of the ECG embeddings (Fig 7). The resulting 256-dimensional ECG embeddings can subsequently be used for downstream supervised tasks.

#### Contrastive loss.

The contrastive loss employed in this work was the InfoNCE loss [41], applied to nonlinear projections (similar to SimCLR [7]. Given that $z_{i}z_{i}z_{i}$ and $z_{i}z_{j}$ are the non-linear projections of representations from two different augmented ECGs belonging to the same patient, the similarity between $z_{i}$ and $z_{j}$ is maximized over all other instances in the batch under a softmax function [42], over the similarity scores. The loss function is defined as follows in Equation (1):


$$\begin{array}{c} \begin{array}{c} {\text{l}}_{i,j}=-\text{log}\frac{\text{exp}\left(\text{sim}\left(z_{i},z_{j}\right)/\tau \right)}{\sum_{k=\text{1}}^{\text{2}N}I_{\left[k\neq i\right]}\text{exp}\left(\text{sim}\left(z_{i},z_{k}\right)/\tau \right)} \end{array} \end{array}$$ (1)

Here, τ is a temperature parameter controlling the sharpness of the distribution, with a value of 0.1 in the current work. N denotes the number of positive pairs in the batch, and $I_{\left[k\neq i\right]}\in \{\text{0},\text{1}\}$ is an indicator function that evaluates to 1 when k≠i.

The similarity function sim(·,·) is defined using cosine similarity in Equation 2:


$$\begin{array}{c} \begin{array}{c} \text{sim}\left(z_{i},z_{j}\right)=\frac{z_{i}^{⊤}z_{j}}{|z_{i}|\cdot |z_{j}|} \end{array} \end{array}$$ (2)

For batches drawn from a single distribution, we modify the loss as follows. Suppose the training cohort consists of multiple distributions {X,Y,…}. Let $z_{iX}$ and $z_{jX}$ be the projections of augmented ECGs from the same patient, both belonging to the distribution X. As each batch contains samples from only one distribution, the similarity between $z_{iX}$ and $z_{jX}$ is maximized over all other instances in the batch that also belong to distribution X. The modified loss is shown in Equation 3:


$$\begin{array}{c} \begin{array}{c} {\text{l}}_{iX,jX}=-\text{log}\frac{\text{exp}\left(\text{sim}\left(z_{iX},z_{jX}\right)/\tau \right)}{\sum_{k=\text{1}}^{\text{2}N}I_{\left[k\neq i\right]}\text{exp}\left(\text{sim}\left(z_{iX},z_{kX}\right)/\tau \right)} \end{array} \end{array}$$ (3)

The total loss over all batches is given in Equation 4:


$$\begin{array}{c} \text{l}=\sum_{\text{Z}\in \{X,Y,\ldots \}}\sum_{(i,j)\in \text{Z}}{\text{l}}_{i\text{Z},j\text{Z}} \end{array}$$ (4)

### Training

**Fig 8:**
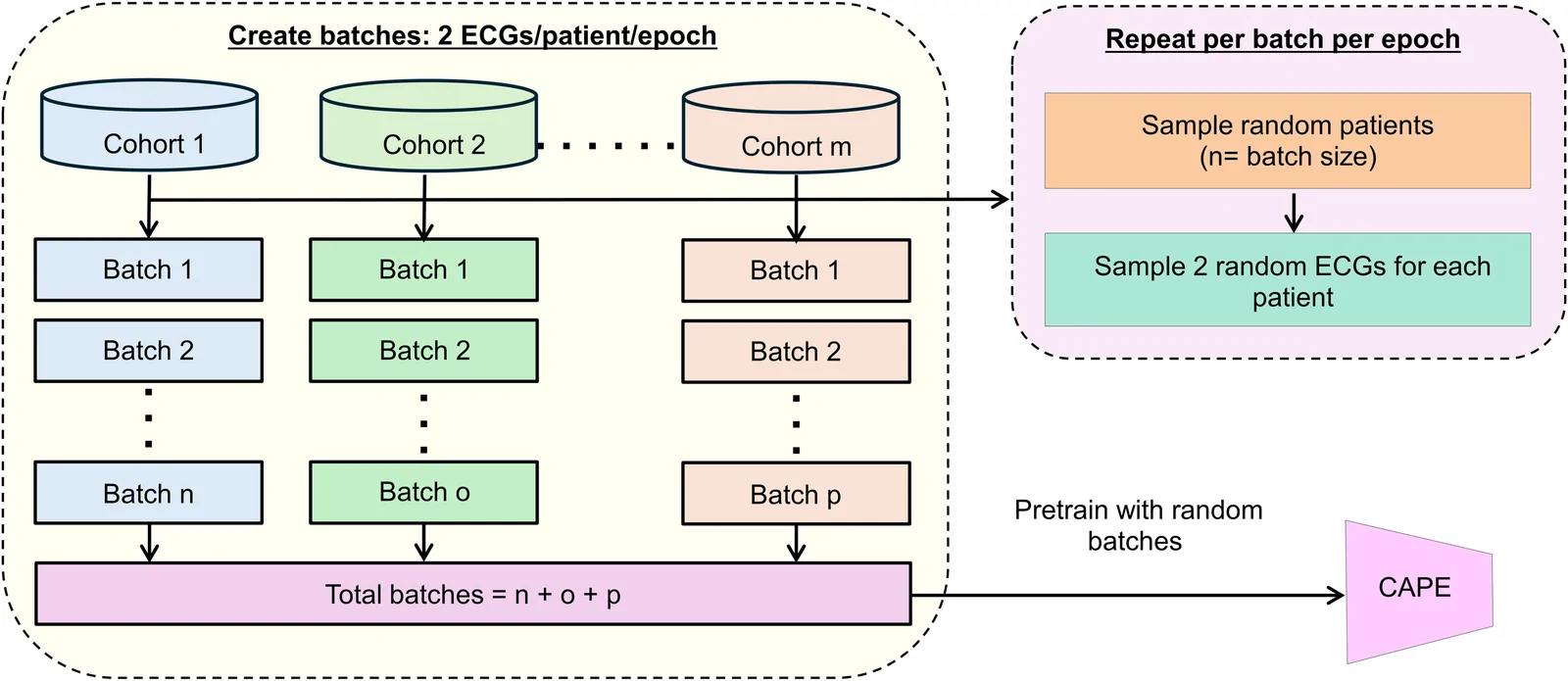
Flowchart of the in-distribution batch (IDB) technique: for each epoch, batches are constructed from the same cohort using two randomly selected ECGs per patient.

During standard CAPE pretraining, batches were randomly sampled across datasets of varying sizes, with each epoch comprising iterations equal to the total number of unique patients. In contrast the in-distribution batch (IDB) constrained each batch to samples drawn from a single dataset. Fig 8 depicts the IDB pretraining procedure: each cohort is partitioned into batches comprising two randomly selected ECG recordings per patient, and batches are randomly sampled during training. The CAPE pretraining randomly samples two ECGs per subject in each epoch, such that different epochs may include different recordings from the same subject.

The contrastive loss performs best with larger batches due to the larger variation of the negative instances [7], but the computation resources limit the batch size. We used a batch size of 1024 (512 patients), as suggested in prior research [11] and trained the model for 200 epochs using the Adam optimiser [43]. The model was implemented in Tensorflow [44] (2.10.1). The initial learning rate was 0.1 and then decayed according to a half-period cosine schedule [45] (similar to previous approaches [11]. The training time in minutes per epoch on NVIDIA GeForce RTX 3090 was approximately 5 for the BIDMC dataset and 50 for the BCSV dataset.

### Prediction head

The prediction head used a multi-layer perceptron (MLP) to learn nonlinear mappings from input features. The MLP architecture was optimised using a grid search over hidden layer sizes of [32, 64, 128, 256] for a two-layer network. For age prediction, the MLP consists of two layers with 256 and 128 neurons, respectively. For sex prediction, both layers contained 256 neurons. The model was trained to predict the target labels using a learning rate of 0.0001, with learning rate decay and early stopping applied to prevent overfitting. The architecture and hyperparameters, including learning rates, were optimised through a limited grid search.

### Performance metrics

Classification performance for sex prediction was evaluated using the area under the receiver operating characteristic curve (AUC). This metric reflects the ability of the model to distinguish between classes across all possible classification thresholds. The AUC is computed by integrating the true positive rate against the false positive rate over varying thresholds. For age regression, model performance was assessed using the mean absolute error (MAE). MAE measures the average absolute difference between predicted and actual values. To compare cohort characteristics and CAPE models pretrained on different datasets, statistical significance was assessed using the Kruskal–Wallis test, with pairwise comparisons corrected using the Holm–Bonferroni method. Comparisons between the In-Distribution Batch (IDB) strategy and training with randomly sampled batches was performed using the Wilcoxon signed-rank test for age prediction (measured by MAE) and the DeLong’s test applied to model prediction scores for sex classification, with each comparison evaluated as a single hypothesis test.

### Code availability

Due to ethical constraints associated with private datasets, the CAPE models used in this study cannot be publicly released. However, to support transparency and reproducibility, we provide a publicly accessible, small-scale working example that demonstrates the key findings of this study. This example utilizes a publicly available subset of the CODE dataset (https://zenodo.org/records/4916206) alongside the PTB-XL dataset and is available at https://github.com/gulrukhk/CAPE-test/.

The example includes an external validation scenario, demonstrating performance both with and without the IDB training procedure. We provide CAPE features extracted from both datasets, together with multilayer perceptrons (MLPs) trained on features originally derived from the BIDMC dataset, for age and sex prediction. In addition, we include supervised learning experiments on the PTB-XL dataset to further demonstrate the effectiveness of the CAPE representations. These experiments comprise age regression, sex classification, and diagnostic classification at both the superclass and subclass levels. Together, the provided example and detailed methodological descriptions are sufficient to reproduce the core results and support the main conclusions of this study.

## Supporting information

S1 Text Supplementary statistical analyses. (DOCX)

S2 Text Exploring device-related artifacts in CODE. (DOCX)

S1 Table Performance comparison of foundation models pretrained using random-batch and in-distribution batch (IDB) strategies, evaluated using the area under the Precision–Recall Gain curve (AUCPRG). The prediction head was trained on BIDMC labels and externally validated on the CODE, SHZS, UKB, and PTB-XL cohorts. Results are reported from ten independent runs using 10,000 randomly selected test samples from each external cohort and are presented as the mean with standard error (SE) in parentheses (e.g., 0.859(019) denotes a mean AUPRG of 0.859 with SE = 0.0019). Models pretrained using IDB generally achieved higher AUPRG than those pretrained with randomly sampled batches, demonstrating improved generalisation to external cohorts.
(DOCX)

S2 Table Sex-stratified performance comparison of foundation models pretrained using random batch and in-distribution batch (IDB) strategies for five-year mortality prediction. AUC (SE) is reported for male and female subgroups, and ΔAUC quantifies the difference in performance between groups. Performance metrics are reported as mean values with standard error (SE) in parentheses, for example 0.805(002) indicates a mean of 0.805 with an SE of 0.002. Overall, sex-related differences are generally small and cohort-dependent, with no consistent pattern favouring either pretraining strategy.
(DOCX)

S3 Table Sex-stratified performance comparison of foundation models pretrained using random batch and in-distribution batch (IDB) strategies for reduced ejection fraction < 40 classification. AUC (SE) is reported for male and female subgroups, and ΔAUC quantifies the difference in performance between groups. Performance metrics are reported as mean values with standard error (SE) in parentheses, for example 0.805(002) indicates a mean of 0.805 with an SE of 0.002. Sex-related differences vary across cohorts, with the largest disparity observed for CAPE-Z in the UKB cohort, indicating that subgroup performance is task- and cohort-dependent rather than consistently associated with the pretraining strategy.
(DOCX)

S4 Table Performance comparison of supervised MLP models trained on BIDMC labels using features from CAPE-B (random ECG pairs per epoch) versus CAPE-B̂ (fixed pair). Performance is reported for age prediction (MAE), sex classification (AUC), and five-year mortality prediction (AUC). Across all downstream tasks, CAPE-B consistently outperforms CAPE-B̂, demonstrating the benefit of exposing the model to diverse ECG pairs during pretraining.
(DOCX)

S5 Table Performance comparison on fine-tuned foundation models pretrained using random-batch and in-distribution batch (IDB) strategies, evaluated using the AUC. The fine-tuning is performed on BIDMC labels across three independent runs, with evaluation conducted on 10,000 random test samples from external cohorts. Performance metrics are reported as mean values with standard errors (SE) in parentheses; for example, a mean absolute error (MAE) of 7.41 with an SE of 0.004 is reported as 7.41(004), and an area under the curve (AUC) of 0.954 with an SE of 0.0002 as 0.954(002). Although fine-tuning improved absolute performance compared with linear probing, it did not recover the reduction in external generalisation associated with random-batch pretraining, with IDB-pretrained models maintaining superior performance across multiple external cohorts.
(DOCX)

S1 Fig Two representative ECGs from the CODE dataset. (A) ECG recorded with device A. (B) ECG recorded with device B. No obvious differences in waveform morphology are observed between the two devices.
(TIF)

S2 Fig Power spectral density (PSD) of ECGs recorded with devices A and B, showing subtle differences, including a small peak around 100 Hz in recordings from device B. (TIF)

S3 Fig Representative ECG before and after spectral transformation. (A) ECG recorded using device A. (B) The same ECG after spectral transformation to match the power spectral density (PSD) of device B. No visible differences in waveform morphology are observed.
(TIF)

S4 Fig Power spectral density (PSD) of an ECG recorded with device A and the same ECG after spectral transformation to match device B. (TIF)

S5 Fig t-SNE components of CAPE embeddings for the CODE dataset. (A) Features from ECGs recorded by the two devices form distinct clusters. (B) Features from ECGs recorded by device A and those from device B transformed to match the spectrum of device A are not separated. (C) Features from ECGs recorded by device B and those from device A transformed to match the spectrum of device B are not separated. (D) Features from ECGs transformed to match the spectrum of the other device again form separate clusters.
(TIF)

S6 Fig Loss comparison for CAPE-B trained with random ECG pairs per epoch for each patient versus a model trained with a fixed random pair (CAPE-B̂). Losses are similar initially but diverge after ~50 epochs, with CAPE-B continuing to decrease while the fixed-pair model saturates.
(TIF)
