# Supplementary material for: Data distribution impacts the performance and generalisability of contrastive learning-based foundation models of electrocardiograms
Source: PLOS Digit Health. 2026 Sep 2;5(9):e0001623. doi: 10.1371/journal.pdig.0001623 (PMC13537565; doi:10.1371/journal.pdig.0001623)
Supplement: Supplementary file 1 [file pdig.0001623.s001.docx]

## S1 Text. Supplementary Statistical Analyses

### This supplementary text provides the detailed pairwise statistical analyses supporting the results presented in the section "Composition of Pretraining Cohort Affects Performance." Statistical significance was assessed using the Kruskal–Wallis test, followed by pairwise comparisons with Holm–Bonferroni correction for multiple testing.

### BIDMC MAE age:

Kruskal-Wallis: H=23.449, p=0.0001

CAPE-B vs CAPE-C: raw p=0.0039, corrected p=0.0395

CAPE-B vs CAPE-S: raw p=0.0039, corrected p=0.0395

CAPE-B vs CAPE-V: raw p=0.0374, corrected p=0.1495

CAPE-B vs CAPE-X: raw p=0.3367, corrected p=0.5247

CAPE-C vs CAPE-S: raw p=0.2623, corrected p=0.5247

CAPE-C vs CAPE-V: raw p=0.0039, corrected p=0.0395

CAPE-C vs CAPE-X: raw p=0.0039, corrected p=0.0395

CAPE-S vs CAPE-V: raw p=0.0039, corrected p=0.0395

CAPE-S vs CAPE-V: raw p=0.0039, corrected p=0.0395

CAPE-V vs CAPE-V: raw p=0.0374, corrected p=0.1495

### CODE MAE age:

Kruskal-Wallis: H=21.763, p=0.0002

CAPE-B vs CAPE-C: raw p=0.0065, corrected p=0.0395

CAPE-B vs CAPE-S: raw p=0.6310, corrected p=1.0000

CAPE-B vs CAPE-V: raw p=0.2623, corrected p=0.7870

CAPE-B vs CAPE-X: raw p=0.0039, corrected p=0.0395

CAPE-C vs CAPE-S: raw p=0.0039, corrected p=0.0395

CAPE-C vs CAPE-V: raw p=0.0104, corrected p=0.0416

CAPE-C vs CAPE-X raw p=0.0065, corrected p=0.0395

CAPE-S vs CAPE-V: raw p=0.5218, corrected p=1.0000

CAPE-V vs CAPE-V: raw p=0.0039, corrected p=0.0395

CAPE-V vs CAPE-X: raw p=0.0039, corrected p=0.0395

### SHZS MAE age:

Kruskal-Wallis: H=21.918, p=0.0002

CAPE-B vs CAPE-C: raw p=0.3367, corrected p=1.0000

CAPE-B vs CAPE-S: raw p=0.0039, corrected p=0.0395

CAPE-B vs CAPE-V: raw p=0.4233, corrected p=1.0000

CAPE-B vs CAPE-X: raw p=0.0039, corrected p=0.0395

CAPE-C vs CAPE-S: raw p=0.0039, corrected p=0.0395

CAPE-C vs CAPE-V: raw p=0.1495, corrected p=0.5982

CAPE-C vs CAPE-X: raw p=0.0039, corrected p=0.0395

CAPE-S vs CAPE-V: raw p=0.0039, corrected p=0.0395

CAPE-S vs CAPE-X: raw p=0.4233, corrected p=1.0000

CAPE-V vs CAPE-X: raw p=0.0039, corrected p=0.0395

### UKB MAE age:

Kruskal-Wallis: H=22.189, p=0.0002

CAPE-B vs CAPE-C: raw p=0.0374, corrected p=0.1495

CAPE-B vs CAPE-S: raw p=0.0039, corrected p=0.0395

CAPE-B vs CAPE-V: raw p=0.4233, corrected p=0.4233

CAPE-B vs CAPE-X: raw p=0.0104, corrected p=0.0624

CAPE-C vs CAPE-S: raw p=0.0250, corrected p=0.1249

CAPE-C vs CAPE-V: raw p=0.0374, corrected p=0.1495

CAPE-C vs CAPE-X: raw p=0.0039, corrected p=0.0395

CAPE-S vs CAPE-V: raw p=0.0039, corrected p=0.0395

CAPE-S vs CAPE-X: raw p=0.0039, corrected p=0.0395

CAPE-V vs CAPE-X: raw p=0.0374, corrected p=0.1495

### PTB-XL MAE age:

Kruskal-Wallis: H=21.966, p=0.0002

CAPE-B vs CAPE-C: raw p=0.0039, corrected p=0.0395

CAPE-B vs CAPE-S: raw p=0.0039, corrected p=0.0395

CAPE-B vs CAPE-V: raw p=0.6310, corrected p=1.0000

CAPE-B vs CAPE-X: raw p=0.4233, corrected p=1.0000

CAPE-C vs CAPE-S: raw p=0.0547, corrected p=0.2187

CAPE-C vs CAPE-V: raw p=0.0039, corrected p=0.0395

CAPE-C vs CAPE-X: raw p=0.0039, corrected p=0.0395

CAPE-S vs CAPE-V: raw p=0.0039, corrected p=0.0395

CAPE-S vs CAPE-X: raw p=0.0039, corrected p=0.0395

CAPE-V vs CAPE-X: raw p=0.3367, corrected p=1.0000

### Mean MAE of all cohorts for Age:

Kruskal-Wallis: H=22.482, p=0.0002

CAPE-B vs CAPE-C: raw p=0.0039, corrected p=0.0395

CAPE-B vs CAPE-S: raw p=0.4233, corrected p=1.0000

CAPE-B vs CAPE-V: raw p=0.6310, corrected p=1.0000

CAPE-B vs CAPE-X: raw p=0.0039, corrected p=0.0395

CAPE-C vs CAPE-S: raw p=0.0065, corrected p=0.0395

CAPE-C vs CAPE-V: raw p=0.0039, corrected p=0.0395

CAPE-C vs CAPE-X: raw p=0.0039, corrected p=0.0395

CAPE-S vs CAPE-V: raw p=0.3367, corrected p=1.0000

CAPE-S vs CAPE-X: raw p=0.0039, corrected p=0.0395

CAPE-V vs CAPE-X: raw p=0.0039, corrected p=0.0395

### BIDMC Sex AUC:

Kruskal-Wallis: H=27.097, p=0.0000

CAPE-B vs CAPE-C: raw p=0.0039, corrected p=0.0395

CAPE-B vs CAPE-S: raw p=0.0039, corrected p=0.0395

CAPE-B vs CAPE-V: raw p=0.0547, corrected p=0.0547

CAPE-B vs CAPE-X: raw p=0.0039, corrected p=0.0395

CAPE-C vs CAPE-S: raw p=0.0039, corrected p=0.0395

CAPE-C vs CAPE-V: raw p=0.0039, corrected p=0.0395

CAPE-C vs CAPE-X: raw p=0.0039, corrected p=0.0395

CAPE-S vs CAPE-V: raw p=0.0039, corrected p=0.0395

CAPE-S vs CAPE-X: raw p=0.0039, corrected p=0.0395

CAPE-V vs CAPE-X: raw p=0.0039, corrected p=0.0395

### CODE Sex AUC:

Kruskal-Wallis: H=27.871, p=0.0000

CAPE-B vs CAPE-C: raw p=0.0039, corrected p=0.0395

CAPE-B vs CAPE-S: raw p=0.0039, corrected p=0.0395

CAPE-B vs CAPE-V: raw p=0.0039, corrected p=0.0395

CAPE-B vs CAPE-X: raw p=0.0039, corrected p=0.0395

CAPE-C vs CAPE-S: raw p=0.0039, corrected p=0.0395

CAPE-C vs CAPE-V: raw p=0.0039, corrected p=0.0395

CAPE-C vs CAPE-X: raw p=0.0039, corrected p=0.0395

CAPE-S vs CAPE-V: raw p=0.0039, corrected p=0.0395

CAPE-S vs CAPE-X: raw p=0.0039, corrected p=0.0395

CAPE-V vs CAPE-X: raw p=0.0039, corrected p=0.0395

### SHZS Sex AUC:

Kruskal-Wallis: H=24.017, p=0.0001

CAPE-B vs CAPE-C: raw p=0.0547, corrected p=0.1640

CAPE-B vs CAPE-S: raw p=0.0039, corrected p=0.0395

CAPE-B vs CAPE-V: raw p=0.2002, corrected p=0.4004

CAPE-B vs CAPE-X: raw p=0.0039, corrected p=0.0395

CAPE-C vs CAPE-S: raw p=0.0039, corrected p=0.0395

CAPE-C vs CAPE-V: raw p=0.2002, corrected p=0.4004

CAPE-C vs CAPE-X: raw p=0.0039, corrected p=0.0395

CAPE-S vs CAPE-V: raw p=0.0039, corrected p=0.0395

CAPE-S vs CAPE-X: raw p=0.0039, corrected p=0.0395

CAPE-V vs CAPE-X: raw p=0.0039, corrected p=0.0395

### UKB Sex AUC:

Kruskal-Wallis: H=26.843, p=0.0000

CAPE-B vs CAPE-C: raw p=0.0039, corrected p=0.0395

CAPE-B vs CAPE-S: raw p=0.0039, corrected p=0.0395

CAPE-B vs CAPE-V: raw p=0.0163, corrected p=0.0395

CAPE-B vs CAPE-X: raw p=0.0039, corrected p=0.0395

CAPE-C vs CAPE-S: raw p=0.0039, corrected p=0.0395

CAPE-C vs CAPE-V: raw p=0.0039, corrected p=0.0395

CAPE-C vs CAPE-X: raw p=0.0039, corrected p=0.0395

CAPE-S vs CAPE-V: raw p=0.0039, corrected p=0.0395

CAPE-S vs CAPE-X: raw p=0.0039, corrected p=0.0395

CAPE-V vs CAPE-X: raw p=0.0250, corrected p=0.0395

### PTB-XL Sex AUC:

Kruskal-Wallis: H=27.578, p=0.0000

CAPE-B vs CAPE-C: raw p=0.0039, corrected p=0.0395

CAPE-B vs CAPE-S: raw p=0.0039, corrected p=0.0395

CAPE-B vs CAPE-V: raw p=0.0104, corrected p=0.0395

CAPE-B vs CAPE-X: raw p=0.0039, corrected p=0.0395

CAPE-C vs CAPE-S: raw p=0.0039, corrected p=0.0395

CAPE-C vs CAPE-V: raw p=0.0039, corrected p=0.0395

CAPE-C vs CAPE-X: raw p=0.0039, corrected p=0.0395

CAPE-S vs CAPE-V: raw p=0.0039, corrected p=0.0395

CAPE-S vs CAPE-X: raw p=0.0039, corrected p=0.0395

CAPE-V vs CAPE-X: raw p=0.0039, corrected p=0.0395

### Mean AUC Sex:

Kruskal-Wallis: H=26.826, p=0.0000

CAPE-B vs CAPE-C: raw p=0.0039, corrected p=0.0395

CAPE-B vs CAPE-S: raw p=0.0039, corrected p=0.0395

CAPE-B vs CAPE-V: raw p=0.1495, corrected p=0.1495

CAPE-B vs CAPE-X: raw p=0.0039, corrected p=0.0395

CAPE-C vs CAPE-S: raw p=0.0039, corrected p=0.0395

CAPE-C vs CAPE-V: raw p=0.0039, corrected p=0.0395

CAPE-C vs CAPE-X: raw p=0.0039, corrected p=0.0395

CAPE-S vs CAPE-V: raw p=0.0039, corrected p=0.0395

CAPE-S vs CAPE-X: raw p=0.0039, corrected p=0.0395

CAPE-V vs CAPE-X: raw p=0.0039, corrected p=0.0395
