# Supplementary material for: Data distribution impacts the performance and generalisability of contrastive learning-based foundation models of electrocardiograms
Source: PLOS Digit Health. 2026 Sep 2;5(9):e0001623. doi: 10.1371/journal.pdig.0001623 (PMC13537565; doi:10.1371/journal.pdig.0001623)
Supplement: Supplementary file 2 [file pdig.0001623.s002.docx]

## S2 Text. Exploring Device-related Artifacts in CODE:

The t-SNE plots in Fig 4C (main paper) show that CAPE embeddings from the CODE cohort form two distinct clusters corresponding to the two ECG recording devices. To investigate the source of this separation, we performed additional analyses. Visual inspection of representative ECGs from both devices revealed no obvious differences (S1 Fig). However, power spectral density (PSD) analysis identified subtle frequency-domain differences, including a small peak around 100 Hz in recordings from device B (S2 Fig).

To determine whether these spectral differences explain the observed clustering, we derived transformations that modify ECG signals so that the PSD of one device matches that of the other. S3 Fig compares a representative ECG recorded with device A before and after spectral transformation, showing no visible differences between the two signals. S4 Fig shows that the transformation successfully matches the PSD of the target device while preserving the ECG waveform.

We then generated CAPE embeddings for the transformed ECGs and repeated the t-SNE analysis. S5 Fig shows that embeddings of ECGs recorded using the two devices form distinct clusters (panel A). When ECGs from one device were transformed to match the PSD of the other, this separation disappeared (panels B and C). However, when ECGs from both devices were transformed to match the PSD of the opposite device, the embeddings again segregated according to device (panel D). Together, these findings indicate that the clustering observed in Fig 4C is primarily driven by subtle device-specific differences in the frequency spectrum rather than visible differences in ECG morphology.
