# Supplementary material for: Data distribution impacts the performance and generalisability of contrastive learning-based foundation models of electrocardiograms
Source: PLOS Digit Health. 2026 Sep 2;5(9):e0001623. doi: 10.1371/journal.pdig.0001623 (PMC13537565; doi:10.1371/journal.pdig.0001623)
Supplement: Supplementary file 3 [file pdig.0001623.s003.docx]

S1 Table. **Performance comparison of foundation models pretrained using random-batch and in-distribution batch (IDB) strategies, evaluated using the area under the Precision–Recall Gain curve (AUCPRG).** The prediction head was trained on BIDMC labels and externally validated on the CODE, SHZS, UKB, and PTB-XL cohorts. Results are reported from ten independent runs using 10,000 randomly selected test samples from each external cohort and are presented as the mean with standard error (SE) in parentheses (e.g., 0.859(019) denotes a mean AUPRG of 0.859 with SE = 0.0019). Models pretrained using IDB generally achieved higher AUPRG than those pretrained with randomly sampled batches, demonstrating improved generalisation to external cohorts.

| No. | Model | BIDMC | CODE | SHZS | UKB | PTB-XL | mean |
| --- | --- | --- | --- | --- | --- | --- | --- |
| Sex prediction (AUPRG) | | | | | | | |
| 1 | CAPE-X | **0.859(019)** | -1.63(517) | 0.497(668) | 0.939(022) | 0.854(016) | 0.669 |
| 2 | CAPE-Z | 0.851(023) | **0.792(037)** | **0.939(016)** | **0.946(013)** | **0.875(013)** | **0.879** |
| 3 | p-value^$^ | <0.0001 | <0.0001 | 0.0002 | 0.0291 | <0.0001 | **-** |
| LVEF < 40 ( AUPRG ) | | | | | | | |
| 4 | CAPE-X | 0.849(025) | - | 0.289(334) | **0.850(106)** | - | 0.663 |
| 5 | CAPE-Z | **0.851(025)** | - | **0.959(015)** | 0.844(014) | - | **0.885** |
| 6 | p-value^$^ | 0.0255 | - | <0.0001 | 0.3183 | - | - |
| 5 year mortality ( AUPRG ) | | | | | | | |
| 7 | CAPE-X | 0.572(072) | 0.412(331) | - | **0.437(132)** | - | 0.483 |
| 8 | CAPE-Z | **0.617(033)** | **0.642(068)** | - | 0.424(058) | - | **0.547** |
| 9 | p-value^$^ | <0.0001 | 0.0001 | - | 0.2939 | - | - |

* Out of range with SE(MAE) ≥ 1.0 or SE(AUC) ≥ 0.01.

^!^ Wilcoxon signed-rank test

^$^ DeLong test
