# Supplementary material for: Data distribution impacts the performance and generalisability of contrastive learning-based foundation models of electrocardiograms
Source: PLOS Digit Health. 2026 Sep 2;5(9):e0001623. doi: 10.1371/journal.pdig.0001623 (PMC13537565; doi:10.1371/journal.pdig.0001623)
Supplement: Supplementary file 4 [file pdig.0001623.s004.docx]

S2 Table. **Sex-stratified performance comparison of foundation models pretrained using random batch and in-distribution batch (IDB) strategies for five-year mortality prediction.** AUC (SE) is reported for male and female subgroups, and ΔAUC quantifies the difference in performance between groups. Performance metrics are reported as mean values with standard error (SE) in parentheses, for example 0.805(002) indicates a mean of 0.805 with an SE of 0.002. Overall, sex-related differences are generally small and cohort-dependent, with no consistent pattern favouring either pretraining strategy.

| No. | Dataset | Model | Male AUC | Female AUC | ΔAUC | p |
| --- | --- | --- | --- | --- | --- | --- |
| 1. | BIDMC (male =52%) | CAPE-X | 0.805 (002) | 0.803 (002) | 0.002 (003) | 0.496 |
| 2. |  | CAPE-Z | **0.823 (002)** | **0.823 (002)** | **0.000 (002)** | **0.734** |
| 3. | CODE  (male =43%) | CAPE-X | 0.695 (013) | 0.686 (015) | **0.009 (007)** | **0.496** |
| 4. |  | CAPE-Z | **0.806 (003)** | **0.796 (004)** | 0.011 (003) | 0.008 |
| 5. | UKB  (male =50%) | CAPE-X | **0.680 (005)** | 0.627 (008) | 0.052 (009) | 0.004 |
| 6. |  | CAPE-Z | 0.676 (003) | **0.632 (005)** | **0.042 (006)** | **0.004** |
