# Supplementary material for: Data distribution impacts the performance and generalisability of contrastive learning-based foundation models of electrocardiograms
Source: PLOS Digit Health. 2026 Sep 2;5(9):e0001623. doi: 10.1371/journal.pdig.0001623 (PMC13537565; doi:10.1371/journal.pdig.0001623)
Supplement: Supplementary file 5 [file pdig.0001623.s005.docx]

S3 Table. **Sex-stratified performance comparison of foundation models pretrained using random batch and in-distribution batch (IDB) strategies for reduced ejection fraction < 40 classification.** AUC (SE) is reported for male and female subgroups, and ΔAUC quantifies the difference in performance between groups. Performance metrics are reported as mean values with standard error (SE) in parentheses, for example 0.805(002) indicates a mean of 0.805 with an SE of 0.002. Sex-related differences vary across cohorts, with the largest disparity observed for CAPE-Z in the UKB cohort, indicating that subgroup performance is task- and cohort-dependent rather than consistently associated with the pretraining strategy.

| No. | Dataset | Model | Male AUC | Female AUC | ΔAUC | p |
| --- | --- | --- | --- | --- | --- | --- |
| 1. | BIDMC (male =52%) | CAPE-X | **0.894 (002)** | 0.886 (004) | **0.006 (004)** | **0.203** |
| 2. |  | CAPE-Z | 0.884 (002) | **0.888 (002)** | **0.006 (004)** | **0.250** |
| 3. | SHZS  (male =55%) | CAPE-X | 0.567 (018) | 0.604 (013) | 0.037 (013) | 0.020 |
| 4. |  | CAPE-Z | **0.964 (002)** | **0.972 (003)** | **0.008 (004)** | **0.074** |
| 5. | UKB  (male =48%) | CAPE-X | 0.866 (005) | **0.827 (017)** | **0.039 (019)** | **0.074** |
| 6. |  | CAPE-Z | **0.877 (008)** | 0.726 (014) | 0.151 (015) | 0.004 |
