# Supplementary material for: Data distribution impacts the performance and generalisability of contrastive learning-based foundation models of electrocardiograms
Source: PLOS Digit Health. 2026 Sep 2;5(9):e0001623. doi: 10.1371/journal.pdig.0001623 (PMC13537565; doi:10.1371/journal.pdig.0001623)
Supplement: Supplementary file 6 [file pdig.0001623.s006.docx]

**S4 Table. Performance comparison of supervised MLP models trained on BIDMC labels using features from CAPE-B (random ECG pairs per epoch) versus CAPE-B̂ (fixed pair). Performance is reported for age prediction (MAE), sex classification (AUC), and five-year mortality prediction (AUC). Across all downstream tasks, CAPE-B consistently outperforms CAPE-B̂, demonstrating the benefit of exposing the model to diverse ECG pairs during pretraining.**

| No. | Model | Age (MAE) | Sex (AUC) | Mortality 5 year (AUC) |
| --- | --- | --- | --- | --- |
| 1 | CAPE-B | **7.95** | **0.927** | **0.854** |
| 2 | CAPE-B̂ | 8.39 | 0.903 | 0.815 |
