# Supplementary material for: Data distribution impacts the performance and generalisability of contrastive learning-based foundation models of electrocardiograms
Source: PLOS Digit Health. 2026 Sep 2;5(9):e0001623. doi: 10.1371/journal.pdig.0001623 (PMC13537565; doi:10.1371/journal.pdig.0001623)
Supplement: Supplementary file 7 [file pdig.0001623.s007.docx]

S5 Table. **Performance comparison on fine-tuned foundation models pretrained using random-batch and in-distribution batch (IDB) strategies, evaluated using the AUC.** The fine-tuning is performed on BIDMC labels across three independent runs, with evaluation conducted on 10,000 random test samples from external cohorts. Performance metrics are reported as mean values with standard errors (SE) in parentheses; for example, a mean absolute error (MAE) of 7.41 with an SE of 0.004 is reported as 7.41(004), and an area under the curve (AUC) of 0.954 with an SE of 0.0002 as 0.954(002). Although fine-tuning improved absolute performance compared with linear probing, it did not recover the reduction in external generalisation associated with random-batch pretraining, with IDB-pretrained models maintaining superior performance across multiple external cohorts.

| No. | Model | BIDMC | CODE | SHZS | UKB | PTB-XL | mean |
| --- | --- | --- | --- | --- | --- | --- | --- |
| Age prediction (MAE) | | | | | | | |
| 1 | CAPE-X | **7.41(004)** | 30.94(*) | 12.15(*) | **5.77(016)** | 7.22(051) | 12.70 |
| 2 | CAPE-Z | 7.44(003) | **7.95(047)** | **7.42(022)** | 5.83(015) | **7.03(029)** | **7.13** |
| 3 | p-value^!^ | 0.919 | <0.0001 | <0.0001 | <0.0001 | 0.063 | **-** |
| Sex prediction (AUC) | | | | | | | |
| 4 | CAPE-X | 0.954(025) | 0.715(*) | 0.900(104) | 0.980(008) | 0.934(013) | 0.896 |
| 5 | CAPE-Z | **0.955(000)** | **0.931(000)** | **0.955(001)** | **0.983(000)** | **0.938(001)** | **0.952** |
| 6 | p-value^$^ | 0.038 | <0.0001 | <0.0001 | 0.0132 | 0.002 |  |

* Out of range with SE(MAE) ≥ 1.0 or SE(AUC) ≥ 0.01.

^!^ Wilcoxon signed-rank test on sample-wise MAE

^$^ DeLong test
