# Supplementary material for: Data distribution impacts the performance and generalisability of contrastive learning-based foundation models of electrocardiograms
Source: PLOS Digit Health. 2026 Sep 2;5(9):e0001623. doi: 10.1371/journal.pdig.0001623 (PMC13537565; doi:10.1371/journal.pdig.0001623)
Supplement: Supplementary file 8 [file pdig.0001623.s015.docx]

**Manuscript: RE: PDIG-D-26-00170**

**Data distribution impacts the performance and generalisability of contrastive learning-based foundation models of electrocardiograms**

We thank the reviewers and editors for their expert opinion, detailed commentary and in-depth review of our manuscript. The detailed feedback has been imperative in improving the manuscript in this submission. We have addressed all of the comments and provided a detailed point-by point response and highlighted changes to the manuscript in response to these comments (Page and Line numbers below refer to the revised manuscript).

We hope that our changes have sufficiently addressed the reviewers’ comments to allow for the publication of our manuscript.

**Editor Comments: Summary:**

**This paper has been review by 5 independent reviewers with suggests of 3 major and 2 minor revisions. All reviewers acknowledge the manuscript’s strengths, including its large-scale multi-cohort dataset, the systematic investigation of cohort composition in ECG foundation model pretraining, and the proposed IDB strategy to improve out-of-distribution (OOD) generalization. The topic is timely and potentially impactful. However, the reviewers identified several substantial concerns that require major revision before the manuscript can be considered further. The major revision points are summarized below.**

We sincerely thank the reviewers for recognizing the strengths of our work and its potential impact. We are also grateful for their thoughtful and constructive feedback, which has significantly helped us improve the quality and clarity of the manuscript. In the following, we address each of the points raised for major revision in detail.

**1. Expand downstream evaluation to include clinically meaningful ECG tasks. (Reviewers 3, 4, 5)**

We thank the editors and reviewers for this important suggestion. While clinically meaningful tasks (reduced left ventricular ejection fraction and five-year mortality) were already included in the study, they were not sufficiently highlighted in the original manuscript. We have revised the manuscript to clearly introduce these endpoints from the outset and emphasise their role in supporting the clinical relevance of our findings, alongside age and sex tasks used for consistent cross-cohort evaluation. Further details are provided in our responses to Reviewers 2, 3, 4, and 5.

**2. Assess the effect of backbone fine-tuning (or justify why frozen-feature evaluation is sufficient). (Reviewers 4, 5)**

We clarified that downstream evaluation is performed using frozen backbone features with an MLP prediction head, following a standard linear-probe-style evaluation commonly used in self-supervised learning. Using frozen features isolates representation quality from downstream task-specific optimisation and enables controlled comparison of pretraining strategies across cohorts and tasks. We further emphasised that, in many practical applications of foundation models, frozen feature extraction is commonly used as a simple and reliable deployment strategy. Our goal is therefore to evaluate learned representations rather than fully optimised downstream performance, and frozen-feature evaluation provides a computationally efficient and practically relevant setting. For completeness, we also investigate fine-tuning in the generalisation experiments in the Supplementary Information and find similar trends to the linear-probe results. Further details and manuscript changes are provided in responses to Reviewers 2, 4 and 5.

**3. Provide deeper analysis and explanation of the IDB strategy, including subgroup analysis and device-artifact considerations. (Reviewers 1, 4)**

We clarified the IDB strategy with an additional figure (Fig. 7) and a more detailed description of its formulation. IDB constrains contrastive pretraining to within-cohort batches, unlike standard CAPE training which mixes cohorts, to reduce reliance on cohort-specific shortcuts that can harm cross-cohort generalisation (supported by t-SNE clustering in Fig. 4 and 5).

We also added subgroup and artifact analyses in the supplementary information showing that cohort clustering is not explained by ethnicity (BIDMC) but is associated with device differences within CODE. These effects persist despite identical preprocessing and spectral alignment, suggesting sensitivity to subtle acquisition-related signals. Further details are provided in responses to Reviewers 1 and 4.

Overall, these additions support our interpretation that IDB encourages learning more transferable representations by reducing cohort-specific bias.

**4. Strengthen statistical evaluation, including full-cohort analyses where possible and clarification of multiple-comparison corrections. (Reviewers 1, 2)**

We thank the reviewers for this important suggestion. We would like to clarify that the primary objective of this work was not to establish state-of-the-art performance for individual cohorts, but rather to enable controlled comparisons across pretraining strategies. We therefore used randomly sampled subsets of equal size for all downstream evaluations to minimise bias arising from differences in cohort size. Repeated random sampling also provides a more robust assessment of model performance and reduces sensitivity to a single train–validation partition.

We have strengthened the statistical evaluation by incorporating Kruskal–Wallis tests followed by Holm–Bonferroni-corrected post hoc pairwise comparisons to account for multiple testing. In addition, the Methods section has been clarified to specify where multiple-comparison correction was applied (cohort-level comparisons) and where single-hypothesis tests were used (Wilcoxon and DeLong tests), together with the corresponding rationale, as detailed in our response to Reviewer 1. Additional pairwise statistical results have also been included in the Supplementary Material.

**5. Clarify dataset definitions and cohort characteristics, particularly the definition of “healthy populations.” (Reviewer 1)**

We thank Reviewer 1 for this suggestion. We have clarified the definition of “healthy populations” and incorporated 5-year mortality as a quantitative measure of cohort health status. We observe substantially higher mortality rates in secondary care cohorts (approximately 32–35%) compared to primary care and population-based cohorts (approximately 9–16% in CODE and 0.4% in UKB). These values are now reported in Tables 1 and 2, and corresponding text has been added to the manuscript. Details are provided in response to Reviewer 1.

**6. Situate the work within broader foundation model research, including other pretraining strategies. (Reviewers 3, 5)**

We thank the reviewers for this helpful suggestion and have revised the manuscript to better situate our work within the broader foundation model literature. Specifically, we added changes in the Introduction and Discussion sections to include a more comprehensive overview of related pretraining strategies and self-supervised learning approaches. These revisions clarify how our method relates to existing foundation model frameworks and highlight its distinction in explicitly addressing cohort structure during pretraining to improve cross-cohort generalisation. Further details are provided in responses to Reviewers 3 and 5.

**7. Improve reproducibility and accessibility of the model or implementation details where possible. (Reviewer 1)**

We agree with the reviewer that reproducibility and accessibility are important. We have therefore added further methodological detail, including a schematic of the IDB training pipeline (Fig. 8), to facilitate replication.

To support our main findings, we additionally provide a public test repository (<https://github.com/gulrukhk/CAPE-test>), which includes extracted features (with and without IDB) for the open cohorts CODE15 and PTB-XL, along with scripts for training and evaluating downstream MLP models. We also demonstrate external validation by training on BIDMC and testing on CODE15 and PTB-XL, illustrating generalisation differences across cohorts.

While the full trained model cannot be released at this stage due to the use of private datasets, we acknowledge this as a limitation and have explicitly stated it in the manuscript. We believe that the combination of detailed methodological description, released code, and shared feature representations provides sufficient support for reproducibility and enables further research on the included public cohorts. Further details on manuscript changes are provided in response to Reviewer 1.

**8. Provide explicit fairness analyses if claims of clinical fairness are maintained. (Reviewer 1)**

We thank the reviewer for this important comment. We clarify that we do not claim end-to-end clinical fairness; rather, our focus is on how pretraining strategies influence downstream generalisation in clinically deployable and robust settings. While the learned representations may improve robustness, we acknowledge that residual biases in downstream supervised models remain and are not fully addressed in this work. A comprehensive fairness analysis is beyond the scope of the present study and represents an important direction for future research. To avoid ambiguity, we have revised the manuscript to remove this claim and to clarify our intended scope. Further details about all changes are provided in response to Reviewer 1.

**9. Substantially improve figures, organization, and language quality throughout the manuscript. (Reviewers 1, 2)**

We improved figure quality by increasing resolution, enhancing colour contrast, and refining annotations. The manuscript was also carefully revised for language clarity, grammar, and overall organisation and flow.

Specifically, we split the original Figure 4 into two figures for improved interpretability: revised Figure 4 presents t-SNE embeddings across cohorts, highlighting stronger associations with technical factors such as recording devices rather than clinical attributes such as ethnicity, while Figure 5 shows that IDB-learned representations reduce such clustering and resemble single-cohort training.

We also standardised all figures by replacing screenshots with high-resolution TIFF images and harmonising font sizes and formatting. Further details are provided in responses to Reviewers 1 and 2.

**10. Investigate device-related artifacts and their relationship to representation clustering (Reviewers 1, 4).**

We thank the reviewers for this suggestion. We agree that interpretation of the t-SNE embeddings requires careful analysis. During training across multiple datasets, we observed a substantial drop in performance during external validation on certain cohorts. To better understand this behaviour, we visualised the learned representations using t-SNE (Fig. 4A), which revealed clear clustering by dataset. Further analysis showed that these clusters were not associated with demographic variables such as ethnicity (Fig. 4B), but were strongly linked to technical factors, in particular differences in recording devices within the CODE cohort (Fig. 4C). Visual inspection of the ECG waveforms did not reveal any obvious differences between devices.

To further investigate this, we conducted additional analyses in Supplementary Information. In the frequency domain, we observed subtle but consistent differences in the power spectral density (PSD) between devices. To assess whether these differences explain the observed clustering, we transformed ECG signals from device A to match the PSD of device B, and vice versa. When t-SNE embeddings were recomputed on features derived from these spectrally matched signals, the device-based clustering largely disappeared. Overall, these results indicate that the learned representations capture device-specific information that is at least partially related to PSD differences, and that such technical artifacts can meaningfully influence the structure of the feature space. Further details are provided in responses to Reviewers 1 and 4.

**Reviewer 1:**

**The article demonstrates innovation and practicality in its methodological design, experimental scale, and research questions. However, despite its high overall quality, several shortcomings remain, which are recommended for improvement during the revision process:**

We sincerely thank the reviewer for the positive assessment of our work and for recognizing its methodological rigor and practical relevance. We are also grateful for the time and effort invested in reviewing our manuscript and for the insightful comments, which have helped us improve the clarity and quality of the work. **1. Although the article used multiple international datasets for pre-training and downstream task validation, the explanation for the improved performance of the "CAPE-Z" model (using the IDB strategy) on external data remains somewhat general. Further exploration is needed to determine whether the IDB strategy is more effective in certain subgroups to enhance the transparency and interpretability of the mechanism.**

We thank the reviewer for this insightful comment. We agree that understanding whether the benefits of the in-distribution batch (IDB) strategy are preferentially associated with specific subgroups is important for improving interpretability and mechanistic transparency. Although a comprehensive subgroup analysis across all demographic and clinical variables was beyond the scope of this study and is acknowledged as a limitation, we performed a limited subgroup analysis for the five-year mortality and reduced ejection fraction prediction task stratified by sex. We selected the endpoints being clinically meaningful with sex distribution relatively balanced across cohorts, whereas other variables such as age were more susceptible to imbalance and confounding effects.

Across datasets, we did not observe consistent evidence that the IDB strategy preferentially improved performance in one sex over the other. In BIDMC, both CAPE-X and CAPE-Z demonstrated nearly identical performance between males and females. In CODE, subgroup differences were statistically significant for CAPE-Z, whereas CAPE-X demonstrated more balanced performance across sexes, albeit with a substantially lower overall AUC. In UKB, larger sex-specific performance differences were observed; however, this pattern was present in both CAPE-X and CAPE-Z, suggesting that the effect was more likely attributable to dataset-specific characteristics, including low event incidence (~0.4% mortality), rather than the IDB strategy itself.

To improve transparency, we have added the detailed subgroup analysis as Supplementary Table S2 and clarified these findings in the manuscript.

**Supplementary information**

Table S2. **Sex-stratified performance comparison of foundation models pretrained using random batch and in-distribution batch (IDB) strategies for five-year mortality prediction.** The performance is reported as AUROC. AUC (SE) is reported for male and female subgroups, and ΔAUC quantifies the difference in performance between groups. Performance metrics are reported as mean values with standard error (SE) in parentheses, for example 0.805(002) indicates a mean of 0.805 with an SE of 0.002.

| No. | Dataset | Model | Male AUC | Female AUC | ΔAUC | p |
| --- | --- | --- | --- | --- | --- | --- |
| 1. | BIDMC (male =52%) | CAPE-X | 0.805 (002) | 0.803 (002) | 0.002 (003) | 0.496 |
| 2. |  | CAPE-Z | **0.823 (002)** | **0.823 (002)** | **0.000 (002)** | **0.734** |
| 3. | CODE  (male =43%) | CAPE-X | 0.695 (013) | 0.686 (015) | **0.009 (007)** | **0.496** |
| 4. |  | CAPE-Z | **0.806 (003)** | **0.796 (004)** | 0.011 (003) | 0.008 |
| 5. | UKB  (male =50%) | CAPE-X | **0.680 (005)** | 0.627 (008) | 0.052 (009) | 0.004 |
| 6. |  | CAPE-Z | 0.676 (003) | 0.632 (005) | **0.042 (006)** | **0.004** |

Table S3. **Sex-stratified performance comparison of foundation models pretrained using random batch and in-distribution batch (IDB) strategies for reduced ejection fraction < 40 classification.** AUC (SE) is reported for male and female subgroups, and ΔAUC quantifies the difference in performance between groups. Performance metrics are reported as mean values with standard error (SE) in parentheses, for example 0.805(002) indicates a mean of 0.805 with an SE of 0.002.

| No. | Dataset | Model | Male AUC | Female AUC | ΔAUC | p |
| --- | --- | --- | --- | --- | --- | --- |
| 1. | BIDMC (male =52%) | CAPE-X | **0.894 (002)** | 0.886 (004) | **0.006 (004)** | **0.203** |
| 2. |  | CAPE-Z | 0.884 (002) | **0.888 (002)** | **0.006 (004)** | **0.250** |
| 3. | SHZS  (male =55%) | CAPE-X | 0.567 (018) | 0.604 (013) | 0.037 (013) | 0.020 |
| 4. |  | CAPE-Z | **0.964 (002)** | **0.972 (003)** | **0.008 (004)** | **0.074** |
| 5. | UKB  (male =48%) | CAPE-X | 0.866 (005) | **0.827 (017)** | **0.039 (019)** | **0.074** |
| 6. |  | CAPE-Z | **0.877 (008)** | 0.726 (014) | 0.151 (015) | 0.004 |

**Manuscript Page 15, Line 325 (Results):**

*‘Sex-stratified analyses of five-year mortality likewise reveal no consistent performance differences between models across cohorts, with observed performance disparities primarily attributable to cohort-level variation rather than model architecture (Supplementary Table S2).’*

**Manuscript Page 20, Line 452 (Discussion):**

*‘We also explored sex-stratified performance for five-year mortality and did not observe consistent subgroup-specific differences between CAPE-X and CAPE-Z. This suggests that the improvements associated with the IDB strategy are not driven by demographic subgroup effects but are more likely related to improved robustness to cohort-level distribution shifts. The observed variability across cohorts appears to be influenced by factors such as outcome incidence and dataset-specific characteristics rather than model bias towards a particular subgroup.’*

Further representation analyses also suggest that learned features are influenced more strongly by cohort-level technical factors than by demographic attributes such as sex. As discussed further in Response 4, t-SNE analyses of ECG representations from different acquisition devices within the CODE dataset revealed clustering associated with device-specific frequency spectrum characteristics. After spectral alignment, this clustering was substantially reduced, supporting the hypothesis that technical signal characteristics contribute to representation learning and external generalisation.

Taken together, these findings suggest that the IDB strategy primarily improves robustness to cohort-level distribution shifts and reduces sensitivity to technical variations across datasets, rather than conferring advantages to specific demographic subgroups.

In this study, the different cohorts represent distinct populations, encompassing variation in ethnicity, socio-economic background, clinical settings, recording devices, and measurement protocols. We acknowledge that bias may still arise from the distribution of the downstream cohort used for supervised training. The age distributions of the different cohorts are shown in Fig. 6A, while Fig. 6B presents MAE across age groups. Error is lowest near the centre of the distribution and increases towards the tails, suggesting that cohorts with more samples concentrated around the mean age range of the training distribution achieve lower MAE. Improving the generalisation of downstream supervised models in the presence of biased labelled data remains an important direction for future research, and we have included this in the limitations.

The corresponding figures and text have been updated as follows:

**Manuscript Figure 6**

**Fig. 6 Impact of age distribution on prediction error for CAPE-Z embeddings with a prediction head trained on BIDMC and tested on external cohorts.** A) Age distributions across different datasets, illustrating mean age and range. B) Mean absolute error (MAE) normalized within each age bin, highlighting the age with minimum MAE and the overall age range. The distribution of labels significantly influences the aggregated performance metrics, as error varies across the age spectrum. Notably, the UK Biobank dataset exhibits a narrower age range with distinct peaks corresponding to ages associated with the lowest MAE.

**Manuscript Page 22, Line 489 (Limitations)**

*‘Our current work explores pretraining cohorts that differ across multiple complex factors, including ethnicity, socio-economic status, health status, hospital systems, and recording devices. We suggest that the individual contribution of each factor should be systematically investigated in future studies. Our analysis is limited to pretraining strategies, while performance remains influenced by bias in downstream datasets, which could be addressed through more detailed subgroup analyses and improved optimisation of downstream supervised training.’*

**2. The article repeatedly mentions "healthy populations," but the definition criteria for these groups are not clearly defined. Especially in the comparison between UKB and CODE, differences in health status may affect model performance. It is recommended to supplement the health screening criteria or relevant clinical indicators for these populations.**

We thank the reviewers for this important comment. We have clarified the definition of “healthy populations” in the revised manuscript. Specifically, we now define cohort health status using both cohort origin (primary care versus secondary care cohorts) and a quantitative clinical indicator, 5-year mortality, as a proxy for disease burden. We have added these values in Tables 1 and 2 for the cohorts where the mortality information was available and updated the manuscript text to explicitly describe these criteria and their relevance to model performance comparisons.

The five-year mortality for the secondary care cohorts such as BIDMC and VUMC have substantially higher values (35% and 32%, respectively) compared to primary care population-based cohorts such as CODE (9–16%) and volunteer cohort of UKB (0.4%).

We hypothesise that cohorts with a higher disease burden exhibit greater physiological diversity, which may contribute to improved robustness of pretrained models. In contrast, when validating supervised models, secondary care cohorts show lower performance compared with primary care cohorts. The healthiest cohort, UK Biobank (with the lowest 5-year mortality rate), shows the lowest age prediction MAE and highest sex prediction AUC. An additional contributing factor may be that the age range of UKB participants is narrower and closer to the mean age of the Beth Israel Deaconess Medical Center cohort, on which the supervised model was trained. The manuscript has been updated accordingly.

**Table 1. Baseline characteristics of the pretraining cohorts included in this study (p < 0.0001 for all pairwise comparisons of age, sex, and mortality).**

|  | BIDMC^∗^ | CODE^∗^ | SHZS^∗^ | VUMC^∗^ |
| --- | --- | --- | --- | --- |
| Location | US | Brazil | China | US |
| Care level | secondary | primary | Primary | Secondary |
| Patients^∗^ | 127,041 | 424,577 | 420,956 | 252,294 |
| ECGs | 1,106,886 | 1,123,903 | 1,560,551 | 1,411,929 |
| Age mean | 64.46 | 56.57 | 54.15 | 60.21 |
| Age IQR | 21.09 | 24.00 | 26.00 | 22.24 |
| Male | 571,944 (52%) | 437,188 (39%) | 874,969 (56%) | 751,040 (54%) |
| Mortality (5y)** | 236,294 (35%) | 5,125 (16%) | _ | 89,900 (32%) |
| Hispanic | 7,077 | − | − | − |
| White | 84,265 | − | − | − |
| Black | 17,778 | − | − | − |
| Asian | 5,315 | − | − | − |
| Other | 12,606 | − | − | − |

∗ Pretraining cohort with patients having more than one ECG

** Includes only ECGs with five-year follow-up

**Table 2. Baseline characteristics of the cohorts explored for the downstream supervised tasks (BIDMC, CODE, SHZS, UKB, and PTB-XL). p < 0.0001 for all pairwise comparisons of age, sex, and mortality.**

|  | BIDMC | CODE | SHZS | VUMC | UKB | PTB-XL |
| --- | --- | --- | --- | --- | --- | --- |
| Location | US | Brazil | China | US | UK | Germany |
| Care level | secondary | primary | primary | secondary | primary | Secondary |
| Patients^∗^ | 189,542 | 1,503,229 | 1,117,197 | 526,017 | 66,402 | 18,869 |
| ECGs | 1,169,387 | 2,202,555 | 2,257,485 | 1,685,652 | 70,655 | 21, 799 |
| Age mean | 63.73 | 53.54 | 53.62 | 58.76 | 65.31 | 62.77 |
| Age IQR | 21.67 | 25.00 | 26.00 | 23.73 | 12.00 | 22.00 |
| Male | 599,731 (51%) | 878,668 (40%) | 1,235,353 (55%) | 876,790 (53%) | 34,265 (49%) | 11,354 (52%) |
| Mortality (5y)* | 242,085 (35%) | 12,575 (9%) | - | 104,130 (26%) | 68 (0.4%) | - |
| Hispanic | 10,248 | − | − |  | − | − |
| White | 123,063 | − | − |  | − | − |
| Black | 24,251 | − | − |  | − | − |
| Asian | 8,924 | − | − |  | − | − |
| Other | 23,056 | − | − |  | − | − |

* Includes only ECGs with five-year follow-up.

**Manuscript Page 9, Line 182:**

*‘The 5-year mortality (among patients with 5-year follow-up) for the secondary care cohorts, BIDMC and VUMC, is 35% and 32%, respectively, compared with15% for the CODE cohort.’*

**Manuscript Page 9, Line 190:**

*‘The 5-year mortality among patients with five-year follow-up in the labelled cohort is 35% for BIDMC, 26% for VUMC, 9% for CODE, and 0.4% for the UKB. ‘*

**3. To ensure fairness in the comparison, performance evaluation metrics are derived from only a subset of 10,000 training samples and 2,000 test samples randomly selected from each cohort. Therefore, the reported performance results do not reflect the model's true performance on the full cohort dataset.**

Thank you for pointing this out and for the opportunity to further clarify this aspect of the evaluation. We agree that the reported performance metrics are derived from only a subset of samples from each cohort, and that this distinction is important for correct interpretation of the results.

The use of a fixed number of samples (10,000 for training and 2,000 for testing) from each dataset during supervised training was a deliberate design choice to ensure a fair and controlled comparison across cohorts with substantially different dataset sizes. This approach minimises confounding effects arising from dataset scale and prevents larger cohorts from disproportionately influencing the evaluation outcomes.

We also agree that evaluating on the full cohort datasets would be valuable for obtaining a more comprehensive estimate of absolute model performance and state-of-the-art benchmarking. However, the objective of this study is different. Rather than maximising cohort-specific performance, our aim is to study model generalisation behaviour under a standardised and comparable sampling framework across all datasets. This setup therefore allows differences in performance to be interpreted more directly in terms of cohort characteristics, rather than differences in dataset size or composition.

The following text is included in the manuscript:

**Manuscript Page 22, Line 496 (Limitations):**

*‘This study is not intended to provide state-of-the-art label prediction performance across cohorts, but rather to evaluate how supervised training behaves when using features from different pretraining methodologies while keeping labelled dataset sizes comparable. We therefore use random splits of equal size from each cohort for training and validation, based on features extracted from a frozen backbone, to ensure fair comparisons. Training the prediction head on the full cohort followed by end-to-end fine-tuning was beyond the scope of the present study and not directly aligned with our primary research objective. Future work may establish state-of-the-art performance for each cohort and further evaluate the impact of fine-tuning on model generalisation and predictive performance.’*

**4. The t-SNE plot shows a significant clustering phenomenon in the features of the CAPE-X model, which the authors speculate is related to device type. However, quantitative analysis is lacking to support this conclusion.**

Thank you for this important suggestion. We agree that qualitative inspection of t-SNE visualisations alone is insufficient to support conclusions regarding clustering structure. To address this concern, we have added a quantitative analysis using Maximum Mean Discrepancy (MMD), computed on the same low-dimensional representation used for visualisation (the first two principal components derived from the learned embeddings). MMD provides a non-parametric measure of distributional difference and enables quantitative comparison of group separation within a shared representation space.

We acknowledge that the learned embeddings are high-dimensional and may encode multiple interacting factors in a complex manner. However, the first two principal components capture the dominant directions of variance in the representation space and therefore provide a meaningful low-dimensional summary for comparative analysis. Within this space, we observe clear and consistent separation between device types in the CODE cohort, whereas substantially weaker separation is observed across ethnicity groups in the BIDMC cohort.

Quantitatively, device-based grouping in CODE demonstrates strong separation (MMD = 0.873), while ethnicity-based grouping in BIDMC shows minimal separation (MMD = 0.007). These findings are consistent with the visual clustering patterns observed in the t-SNE plots and support the conclusion that device-related factors contribute more strongly than patient demographics to the dominant structure observed in the learned representation space.

**Manuscript Figure 4**

Fig 4. t-SNE visualization of learned embeddings from the CAPE-X model form clusters across cohorts. The CAPE-X model was pretrained on the combined BCSV dataset using random batches. (A) The learned feature representation, projected onto the first two components, shows distinct clustering by cohort, with overlap observed between BIDMC and PTB-XL. (B) Within the BIDMC cohort, features from different ethnic groups exhibit substantial overlap, with low distributional separation in the component space (MMD = 0.007). (C) In contrast, features from the CODE dataset form two clearly separated clusters corresponding to recording device types, indicating strong distributional shift in the component space (MMD = 0.873).

**Manuscript Page 13, Line 279 (Results):**

*‘We quantify distributional differences between groups using Maximum Mean Discrepancy (MMD), a non-parametric metric that measures the distance between two probability distributions in a reproducing kernel Hilbert space. Higher MMD values indicate greater separation between group feature distributions, while lower values suggest substantial overlap. Visualisations of the first two t-SNE components show that within BIDMC, ethnicity distributions exhibit minimal separation (MMD = 0.007), while within CODE, device types show strong separation (MMD = 0.873). This suggests that technical factors may contribute more strongly than patient demographics to the dominant structure observed in the t-SNE representation.’*

**5. While the IDB strategy performs well in practice, the article does not provide a theoretical analysis of why it improves generalization ability. For example, is it related to reducing intra-batch distribution confusion and decreasing the model's dependence on spurious correlation features?**

We thank the reviewer for this insightful comment. We agree that a full theoretical characterisation of the IDB strategy is beyond the scope of this work; however, the reviewer’s interpretation aligns well with our understanding, and we provide a conceptual and empirical explanation for the observed improvements in generalisation.

In standard contrastive learning, the model maximises similarity between positive pairs while distinguishing them from negatives within a batch, implicitly assuming that negatives are drawn from a comparable distribution. When batches are formed from randomly sampled ECGs across different cohorts, this assumption is violated, as multiple distributions (e.g. due to differences in devices, populations, or preprocessing) coexist within a batch. This can lead to intra-batch distribution confusion, where the model exploits cohort-related distributional differences rather than meaningful physiological variation. In addition, such mixing increases reliance on spurious correlation features, such as cohort- or device-specific artefacts, which can act as shortcuts for discrimination but do not generalise.

The IDB strategy mitigates these issues by restricting each batch to a single cohort. This reduces intra-batch distribution confusion by ensuring a more consistent data distribution within each batch, and it discourages reliance on spurious correlations by removing cohort identity as an easy discriminative cue. Across training, exposure to multiple cohorts is still maintained, encouraging learning of representations that are robust to inter-cohort variation.

Our empirical results support this interpretation, showing improved external cohort performance and reduced cohort-driven clustering in the representation space. We have clarified this motivation in the Discussion section of the revised manuscript.

**Manuscript Page 20, Line 433 (Discussion):**

*‘The CAPE model, when trained with batches formed from multiple cohorts, may inadvertently learn cohort-specific signals such as device characteristics, acquisition settings, or preprocessing artefacts. Although not clinically meaningful, these can be exploited during contrastive learning, leading to* intra-batch distribution confusion *where multiple underlying distributions coexist within a batch and the model relies on spurious correlation features rather than robust physiological patterns, potentially harming generalisation to unseen cohorts. In contrast, CAPE-Z enforces cohort-level consistency within each batch, reducing distributional heterogeneity and limiting the use of cohort-specific artefacts as discriminative shortcuts, thereby promoting more stable and clinically relevant representations. Empirically, this results in improved out-of-distribution performance, with the largest gains in pretraining cohorts, supporting the hypothesis that standard contrastive pretraining can capture cohort-specific confounding signals, alongside smaller but consistent improvements in external cohorts, indicating enhanced generalisation.’*

We also add a flow chart of the IDB approach to further clarify as following:

**Manuscript Figure 8:**

Fig 8. Flowchart of the in-distribution batch (IDB) technique: for each epoch, batches are constructed from the same cohort using two randomly selected ECGs per patient.

**6. The article mentions using the Kruskal-Wallis test and the Wilcoxon test for comparison, but does not explain the correction method for multiple comparisons (e.g., Bonferroni correction). Without correction in multiple comparisons, the Type I error rate may increase. It is recommended to supplement the explanation of whether multiple comparison correction was performed.**

We thank the reviewers for the suggestion regarding multiple-comparison control in our statistical analysis. In our experiments, prediction heads were trained on feature sets from different cohorts and evaluated across datasets. We first used the Kruskal–Wallis test to assess overall significance, and we have now added post hoc pairwise comparisons with Holm–Bonferroni correction to account for multiple comparisons.

The Wilcoxon signed-rank and DeLong tests were applied only in single pairwise comparison settings per evaluation; therefore, no multiple-comparison correction was required. For Wilcoxon, p-values were computed from AUC distributions across multiple runs, while for DeLong they were derived from aggregated model predictions across runs. We have clarified this in the manuscript as follows:

**Manuscript Page 11, Line 230 (Results):**

*‘Models pretrained on the multi-centre BCSV dataset (CAPE-X) achieve the best overall performance, demonstrating the benefit of pretraining on a large and diverse dataset. CAPE-X achieved the lowest mean MAE for age prediction (7.86, 95% CI: 7.70–7.82), with overall differences across cohorts being significant (Kruskal–Wallis, p = 0.0002). Post hoc Holm–Bonferroni-corrected comparisons indicated that BIDMC, SHZS, and VUMC did not differ from each other, whereas all other pairwise differences were significant (padj = 0.0395). For sex prediction, CAPE-X achieved the highest mean AUROC (0.955, 95% CI: 0.955–0.956), with overall cohort differences significant (Kruskal–Wallis, p<0.0001); post hoc comparisons showed that BIDMC and VUMC were not significantly different, while all other pairwise differences were significant (padj = 0.0395). Among CAPE models pretrained on single cohorts, those trained on secondary care datasets such as BIDMC (age MAE: 8.19, 95% CI8.10–8.28; sex AUROC: 0.942, 95% CI: 0.941–0.942) and VUMC (age MAE: 8.20, 95% CI: 8.12–08.27; sex AUROC: 0.942, 95% CI: 0.942–0.943) outperform those trained on healthier population-based cohorts such as CODE (age MAE: 8.40, 95% CI: 8.35–8.45; sex AUROC: 0.934, 95% CI: 0.934–0.935) and SHZS (age MAE:8.23, 95% CI: 8.16–8.30; sex AUROC: 0.924, 95% CI: 0.924–0.925, with differences significant at padj < 0.05.’*

**Manuscript Page 28, Line 614 (Methods):**

*‘To compare CAPE models pretrained on different datasets, statistical significance is assessed using the Kruskal–Wallis test, with pairwise comparisons corrected using the Holm–Bonferroni method. Comparisons between the In-Distribution Batch (IDB) strategy and training with randomly sampled batches is performed using the Wilcoxon signed-rank test for age prediction (measured by MAE) and the DeLong’s test applied to model prediction scores for sex classification, with each comparison evaluated as a single hypothesis tes.t ‘*

Detailed pair-wise statistics are also added to the supplementary material as follows:

**Mean MAE of all Age labels:**

Kruskal-Wallis: H=22.482, p=0.0002

CAPE-B vs CAPE-C: raw p=0.0039, corrected p=0.0395

CAPE-B vs CAPE-S: raw p=0.4233, corrected p=1.0000

CAPE-B vs CAPE-V: raw p=0.6310, corrected p=1.0000

CAPE-B vs CAPE-X: raw p=0.0039, corrected p=0.0395

CAPE-C vs CAPE-S: raw p=0.0065, corrected p=0.0395

CAPE-C vs CAPE-V: raw p=0.0039, corrected p=0.0395

CAPE-C vs CAPE-X: raw p=0.0039, corrected p=0.0395

CAPE-S vs CAPE-V: raw p=0.3367, corrected p=1.0000

CAPE-S vs CAPE-X: raw p=0.0039, corrected p=0.0395

CAPE-V vs CAPE-X: raw p=0.0039, corrected p=0.0395

**Mean AUC Sex:**

Kruskal-Wallis: H=26.826, p=0.0000

CAPE-B vs CAPE-C: raw p=0.0039, corrected p=0.0395

CAPE-B vs CAPE-S: raw p=0.0039, corrected p=0.0395

CAPE-B vs CAPE-V: raw p=0.1495, corrected p=0.1495

CAPE-B vs CAPE-X: raw p=0.0039, corrected p=0.0395

CAPE-C vs CAPE-S: raw p=0.0039, corrected p=0.0395

CAPE-C vs CAPE-V: raw p=0.0039, corrected p=0.0395

CAPE-C vs CAPE-X: raw p=0.0039, corrected p=0.0395

CAPE-S vs CAPE-V: raw p=0.0039, corrected p=0.0395

CAPE-S vs CAPE-X: raw p=0.0039, corrected p=0.0395

CAPE-V vs CAPE-X: raw p=0.0039, corrected p=0.0395

**7. Although the authors provide small-scale sample code, overall reproducibility is limited because the core model and proprietary data cannot be publicly disclosed. It is recommended to disclose the model weights or provide an API interface, to the extent possible, to facilitate other researchers' validation and expansion of the research.**

We agree with the reviewer that open-source resources would further facilitate validation and extension of this work. However, as the model was trained using private datasets, we are currently unable to publicly release the full model weights without explicit permission from all contributing parties, which is not available at this stage.

To improve reproducibility within these constraints, we have provided sample code and released features derived from two versions of our model on the open datasets PTB-XL and CODE15 (a subset of CODE). These features demonstrate the performance of our IDB approach and can support further analysis on these cohorts. We have also ensured that the methodological details are described sufficiently for replication, and we have added a new Figure 8 to clarify the IDB approach in greater detail.

**Manuscript Figure 8:**

Fig 8. Flowchart of the in-distribution batch (IDB) technique: for each epoch, batches are constructed from the same cohort using two randomly selected ECGs per patient.

*.* **8. The figures in the article are of very poor quality. All figures are poorly readable. Many images in the paper are simply screenshots, and some even contain spelling errors.**

We thank the reviewer for this comment. We have addressed the issue of figure quality and clarity in the revised manuscript. Figure 4 has been reorganised and split into two figures to improve interpretability, separating t-SNE analyses of random batches versus cohorts (A), ethnicity (B), and recording device (C), and the comparison of CAPE models trained on single cohorts versus the multi-cohort IDB-trained model (now presented as Figure 5). In addition, a new figure has been added showing a flowchart of the IDB approach to better illustrate the methodology. All figures have been regenerated in high-resolution TIFF format, and spelling and labelling errors have been corrected to improve readability and presentation quality.

**Manuscript Figure 4**

 **Fig. 4 t-SNE visualization of learned embeddings from the CAPE-X model form clusters across cohorts.** The CAPE-X model was pretrained on the combined BCSV dataset using random batches. (A) The learned feature representation, projected onto the first two components, shows distinct clustering by cohort, with overlap observed between BIDMC and PTB-XL. (B) Within the BIDMC cohort, features from different ethnic groups exhibit substantial overlap, with low distributional separation in the component space (MMD = 0.007). (C) In contrast, features from the CODE dataset form two clearly separated clusters corresponding to recording device types, indicating strong distributional shift in the component space (MMD = 0.873).

Fig 5. t-SNE visualization of learned embeddings. Feature embeddings overlap across cohorts without forming distinct clusters. A) CAPE-B, pretrained on BIDMC alone, shows no clear clustering, although a weak cohort-associated gradient is apparent. B) CAPE-Z, pretrained on the combined BCSV dataset using the in-distribution batch (IDB) strategy, likewise shows no apparent cohort-specific clustering.

 Fig 6. Impact of age distribution on prediction error for CAPE-Z embeddings with a prediction head trained on BIDMC and tested on external cohorts. A) Age distributions across different datasets, illustrating mean age and range. B) Mean absolute error (MAE) normalized within each age bin, highlighting the age with minimum MAE and the overall age range. The distribution of labels significantly influences the aggregated performance metrics, as error varies across the age spectrum. Notably, the UK Biobank dataset exhibits a narrower age range with distinct peaks corresponding to ages associated with the lowest MAE.

**Manuscript Figure 7:**

Fig 7. Overview of the CAPE pretraining framework. Patient A has two ECG recordings (ECG1A and ECG2A), and Patient B, another patient in the same batch, has ECG1B and ECG2B. Each ECG undergoes temporal augmentations before being encoded by the network into a 256-dimensional feature vector. The contrastive loss encourages embeddings from the same patient’s ECGs to be closer together while pushing apart embeddings from different patients within the batch.

**Manuscript Figure 8:**

**Fig. 8 Flowchart of the in-distribution batch (IDB) technique**: for each epoch, batches are constructed from the same cohort using two randomly selected ECGs per patient.

**9. The article title and abstract mention "clinically fair," but the text does not systematically evaluate the model's performance differences across different genders, races, or socioeconomic groups. It is recommended to supplement this with quantitative analysis of fairness, such as group AUC or MAE difference tests.**

We thank the reviewer for this important comment regarding fairness evaluation. We agree that the term “clinically fair” implies a systematic subgroup analysis, and we have therefore revised the abstract to avoid potential misinterpretation.

In this work, our primary focus is on how pretraining cohort composition and the proposed IDB strategy affect representation learning and generalisation, rather than on a dedicated fairness optimisation framework. Nevertheless, we have added additional analyses to improve transparency.

Specifically, we report sex-stratified results for the five-year mortality and reduced ejection fraction prediction task in the Supplementary Material. Across datasets, we do not observe consistent or systematic performance differences between males and females, and any observed variations are small and not consistently directional.

We also include an analysis of MAE across age groups under the IDB setting, showing that performance varies with age rather than remaining uniform, highlighting the influence of label distribution and cohort composition on aggregate metrics.

We acknowledge that a full evaluation across race or socioeconomic subgroups is not included due to the limited availability of such annotations in the datasets used. We have clarified this limitation in the revised manuscript and emphasize that more comprehensive subgroup analyses, such as group-wise AUC or MAE comparisons, are an important direction for future work.

**Supplementary Information Table S2:**

Table S2. **Sex-stratified performance comparison of foundation models pretrained using random batch and in-distribution batch (IDB) strategies for five-year mortality prediction.** AUC (SE) is reported for male and female subgroups, and ΔAUC quantifies the difference in performance between groups. Performance metrics are reported as mean values with standard error (SE) in parentheses, for example 0.805(002) indicates a mean of 0.805 with an SE of 0.002.

| No. | Dataset | Model | Male AUC | Female AUC | ΔAUC | p |
| --- | --- | --- | --- | --- | --- | --- |
| 1. | BIDMC (male =52%) | CAPE-X | 0.805 (002) | 0.803 (002) | 0.002 (003) | 0.496 |
| 2. |  | CAPE-Z | **0.823 (002)** | **0.823 (002)** | **0.000 (002)** | **0.734** |
| 3. | CODE  (male =43%) | CAPE-X | 0.695 (013) | 0.686 (015) | **0.009 (007)** | **0.496** |
| 4. |  | CAPE-Z | **0.806 (003)** | **0.796 (004)** | 0.011 (003) | 0.008 |
| 5. | UKB  (male =50%) | CAPE-X | **0.680 (005)** | 0.627 (008) | 0.052 (009) | 0.004 |
| 6. |  | CAPE-Z | 0.676 (003) | 0.632 (005) | **0.042 (006)** | **0.004** |

Table S3 | **Sex-stratified performance comparison of foundation models pretrained using random batch and in-distribution batch (IDB) strategies for reduced ejection fraction < 40 classification.** AUC (SE) is reported for male and female subgroups, and ΔAUC quantifies the difference in performance between groups. Performance metrics are reported as mean values with standard error (SE) in parentheses, for example 0.805(002) indicates a mean of 0.805 with an SE of 0.002.

| No. | Dataset | Model | Male AUC | Female AUC | ΔAUC | p |
| --- | --- | --- | --- | --- | --- | --- |
| 1. | BIDMC (male =52%) | CAPE-X | **0.892 (002)** | 0.886 (004) | **0.006 (005)** | **0.203** |
| 2. |  | CAPE-Z | 0.884 (002) | **0.888 (002)** | **0.006 (004)** | **0.250** |
| 3. | SHZS  (male =55%) | CAPE-X | 0.567 (018) | 0.604 (013) | 0.037 (013) | 0.020 |
| 4. |  | CAPE-Z | **0.964 (002)** | **0.972 (003)** | **0.008 (004)** | **0.074** |
| 5. | UKB  (male =48%) | CAPE-X | 0.866 (005) | **0.827 (008)** | **0.038 (019)** | **0.074** |
| 6. |  | CAPE-Z | **0.877 (008)** | 0.726 (014) | 0.154 (015) | 0.004 |

The following changes were made in the manuscript:

**Manuscript Page 4, Line 81 (Abstract):**

*‘This work provides insights into pretraining strategies for more clinically deployable and generalisable foundation models.’*

**Manuscript Page 15, Line 322 (Results):**

‘*Sex-stratified analyses of five-year mortality likewise reveal no consistent performance differences between models across cohorts, with observed performance disparities primarily attributable to cohort-level variation rather than model architecture (Supplementary Table S2).*’

**Manuscript Page 20, Line 448 (Discussion):**

*‘We also explored sex-stratified performance for five-year mortality and did not observe consistent subgroup-specific differences between CAPE-X and CAPE-Z. This suggests that the improvements associated with the IDB strategy are not driven by demographic subgroup effects but are more likely related to improved robustness to cohort-level distribution shifts. The observed variability across cohorts appears to be influenced by factors such as outcome prevalence and dataset-specific characteristics rather than model bias towards a particular subgroup.’*

**Reviewer 2:**

**1. Some grammatical errors (e.g., "Deep learning models, often struggle with generalisation").**

Thank you for pointing out this error. We have corrected the mentioned sentence (“Deep learning models often struggle with generalisation”) and also performed a careful proofreading of the manuscript to fix additional grammatical issues.

**2. Some claims would be strengthened by citations (e.g., "This challenge is further compounded by significant heterogeneity across ECG cohorts, stemming from differences in age distribution, clinical profiles, recording devices, and institutional practices.").**

We thank the reviewer for this helpful suggestion. We agree that supporting this statement with citations strengthens the claim. Accordingly, we have revised the manuscript as follows:

**Manuscript Page 6, Line 125 (Introduction):**

*‘This challenge is further compounded by significant heterogeneity across cohorts, stemming from differences in age distribution, clinical profiles, recording devices, and institutional practices^17, 20^.’*

We have added the following references to support this statement:

17. Soltanieh S, Hashemi J, Etemad A. In-distribution and out-of-distribution self-supervised ECG representation learning for arrhythmia detection. IEEE J Biomed Health Inform. 2024;28(2):789–800.

20.	Strodthoff N, Wagner P, Schaeffter T, Samek W. Deep learning for ECG analysis: benchmarks and insights from PTB-XL. IEEE J Biomed Health Inform. 2021;25:1519–1528.

**3. "a retinal image–based foundational model trained on British patient data demonstrated poor transferability to Chinese cohorts, performing comparably to model pretrained on natural images."
- I might clarify this with, "Explorations performed on other medical data modalities...", otherwise it's jarring.**

We thank the reviewer for this helpful suggestion. We agree that the original phrasing was abrupt, and we have revised the sentence to improve readability and flow by adding an introductory framing. The corrected text now reads:

**Manuscript Page 7, Line 131 (Introduction):**

*‘Foundation models must overcome domain shift to be clinically viable, a challenge observed across multiple medical modalities. For example, a retinal image-based foundation model trained on British patient data demonstrated poor transferability to Chinese cohorts^24^.’*

**4. "Initially, CAPE models are pretrained to learn generic ECG features, which are then generated for each labeled cohort. This approach significantly reduces training time for downstream tasks by leveraging these precomputed features."
- This is essentially a linear probing strategy, which is often worse performance than full fine-tuning, and therefore more impressive if done well. You might consider addressing this? Especially if/since you're not receiving SOTA results, which I don't believe is your objective here.
- I do see that you address this in your limitations, but again, you might benefit from better framing here.**

We thank the reviewer for this insightful comment. We agree that strong performance under a frozen-feature, linear-probe-style evaluation is particularly meaningful, as it indicates that the pretrained backbone has learned robust and transferable representations without relying on extensive downstream optimisation. Our objective was therefore not to achieve state-of-the-art performance through full end-to-end fine-tuning, but rather to investigate how pretraining cohort composition and the proposed IDB strategy influence representation quality and generalisability under a controlled and consistent evaluation framework.

We have clarified this framing in the manuscript to better reflect the intended focus of the study and to avoid potential confusion regarding the optimisation setting. In addition, we explored downstream fine-tuning in Supplementary Table S5 and observed trends consistent with the linear-probe results. In particular, fine-tuning did not recover the reduced external validation performance observed for CAPE-X, whereas CAPE-Z continued to demonstrate robust generalisation across external cohorts. These findings suggest that the observed differences primarily arise from the robustness of the learned representations rather than limitations of the downstream optimisation strategy.

**Manuscript Page 9, Line 197 (Results):**

*‘Supervised learning is performed in a manner similar to a linear probe, except that a non-linear multi-layer perceptron (MLP) prediction head is used. Linear probing is a standard methodology to compare different pretraining approaches and assess the quality of learned representations without any bias introduced by downstream optimisation (e.g., SimCLR^12)^. Moreover, the training time is substantially reduced by leveraging precomputed features, which is essential given the scale of the experiments.’*

**5. While I understand the constraints you're working with due to different label composition, age and sex tasks are hardly clinically salient. At the very least, I feel this should have been addressed more clearly/honestly.
- Coming back to this comment, I do now see that you've also done reduced LVEF and five-year mortality. If appropriate, I may have highlighted this earlier on, and also made this discussion bigger, because it lends clinical significance to your work!**

We thank the reviewer for this important suggestion. Age and sex prediction are not, in themselves, clinically meaningful endpoints. In our study, however, we used these tasks as “canary in a coal mine” proxies because they are consistently available across all cohorts, enabling systematic and large-scale comparisons aligned with our research objectives. We would like to clarify that clinically meaningful labels, specifically low ejection fraction and five-year mortality are included in our downstream evaluations, but this was not clearly stated or sufficiently emphasized in the original manuscript. We regret this lack of clarity.

In the revised manuscript, we now explicitly highlight these clinical tasks from the outset and clarify their role in validating our findings. We note that the same trends identified using age and sex labels are also reflected in these clinically meaningful endpoints, strengthening our conclusions. As these labels are available only for a subset of cohorts, they are used to corroborate our findings rather than serve as the basis for uniform comparisons across all datasets. We have revised the section describing the training configurations and experimental setup to clearly articulate this distinction and better motivate our evaluation strategy.

**Manuscript Page 10, Line 206 (Results):**

*‘Supervised tasks implemented in the current work include predicting age and sex, as well as reduced left ventricular ejection fraction and five-year mortality. Demographic labels such as age and sex are available across all cohorts and have been shown to be implicitly encoded in ECG signals^4, 20^, enabling broader comparison while remaining sufficiently challenging and representative. In contrast, clinical labels are heterogeneous across datasets and not uniformly available; thus, we use these tasks to further corroborate the primary findings from age and sex.’*

**6. AUROC is not always a robust metric when dealing with different label prevalence. I can't say whether you should include this metric in the paper, but I would make sure that Area Under the Precision-Recall-Gain curve (AUPRG) agrees with your AUROC-based conclusions (I've found this useful in my own work, at least).**

We thank the reviewer for this valuable suggestion. We agree that AUROC can be sensitive to differences in label prevalence, which is consistent with our observations that the same model can yield different AUROC values across cohorts. As such, we emphasise in the manuscript that performance across cohorts is not directly comparable unless evaluated on the same dataset.

In our study, methodological comparisons are always performed on the same data, ensuring a one-to-one pairing. For external validation, we further control this by evaluating all models on identical randomly sampled test sets using fixed seeds.

Following the reviewer’s suggestion, we additionally evaluated the Area Under the Precision–Recall Gain curve (AUPRG) for our classification tasks. The results, now included in Supplementary Table S1, are consistent with our AUROC-based findings, with CAPE-Z maintaining superior performance across tasks and cohorts. We believe this additional analysis strengthens the robustness of our conclusions.

**Manuscript Page 15, Line 323:**

*‘In addition, the area under the precision–recall gain curve (AUPRG), a metric particularly suited to imbalanced data, shows consistent performance trends (Supplementary Table S1).’*

**Supplementary Information:**

Table S1. Performance comparison of foundation models pretrained using random-batch and in-distribution batch (IDB) strategies, evaluated using the area under the Precision–Recall Gain curve (AUCPRG). The prediction head is trained on BIDMC labels across ten independent runs, with evaluation conducted on 10,000 random test samples from external cohorts. Performance metrics are reported as the mean with standard error (SE) in parentheses: e.g. the area under the Precision–Recall Gain curve (AUCPRG) of mean 0.859 and standard error 0.0019 as 0.859(019).

| No. | Model | BIDMC | CODE | SHZS | UKB | PTB-XL | mean |
| --- | --- | --- | --- | --- | --- | --- | --- |
| Sex prediction (AUPRG) | | | | | | | |
| 1 | CAPE-X | **0.859(019)** | -1.63(517) | 0.497(668) | 0.939(022) | 0.854(016) | 0.669 |
| 2 | CAPE-Z | 0.851(023) | **0.792(037)** | **0.939(016)** | **0.946(013)** | **0.875(013)** | **0.879** |
| 3 | p-value^$^ | <0.0001 | <0.0001 | 0.0002 | 0.0291 | <0.0001 | **-** |
| LVEF < 40 ( AUPRG ) | | | | | | | |
| 4 | CAPE-X | 0.849(025) | - | 0.289(334) | **0.850(106)** | - | 0.663 |
| 5 | CAPE-Z | **0.851(025)** | - | **0.959(015)** | 0.844(014) | - | **0.885** |
| 6 | p-value^$^ | 0.0255 | - | <0.0001 | 0.3183 | - | - |
| 5 year mortality ( AUPRG ) | | | | | | | |
| 7 | CAPE-X | 0.572(072) | 0.412(331) | - | **0.437(132)** | - | 0.483 |
| 8 | CAPE-Z | **0.617(033)** | **0.642(068)** | - | 0.424(058) | - | **0.547** |
| 9 | p-value^$^ | <0.0001 | 0.0001 | - | 0.2939 | - | - |

**7. "This design discourages the model from learning spurious cohort-distinguishing features and instead promotes the identification of clinically meaningful variability within each cohort."
- I might have put a line like this into your abstract. It's an intriguing design! Though I was curious if any prior works used anything like it in other contexts.**

Thank you for this helpful suggestion. We agree that this phrasing clearly highlights the intuition behind our design. We have incorporated a similar sentence into the abstract to better emphasise the role of our approach in discouraging spurious cohort-specific features and promoting clinically meaningful variability within cohorts.

**Manuscript Page 4, Line 77 (Abstract):**

‘*To address this, we propose the In-Distribution Batch (IDB) strategy, which preserves intra-cohort consistency during pretraining, discourages learning of spurious cohort-specific features, and instead promotes clinically meaningful variability within cohorts. This leads to improved out-of-distribution robustness, with gains of 9 to 40% in downstream label prediction performance.’*

Regarding your question about related work, prior approaches in invariant representation learning and domain generalisation, similarly aim to mitigate spurious domain- or cohort-specific signals and improve out-of-distribution generalisation. However, these methods typically enforce invariance through objective functions across environments. In contrast, our proposed In-Distribution Batch (IDB) strategy operates at the data sampling level, preserving intra-cohort consistency during pretraining to encourage meaningful variability within cohorts. To the best of our knowledge, this batch-based mechanism has not been widely explored in this form, and we have clarified this distinction in the revised manuscript.

We have added references and discussion of related work in domain generalisation to better position our contribution.

**Manuscript Page 7, Line 140 (Introduction):**

*‘Several approaches have been proposed to improve domain generalisation²⁵ across diverse domains. Uncertainty-aware and ensemble-based foundation models²⁶ improve external validation performance in glioblastoma classification by leveraging multiple models and uncertainty estimation to better distinguish tumours from their mimics. Contrastive learning with fairness objectives²⁷ enhances fairness in pathology AI systems by incorporating fairness constraints into the learning objective. For ECG tasks, self-supervised contrastive learning¹⁷ and aggregation of features from multiple intermediate layers²⁸ have been crucial for improving generalisation, collectively highlighting the need for models that generalise across diverse clinical environments.’*

**8. "CAPE models pretrained on secondary care cohorts outperform those pretrained on larger but healthier populations. A plausible explanation for this is thas1t healthier populations tend to exhibit reduced variability, whereas secondary care cohorts encompass a diverse range of cardiac signal patterns."
- You could likely strengthen this claim with some basic statistical analysis of the label distributions**

We thank the reviewer for this important comment regarding the definition of “healthy populations.” In the original manuscript, cohort origin was used as a proxy for health status, with primary care or volunteer cohorts considered relatively healthier than secondary care cohorts.

In response to the reviewer’s suggestion, we have now incorporated cohort-level 5-year mortality as a quantitative measure of health status, since other morphology- or rhythm-related information was unavailable for most cohorts, whereas mortality data were available for four cohorts. These values are now reported in Tables 1 and 2 for both the pretraining and downstream cohorts.

The updated results support our original interpretation, with secondary care cohorts such as BIDMC and VUMC showing substantially higher 5-year mortality (35% and 32%, respectively) compared with population-based cohorts such as CODE (9–16%) and UKB (0.4%). This suggests that cohorts with greater disease burden may contain broader physiological and ECG variability, which could contribute to improved robustness of pretrained representations. For all statistical tests, cohort characteristics differed significantly across all pairwise comparisons (p < 0.0001).

The corresponding discussion has been added to the revised manuscript.

**Table 1. Baseline characteristics of the pretraining cohorts included in this study. (p < 0.0001 for all pairwise comparisons of age, sex, and mortality).**

|  | BIDMC^∗^ | CODE^∗^ | SHZS^∗^ | VUMC^∗^ |
| --- | --- | --- | --- | --- |
| Location | US | Brazil | China | US |
| Care level | secondary | Primary | Primary | Secondary |
| Patients^∗^ | 127,041 | 424,577 | 420,956 | 252,294 |
| ECGs | 1,106,886 | 1,123,903 | 1,560,551 | 1,411,929 |
| Age mean | 64.46 | 56.57 | 54.15 | 60.21 |
| Age IQR | 21.09 | 24.00 | 26.00 | 22.24 |
| Male | 571,944 (52%) | 437,188 (39%) | 874,969 (56%) | 751,040 (54%) |
| Mortality (5y)** | 236,294 (35%) | 5,125 (16%) | _ | 89,900 (32%) |
| Hispanic | 7,077 | − | − | − |
| White | 84,265 | − | − | − |
| Black | 17,778 | − | − | − |
| Asian | 5,315 | − | − | − |
| Other | 12,606 | − | − | − |

∗ Pretraining cohort with patients having more than one ECG

** Includes only ECGs with five-year follow-up

**Table 2. Baseline characteristics of the cohorts explored for the downstream supervised tasks (BIDMC, CODE, SHZS, UKB, and PTB-XL). p < 0.0001 for all pairwise comparisons of age, sex, and mortality.**

|  | BIDMC | CODE | SHZS | VUMC | UKB | PTB-XL |
| --- | --- | --- | --- | --- | --- | --- |
| Location | US | Brazil | China | US | UK | Germany |
| Care level | secondary | primary | Primary | secondary | primary | Secondary |
| Patients^∗^ | 189,542 | 1,503,229 | 1,117,197 | 526,017 | 66,402 | 18,869 |
| ECGs | 1,169,387 | 2,202,555 | 2,257,485 | 1,685,652 | 70,655 | 21, 799 |
| Age mean | 63.73 | 53.54 | 53.62 | 58.76 | 65.31 | 62.77 |
| Age IQR | 21.67 | 25.00 | 26.00 | 23.73 | 12.00 | 22.00 |
| Male | 599,731 (51%) | 878,668 (40%) | 1,235,353 (55%) | 876,790 (53%) | 34,265 (49%) | 11,354 (52%) |
| Mortality (5y)* | 242,085 (35%) | 12,575 (9%) | - | 104,130 (26%) | 68 (0.4%) | - |
| Hispanic | 10,248 | − | − |  | − | − |
| White | 123,063 | − | − |  | − | − |
| Black | 24,251 | − | − |  | − | − |
| Asian | 8,924 | − | − |  | − | − |
| Other | 23,056 | − | − |  | − | − |

* Includes only ECGs with five-year follow-up.

**Manuscript Page 9, Line 182 (Results):**

*‘The 5-year mortality (among patients with 5-year follow-up) for the secondary care cohorts, BIDMC and VUMC, is 35% and 32%, respectively, compared with15% for the CODE cohort.’*

**Manuscript Page 9, Line 181 (Results):**

*‘The 5-year mortality among patients with five-year follow-up in the labelled cohort is 35% for BIDMC, 26% for VUMC, 9% for CODE, and 0.4% for the UKB. ‘*

**9. "This suggests that our random sampling from patient ECGs at the training time can successfully exploit the diversity in ECGs belonging to one patient."
- I would want to see more evidence behind this claim.**

Thank you for this insightful comment and for encouraging us to further substantiate this claim. We agree that the original statement was based on an observation and requires stronger empirical support.

In our initial experiments, we observed that the number of patients in BIDMC (127,041) is approximately half that of VUMC (252,306), while the total number of ECGs shows a smaller difference (1.2×: 1,106,886 vs 1,412,012, respectively). Despite this, pretraining on both datasets resulted in comparable downstream performance. One possible explanation is that, in BIDMC, multiple ECGs per patient are more effectively utilised. BIDMC also has a higher mortality rate. During training, each epoch samples two random ECGs per patient, allowing the model to potentially observe different ECG instances from the same patient across epochs. This may help expose intra-patient variability and thereby improve representation learning.

However, as you rightly point out, this observation alone is insufficient to conclude that intra-patient ECG diversity is the driving factor. Other differences between the datasets, such as outcome prevalence (for example, mortality rates of 35% in BIDMC vs 32% in VUMC), or other unmeasured cohort characteristics, may also contribute to the similar performance.

To directly address this concern, we conducted an additional experiment on BIDMC in which the same two ECGs per patient are fixed across all training epochs, thereby removing the effect of random sampling. We then compared the downstream performance of this model with our original approach, which samples different ECGs in each epoch. This experiment allows us to more explicitly evaluate whether exploiting intra-patient ECG diversity contributes to performance gains. The results of this comparison are now included in the Supplementary Information.

Table S6 compares the training loss trajectories of CAPE-B and CAPE-B̂ on the BIDMC dataset. CAPE-B is trained using randomly sampled patient ECG pairs at each epoch, whereas CAPE-B̂ uses the same fixed random pairs throughout training. Around 50 epochs, the loss for CAPE-B̂ begins to plateau, while CAPE-B continues to improve steadily. The downstream performance of the learned representations is further assessed using supervised models for age prediction, sex classification, and five-year mortality prediction (Table S4). Across all tasks, CAPE-B consistently outperforms CAPE-B̂.

**Fig. S6. Loss comparison for CAPE-B trained with random ECG pairs per epoch versus a model trained with a fixed random pair (CAPE-B̂). Losses are similar initially but diverge after ~50 epochs, with CAPE-B continuing to decrease while the fixed-pair model saturates.**

**Table S4. Performance comparison of supervised MLP models trained on BIDMC labels using features from CAPE-B (random ECG pairs per epoch) versus CAPE-B̂ (fixed pair). CAPE-B consistently outperforms CAPE-B̂.**

| No. | Model | Age (MAE) | Sex (AUC) | Mortality 5 year (AUC) |
| --- | --- | --- | --- | --- |
| 1 | CAPE-B | **7.95** | **0.927** | **0.854** |
| 2 | CAPE-B̂ | 8.39 | 0.903 | 0.815 |

**10. I would have talked about your reduced LVEF and five-year mortality tasks more in the discussion.**

Thank you for this helpful suggestion. We agree that these clinically relevant labels are of particular interest to the medical community. Age and sex were primarily used for performance comparison, as these demographic labels are consistently available across all cohorts, whereas cardiac labels are only available for a smaller subset.

Our results show that the same performance deterioration observed for CODE and SHZS in CAPE-X is also present for the cardiac labels. In contrast, CAPE-Z mitigates this performance loss and improves generalisation. To highlight this, we have expanded the Discussion section as follows:

**Manuscript Page 9, Line 197 (Results):**

*‘Supervised tasks implemented in the current work include predicting age and sex, as well as reduced left ventricular ejection fraction and five-year mortality. Demographic labels such as age and sex are available across all cohorts and have been shown to be implicitly encoded in ECG signals^4, 20^, enabling broader comparison while remaining sufficiently challenging and representative. In contrast, clinical labels are heterogeneous across datasets and not uniformly available; thus, we use these tasks to further corroborate the primary findings from age and sex.’*

**Manuscript Page 20, Line 447 (Discussion):**

*‘The demographic labels of age and sex were selected based on their availability across all cohorts for performance assessment. We further evaluated performance using cardiac labels, including reduced ejection fraction and five-year mortality, in cohorts with available data. The IDB approach outperformed in external validation, and the same performance trends observed for age and sex were also present for these cardiac labels. We also explored sex-stratified performance for five-year mortality and did not observe consistent subgroup-specific differences between CAPE-X and CAPE-Z. This suggests that the improvements associated with the IDB strategy are not driven by demographic subgroup effects but are more likely related to improved robustness to cohort-level distribution shifts. The observed variability across cohorts appears to be influenced by factors such as outcome prevalence and dataset-specific characteristics rather than model bias towards a particular subgroup.’*

**11. "A comparable work explores domain generalisation for ECG datasets from different sources"
- References are sometimes sprinkled in disruptively? The work could generally benefit from a little more informational organization.**

Thank you for this helpful observation. We agree that the placement of references and the organisation of the discussion could be improved for clarity and readability. In particular, the citation to related work on domain generalisation was previously introduced somewhat abruptly. To address this, we have revised the Discussion and Introduction sections throughout to improve the logical flow by grouping related work together and introducing it with clearer context. We now explicitly position prior work on ECG domain generalisation before contrasting it with our approach, and we have adjusted the placement of references to avoid disrupting the narrative.

For example, the above text is now revised as:

**Manuscript Page 19, Line 413 (Discussion):**

*‘We explore the impact of pretraining data composition on OOD test performance. Prior work on ECG domain generalisation has explicitly treated data from different hospitals or sources as distinct domains, demonstrating the presence of substantial distributional shifts and corresponding performance degradation under such shifts^28^. Building on this, we evaluate downstream generalisation for our CAPE model trained on diverse cohorts. ‘*

**Manuscript Page 5, Line 92 (Introduction):**

*‘Foundation models learn general patterns from large datasets, which enhances performance, accelerates learning, and reduces the need for extensive task-specific annotations5. While they can be pretrained with supervised learning6, self-supervised learning (SSL) has become the predominant paradigm, with common approaches including contrastive learning7 and masked modeling8. These SSL methods enable models to learn rich, transferable representations that generalise across a wide range of tasks and domains.’*

**Manuscript Page 6, Line 108 (Introduction):**

*‘In computer vision, prior work has shown that dataset composition can affect representations learned through contrastive self-supervised learning, while also demonstrating a degree of robustness across different dataset types16. Similarly, in the ECG domain, a prior study using self-supervised pretraining across medium-sized secondary-care datasets reported comparable out-of-distribution performance despite substantial distributional differences between cohorts17. However, it remains unclear whether these observations generalise to datasets derived from different healthcare levels with varying diversity and size. This question is particularly relevant in ECG analysis, where signals from healthy individuals are often relatively homogeneous, while pathological conditions introduce structured but heterogeneous deviations. A large-scale systematic investigation of ECG data is therefore needed.’*

**12. "We anticipate that training the prediction head on the full cohort, followed by fine-tuning the model, could further improve performance."
- This is true all of ML. Consider removing.**

Thank you for this comment. We agree that the statement is general and reflects a common observation in machine learning regarding the benefits of additional supervised fine-tuning. To address this, we have removed the sentence from the manuscript to avoid overgeneral and non-specific claims that do not directly contribute to the discussion of our method or results.

**13. "The ECGs for the same patient are treated as positive views while all other ECGs in the batch are negative views."
- Is this inspired by another paper? You don't seem to motivate this choice of contrastive method anywhere, and this study begs the question: Would your results hold across other forms of contrastive learning? E.g., same-sample pairs (temporal crops), or other options. Feels like this should be discussed more.**

Thank you for highlighting this important point regarding motivation and clarity. We agree that the rationale for the contrastive design should have been more explicitly signposted in the manuscript.

Our approach is based on Patient Contrastive Learning (PCLR)^1^, where ECGs from the same patient are treated as positive pairs and all other ECGs within the batch are treated as negatives. This design is motivated by the assumption that recordings from the same patient share clinically meaningful structure and has been shown to yield strong representations for ECG modelling.

Importantly, this formulation is not newly introduced in the present work but follows our previously published CAPE framework². PCLR was originally proposed by Diamant et al.^1^, who demonstrate its effectiveness for ECG representation learning, while our CAPE framework² introduces additional augmentations and training modifications. The current manuscript builds on CAPE and applies it within our experimental setting rather than redefining the contrastive objective.

We acknowledge that this connection was not sufficiently emphasised in the original manuscript and have revised the text to clarify this point.

References

[1] Diamant N, et al. Patient contrastive learning: a performant, expressive, and practical approach to electrocardiogram modeling. PLoS Comput Biol. 2022;18:e1009862.

[2] Khattak GR, et al. Contrasting by augmented patient electrocardiograms to learn representations for a foundation model. In: Bellazzi R, Juarez Herrero JM, Sacchi L, Zupan B, editors. Artificial Intelligence in Medicine (AIME 2025). Lect Notes Comput Sci. 2025;15735.

Accordingly, we have updated the manuscript as follows:

**Manuscript Page 25, Line 550 (Methods):**

*‘We follow the patient contrastive learning (PCLR)^8^, where ECGs from the same patient are treated as positive pairs, while all other ECGs in the batch are treated as negatives.’*

It is possible that alternative contrastive learning approaches based on augmentations of the same signal may similarly capture cohort-specific artefacts, particularly when negative samples are drawn randomly from a multi-centre cohort. While a systematic investigation of these effects is beyond the scope of the present study, we acknowledge this as a limitation and discuss it accordingly in the manuscript.

**Manuscript Page 22, Line 484 (Limitations):**

*‘While the insights gained are expected to be relevant to other contrastive learning frameworks and supervised training approaches, further experimentation is required to validate these findings in different contexts, including alternative foundation model paradigms such as masked autoencoders and generative pretraining methods.’*

**14. I found the supposed ethnicity clustering within Figure 4b confusing.**

We apologise for the confusion regarding the ethnicity visualisation. To improve clarity, we have revised Figure 4 and split it into two separate figures. The original combined visualisation in previous Figure 4B has been separated into (A) cohort-level t-SNE representations and (B) ethnicity-level t-SNE representations for the BIDMC cohort, as ethnicity labels are only available for this dataset.

The aim of the analysis was to assess whether the learned representations exhibit clustering by ethnicity, motivated by prior work suggesting that ethnicity may be associated with subtle ECG signatures. In the original combined plot, cohort structure and ethnicity were overlaid, which made interpretation difficult.

In the revised figures, cohort structure is shown separately from ethnicity structure. The cohort-level plot illustrates inter-dataset organisation, while the BIDMC-only plot shows ethnicity stratification. In this updated visualisation, we observe that ethnicity groups within BIDMC do not form distinct clusters, suggesting that the learned representations do not meaningfully separate by ethnicity in this embedding space.

Fig 4. t-SNE visualization of learned embeddings from the CAPE-X model form clusters across cohorts. The CAPE-X model was pretrained on the combined BCSV dataset using random batches. (A) The learned feature representation, projected onto the first two components, shows distinct clustering by cohort, with overlap observed between BIDMC and PTB-XL. (B) Within the BIDMC cohort, features from different ethnic groups exhibit substantial overlap, with low distributional separation in the component space (MMD = 0.007). (C) In contrast, features from the CODE dataset form two clearly separated clusters corresponding to recording device types, indicating strong distributional shift in the component space (MMD = 0.873).

**15. Typo in Figure 6.**

Thank you for pointing this out. We have corrected the typographical error in Figure 6 in the revised manuscript.

Fig 7. Overview of the CAPE pretraining framework. Patient A has two ECG recordings (ECG1A and ECG2A), and Patient B, another patient in the same batch, has ECG1B and ECG2B. Each ECG undergoes temporal augmentations before being encoded by the network into a 256-dimensional feature vector. The contrastive loss encourages embeddings from the same patient’s ECGs to be closer together while pushing apart embeddings from different patients within the batch.

**Reviewer 3:**

**I appreciate the scale of this work. The effort to systematically evaluate and explor how cohort composition impacts contrastive pretraining in ECG-based foundation models. The findings related to distribution shift and OOD performance are particularly interesting as well. A couple of suggestions are made to straighten the presentation.**

We thank the reviewer for their positive assessment of our work and for recognising the importance of systematically evaluating cohort composition in contrastive pretraining for ECG-based foundation models. We are glad that the analysis of distribution shift and out-of-distribution performance was found to be particularly interesting. We address the reviewer’s specific suggestions in detail below.

**1. Relatedly, the review/discussion would benefit from positioning this work within the rapidly evolving foundation model literature across other biomedical modalities. Recent studies have demonstrated that contrastive pretraining strategies and ensemble-based foundation models can meaningfully improve robustness to out-of-distribution samples (Zhao, J., et al. Uncertainty-aware ensemble of foundation models differentiates glioblastoma from its mimics. Nat Commun 16, 8341 (2025).** [**https://doi.org/10.1038/s41467-025-64249-6**](https://doi.org/10.1038/s41467-025-64249-6)**), and contrastive learning has been applied to enhance fairness in AI systems (Lin, S. Y., et al. (2025). Contrastive learning enhances fairness in pathology artificial intelligence systems. Cell reports. Medicine, 6(12), 102527.** [**https://doi.org/10.1016/j.xcrm.2025.102527**](https://doi.org/10.1016/j.xcrm.2025.102527)**). These works suggest that cohort-aware pretraining and contrastive strategies may have broader implications for multimodal clinical AI beyond signal-based applications. Similar conclusions are drawn, so they should be added to the introduction or discussion.**

We thank the reviewer for this helpful suggestion. We agree that situating our work within the broader foundation model literature across biomedical modalities strengthens the discussion. We have therefore expanded the Introduction/Discussion to incorporate the suggested studies and to better highlight related advances in ensemble-based foundation models and fairness-aware contrastive learning, particularly in addressing distribution shift and robustness to out-of-distribution data.

**Manuscript Page 7, Line 131 (Introduction):**

*‘Foundation models must overcome domain shift to be clinically viable, a challenge observed across multiple medical modalities. For example, a retinal image-based foundation model trained on British patient data demonstrated poor transferability to Chinese cohorts24. In parallel, evidence of socio-demographic bias has also been reported in foundation models used for medical diagnosis based on large language models23, highlighting broader concerns regarding fairness and robustness across diverse populations. Together, these findings underscore the critical need for robust generalisation across diverse populations to ensure the practical utility of foundation models in biomedical applications.*

*Several approaches have been proposed to improve domain generalisation²⁵ across diverse domains. Uncertainty-aware and ensemble-based foundation models²⁶ improve external validation performance in glioblastoma classification by leveraging multiple models and uncertainty estimation to better distinguish tumours from their mimics. Contrastive learning with fairness objectives²⁷ enhances fairness in pathology AI systems by incorporating fairness constraints into the learning objective. For ECG tasks, self-supervised contrastive learning¹⁷ and aggregation of features from multiple intermediate layers²⁸ have been crucial for improving generalisation, collectively highlighting the need for models that generalise across diverse clinical environments.’*

**Manuscript Page 16, Line 348 (Discussion):**

*‘Such limitations have motivated approaches to improve generalisation, including the use of intermediate-layer features from residual networks28, ensemble-based foundation models25, and fairness-aware contrastive learning26. Although the current work focuses on contrastive learning, cohort-dependent effects on representation learning and downstream generalisation may also extend to alternative foundation model paradigms, including masked autoencoders and generative pretraining approaches.’*

**2. The current downstream evaluation relies predominantly on age and sex prediction tasks, which, while useful as technical benchmarks, are not strongly representative of clinically meaningful deployment scenarios. If the goal is to support claims regarding generalizability of medical foundation models, some additional downstream analyses would strengthen the translational relevance of the work, for example, incorporating broader clinical prediction tasks or fairness-sensitive outcomes beyond demographic inference.**

**Incorporating discussion of these recent developments would help contextualize the present findings and highlight how future multimodal or ensemble-based integration strategies could further improve robustness and fairness in foundation models for clinical deployment.

For these reasons, I would suggest a revision to include additional downstream analysis (clinically relevant tasks in ECG, e.g., pattern classifications) and to better situate the work within the broader landscape of recent foundation model research in biomedicine.**

Thank you for this helpful suggestion. We agree that relying solely on age and sex prediction may limit the relevance of downstream evaluation when assessing the generalisability of medical foundation models. In the revised manuscript, we clarify that, in addition to demographic prediction tasks, we also evaluated clinically relevant outcomes including reduced left ventricular ejection fraction and five-year mortality to assess generalisation across external cohorts. These tasks are more directly aligned with real-world clinical deployment scenarios. We additionally note these tasks explicitly at the beginning of the Results section to improve clarity.

The demographic tasks were primarily intended as broadly comparable benchmark tasks across cohorts and models, analogous to a “canary in a coal mine” for assessing distributional sensitivity and representation learning. Age and sex labels were consistently available across all datasets, enabling systematic cross-cohort comparison, whereas clinical labels were heterogeneous and not uniformly available. Importantly, we observed that the same trends identified using demographic labels were also reflected in the cardiology-related downstream tasks, supporting the broader conclusions regarding model generalisation.

The following text has been added to the Results section:

**Manuscript Page 10, Line 206 (Results):**

*‘Supervised tasks implemented in the current work include predicting age and sex, as well as reduced left ventricular ejection fraction and five-year mortality. Demographic labels such as age and sex are available across all cohorts and have been shown to be implicitly encoded in ECG signals^4, 20^, enabling broader comparison while remaining sufficiently challenging and representative. In contrast, clinical labels are heterogeneous across datasets and not uniformly available; thus, we use these tasks to further corroborate the primary findings from age and sex.’*

We also appreciate the reviewer highlighting recent approaches aimed at improving robustness and mitigating bias in foundation models. In response, we incorporated discussion of ensemble-based foundation models and fairness-aware contrastive learning, as these represent promising directions for improving transferability and robustness in real-world clinical settings.

The following text has been added:

**Manuscript Page 7, Line 140 (Introduction):**

*‘Several approaches have been proposed to improve domain generalisation²⁵ across diverse domains. Uncertainty-aware and ensemble-based foundation models²⁶ improve external validation performance in glioblastoma classification by leveraging multiple models and uncertainty estimation to better distinguish tumours from their mimics. Contrastive learning with fairness objectives²⁷ enhances fairness in pathology AI systems by incorporating fairness constraints into the learning objective. For ECG tasks, self-supervised contrastive learning¹⁷ and aggregation of features from multiple intermediate layers²⁸ have been crucial for improving generalisation, collectively highlighting the need for models that generalise across diverse clinical environments.’*

**Manuscript Page 16, Line 348 (Discussion):**

*‘Such limitations have motivated approaches to improve generalisation, including the use of intermediate-layer features from residual networks28, ensemble-based foundation models25, and fairness-aware contrastive learning26. Although the current work focuses on contrastive learning, cohort-dependent effects on representation learning and downstream generalisation may also extend to alternative foundation model paradigms, including masked autoencoders and generative pretraining approaches.’*

**Reviewer 4:**

**1. This manuscript presents a systematic investigation of how pretraining cohort composition influences the performance and out-of-distribution (OOD) generalisation of the CAPE contrastive learning framework for ECG foundation models. The study is conducted at impressive scale, utilizing over 5.2 million ECGs from five countries across four continents. The authors additionally propose an In-Distribution Batch (IDB) strategy to mitigate cohort-specific artifact encoding. The topic is timely and clinically relevant. However, several methodological concerns and gaps in experimental validation must be addressed before the manuscript is suitable for publication.**

We sincerely thank the reviewer for the thoughtful and encouraging assessment of our work. We appreciate the recognition of the scale of the study, the clinical relevance of the problem, and the importance of understanding how pretraining cohort composition affects out-of-distribution generalisation in ECG foundation models. We are also grateful for the reviewer’s constructive feedback regarding the methodological concerns and areas requiring further experimental clarification.

Below, we respond to each point individually and describe the corresponding revisions made to the manuscript.

**My major comments are:

1) The downstream evaluation is restricted to age regression and sex classification. They might be insufficient to demonstrate the clinical utility of the proposed IDB strategy. The authors can consider include at least one clinically meaningful prediction task.**

We thank the reviewer for this important suggestion and agree that clinically meaningful downstream tasks are important for demonstrating the relevance of the proposed IDB strategy. While age regression and sex classification were used as broadly comparable benchmark tasks across all cohorts due to their consistent availability, the current study also includes clinically relevant prediction tasks, specifically reduced left ventricular ejection fraction and five-year mortality, for evaluation on external cohorts. We recognise that these tasks were not sufficiently highlighted in the original manuscript and have revised the text to make this clearer.

The demographic tasks were intentionally used as sensitive indicators of representation quality and cohort-related distributional effects, analogous to a “canary in a coal mine,” because prior work has demonstrated that age and sex are strongly encoded within ECG signals while remaining consistently available across datasets. Importantly, we observed that the same generalisation trends identified using demographic labels were also reflected in the clinically relevant cardiology tasks, further supporting the utility of the proposed IDB strategy.

To clarify this point, we added the following text at the beginning of the Results section:

**Manuscript Page 10, Line 206 (Results):**

*‘Supervised tasks implemented in the current work include predicting age and sex, as well as reduced left ventricular ejection fraction and five-year mortality. Demographic labels such as age and sex are available across all cohorts and have been shown to be implicitly encoded in ECG signals^4, 20^, enabling broader comparison while remaining sufficiently challenging and representative. In contrast, clinical labels are heterogeneous across datasets and not uniformly available; thus, we use these tasks to further corroborate the primary findings from age and sex.’*

**2) Downstream evaluations rely on frozen features extracted from the pretrained backbone, with only the MLP prediction head trained for each task. The authors acknowledge this limitation in the limitations section, but its implications are substantial. Fine-tuning the backbone could potentially diminishing or reversing the observed OOD benefits of the IDB strategy.**

We thank the reviewer for this important point and for highlighting the implications of evaluating downstream tasks using frozen representations. We agree that the backbone is not fine-tuned in our experimental setup. However, this is a deliberate and widely adopted design choice in representation learning studies. Our primary objective is to compare pretraining strategies and evaluate the quality of the learned representations, rather than to maximise performance on a particular downstream task. Full fine-tuning introduces an additional layer of task-specific optimisation, which can obscure differences originating from the pretraining stage and make it more difficult to attribute performance gains to the proposed IDB strategy.

Moreover, foundation models are often used in practice as feature extractors, where pretrained representations are transferred to downstream analyses and prediction tasks without updating the backbone. In many real-world settings, full fine-tuning is computationally expensive and not always feasible, particularly for large-scale models and datasets.

In our experiments, downstream evaluation is performed by training an MLP prediction head on frozen backbone features, which closely follows the standard linear probing paradigm. This setup isolates the information encoded in the pretrained representations from downstream adaptation effects, enabling a more controlled and task-agnostic assessment of representation quality. Although our prediction head includes a lightweight non-linearity, prior work in self-supervised and contrastive learning (e.g., SimCLR, MoCo, and BYOL) has shown that such shallow heads remain consistent with linear-probe-style evaluation and are sufficient to reflect the utility of learned features.

We also note that frozen-feature evaluation offers important practical advantages in our setting. It substantially reduces computational cost and enables consistent large-scale evaluation across multiple cohorts and downstream tasks. Given the scale of our experiments and the number of comparisons performed, this design helps ensure that the observed differences primarily reflect the effects of pretraining rather than variability introduced by downstream optimisation dynamics.

Finally, we acknowledge that full fine-tuning could potentially alter the magnitude of the observed out-of-distribution effects. To address this, we evaluated fine-tuned CAPE-X and CAPE-Z models in Supplementary Table S4 and found results consistent with the frozen-feature setting, with CAPE-X still exhibiting reduced external performance after fine-tuning.

We have clarified this rationale in the revised manuscript as follows:

**Manuscript Page 9, Line 197 (Results):**

*‘Supervised learning is performed in a manner similar to a linear probe, except that a non-linear multi-layer perceptron (MLP) prediction head is used. Linear probing is a standard methodology to compare different pretraining approaches and assess the quality of learned representations without any bias introduced by downstream optimisation (e.g., SimCLR^12)^. Moreover, the training time is substantially reduced by leveraging precomputed features, which is essential given the scale of the experiments.’*

We hope this clarification addresses the reviewer’s concern regarding the implications of frozen backbone evaluation and the rationale behind our experimental design.

**3) A key mechanistic finding of the paper is that t-SNE clusters in CAPE-X representations correspond more strongly to ECG acquisition device differences than to patient ethnicity. This is an important and novel observation. However, the authors do not provide experiment that disentangles the contribution of device-level artifacts from the benefit of IDB. Specifically, it remains unclear whether standard signal harmonization or device-aware preprocessing could achieve similar or superior OOD improvements without requiring a modified training objective.**

We thank the reviewer for this important point. While we agree that our current experiments do not fully disentangle the relative contributions of device-specific artefacts and the IDB training objective, our analyses suggest a strong association between cohort-specific representation structure and downstream generalisation performance. Specifically, the model exhibiting stronger clustering by cohort and acquisition source also demonstrated reduced performance across several external cohorts, whereas the IDB-based model showed markedly reduced clustering alongside more robust external validation performance. Together, these findings suggest that suppressing cohort-specific shortcuts may improve robustness under distribution shift. Importantly, however, the relationship between clustering behaviour and downstream performance degradation was not uniform across cohorts. Although most cohorts formed distinct clusters, only a subset exhibited substantially impaired generalisation. This observation suggests that the impact of encoded cohort-specific signals likely depends on the nature and magnitude of the underlying distributional differences.

We also note that identical preprocessing pipelines were applied across all cohorts, indicating that the observed differences are unlikely to be driven by preprocessing variability and instead reflect acquisition- and cohort-specific characteristics.

Regarding whether standard signal harmonisation or device-aware preprocessing could achieve similar or superior out-of-distribution (OOD) improvements, we acknowledge this as an important and valid alternative hypothesis. In this study, we applied consistent preprocessing across datasets but did not explicitly incorporate device-specific harmonisation, primarily because such approaches typically require detailed and reliable device metadata, which is not consistently available across all cohorts. In addition, the proposed IDB framework is designed to operate without any explicit device annotations or harmonisation parameters, making it applicable in settings where such information is incomplete or unavailable.

To further explore the role of acquisition-related variability, we conducted additional analyses in the Supplementary Information using ECGs acquired from different devices within the CODE cohort. We identified systematic spectral differences between devices (Supplementary Fig. S2), which were not visually apparent in the raw signals (Supplementary Fig. S1). We further observed that spectral alignment reduced the clustering patterns in the learned representation space. These findings indicate that harmonisation (Supplementary Figs. S3–S5) can mitigate part of the observed device-related structure; however, they do not directly establish whether such approaches would match or exceed the out-of-distribution improvements achieved by IDB, which remains an interesting direction for future work.

Overall, we view device-aware harmonisation and IDB as complementary approaches: harmonisation explicitly targets known acquisition differences, whereas IDB aims to reduce reliance on cohort-specific shortcuts more broadly without requiring explicit metadata or modifications to the input preprocessing pipeline.

**Supplementary Figures:**

**Fig S1. Two representative ECGs from the CODE dataset. (A) ECG recorded with device A. (B) ECG recorded with device B. No obvious artifacts are observed.**

**Fig. S2. Power spectral density (PSD) of ECGs recorded with devices A and B, showing subtle differences, including a small peak around 100 Hz in device B.**

**Fig. S3. A) A representative ECG recorded with device A. B) The same ECG after transformation to match the power spectral density (PSD) of device B.**

**Fig. S4. Power spectral density (PSD) of an ECG recorded with device A and the same ECG after spectral transformation to match device B.**

**Fig. S5. t-SNE components of CAPE embeddings for the CODE dataset. (A) Features from ECGs recorded by the two devices form distinct clusters. (B) Features from ECGs recorded by device A and those from device B transformed to match the spectrum of device A are not separated. (C) Features from ECGs recorded by device B and those from device A transformed to match the spectrum of device B are not separated. (D) Features from ECGs transformed to match the spectrum of the other device again form separate clusters.**

We also add the following to the manuscript:

**Manuscript Page 22, Line 495 (Limitations):**

*‘We further acknowledge that preprocessing-based strategies may offer a means to mitigate device-specific artefacts. However, exploring such approaches lies beyond the scope of the present study and represents an important avenue for future investigation. Notably, these methods typically require explicit identification of the underlying sources of variation being encoded, which may extend beyond device-related factors. In contrast, IDB does not rely on prior knowledge of these sources.’*

**Reviewer #5:**

**1. The paper's central claim that In Distribution Batch (IDB) training improves Out Of Distribution (OOD) generalization is interesting. However, it must be noted that this paper focuses on contrastive learning which is only one of many approaches for creating foundation models. I think there should be more commentary in the paper on other approaches for creating foundation models (liked masked autoencoders and generative pretraining) and whether these other approaches have similar challengs with respect to OOD generalization. Also, it would be useful to have a discussion on whether the IDB approach translates to these diffferent approaches for creating foundation models.**

We thank the reviewer for this thoughtful and important comment. We agree that contrastive learning represents only one class of approaches for developing foundation models, and that the broader implications of our findings for alternative pretraining paradigms should be discussed more explicitly.

Contrastive learning explicitly optimise representations by bringing positive pairs closer together while separating negatives. In heterogeneous multi-cohort datasets, this objective may inadvertently encourage the model to exploit cohort-specific or acquisition-specific features that help discriminate between samples, thereby contributing to reduced OOD generalisation. Our findings suggest that not only the learning framework itself, but also the composition of the pretraining cohorts, substantially influences the resulting representations.

We agree that other foundation model paradigms, including masked autoencoders and generative pretraining approaches, may also be susceptible to learning spurious cohort- or device-specific features, particularly given that these methods aim to reconstruct or model raw ECG signals directly. However, systematically evaluating whether such methods exhibit similar OOD behaviour, and whether IDB-like strategies would yield comparable benefits, would require a controlled comparative study under matched cohorts and evaluation settings. We view this as an important direction for future work, but beyond the scope of the present study. More generally, the IDB strategy is most naturally aligned with representation-learning frameworks that rely on similarity-based objectives, whereas reconstruction-based approaches may require alternative mechanisms to discourage learning of non-causal, cohort-specific features.

To address the reviewer’s suggestion, we have expanded the discussion and limitations sections to better contextualise the findings within the broader landscape of foundation model approaches and to acknowledge the need for future investigations across alternative pretraining paradigms.

**Manuscript Page 5, Line 92 (Introduction):**

*‘Foundation models learn general patterns from large datasets, which enhances performance, accelerates learning, and reduces the need for extensive task-specific annotations^5^. While they can be pretrained with supervised learning6, self-supervised learning (SSL) has become the predominant paradigm, with common approaches including contrastive learning^7^ and masked modeling^8^. These SSL methods enable models to learn rich, transferable representations that generalise across a wide range of tasks and domains.’*

**Manuscript Page 16, Line 348 (Discussion):**

*‘Although the current work focuses on contrastive learning, cohort-dependent effects on representation learning and downstream generalisation may also extend to alternative foundation model paradigms, including masked autoencoders and generative pretraining approaches.’*

**Manuscript Page 22, Line 484 (Limitations):**

*‘While the insights gained are expected to be relevant to other contrastive learning settings, further experimentation is required to validate these findings across different learning paradigms, including supervised learning and alternative foundation model approaches such as masked autoencoders and generative pretraining methods.’*

**2. The paper choses to use only age and sex as downstream finetuning benchmarks, but it is not clear how well the results translate to downstream tasks like arrhythmia detection. More commentary around this would be useful.**

We thank the reviewer for this important comment and agree that the role of the downstream tasks should be clarified more precisely. In this work, age and sex prediction are used as primary benchmark tasks because they are consistently available across all cohorts, enabling a fair and systematic comparison of representation quality and generalisation behaviour across pretraining strategies and datasets. Prior work has also shown that both age and sex are strongly encoded in ECG signals, making them well-established proxy tasks for evaluating learned representations at scale.

Importantly, we also evaluate clinically meaningful downstream tasks, including reduced left ventricular ejection fraction (LVEF) and five-year mortality prediction, to assess whether the trends observed in the demographic benchmarks translate to clinically relevant outcomes. We find that the generalisation patterns observed for age and sex are consistently reflected in these cardiac endpoints, suggesting that the conclusions are not restricted to demographic inference.

We acknowledge that these tasks were not previously introduced at the outset. We have therefore revised the manuscript to clarify this earlier and explicitly describe all downstream evaluation labels upfront, improving readability and ensuring that the evaluation setup is immediately transparent to the reader.

**Manuscript Page 10, Line 206 (Results):**

*‘Supervised tasks implemented in the current work include predicting age and sex, as well as reduced left ventricular ejection fraction and five-year mortality. Demographic labels such as age and sex are available across all cohorts and have been shown to be implicitly encoded in ECG signals^4, 20^, enabling broader comparison while remaining sufficiently challenging and representative. In contrast, clinical labels are heterogeneous across datasets and not uniformly available; thus, we use these tasks to further corroborate the primary findings from age and sex.’*

**3. The decision to use frozen features (and no fine-tuning of the feature-extracting backbone fine-tuning) limits the conclusions. It is unclear whether the observed OOD degradation is a property of the pretraining strategy itself or simply an artifact of not adapting the feature-extracting backbone to downstream tasks. The authors acknowledge this as a limitation, but it deserves more emphasis, since fine-tuning might largely resolve the OOD gap.**

**key aspect of the experimental design that deserves clearer emphasis.**

We thank the reviewer for highlighting this important point and agree that it warrants clearer emphasis, as it is central to the experimental design. In our study, the backbone is intentionally kept frozen. The primary objective is to assess how pretraining cohort composition and the proposed IDB strategy affect the quality and transferability of the learned representations, rather than to optimise performance on individual downstream tasks. Allowing full fine-tuning would introduce an additional layer of task-specific adaptation that can obscure the effects of the pretraining strategy, making it more difficult to attribute observed differences specifically to representation learning.

Evaluating frozen features isolates the information encoded during pretraining and enables a controlled and consistent comparison across cohorts, tasks, and experimental settings. This is a standard evaluation paradigm in representation learning and self-supervised learning, where linear probing or lightweight heads are used to assess representation quality independent of downstream optimisation. Importantly, this setting is also practically relevant, as many applications of foundation models rely on frozen or minimally adapted representations due to the computational cost and deployment constraints associated with full fine-tuning.

In response to the reviewer’s concern, we additionally report fine-tuning results in the Supplementary Information (Table S6). These results indicate that fine-tuning does not fully mitigate the reduced generalisation observed for models pretrained on multi-centre cohorts, suggesting that the observed OOD gap is not solely an artefact of freezing the backbone.

We have revised the manuscript to more explicitly emphasise the rationale for frozen-feature evaluation and to better contextualise the supplementary fine-tuning analysis.

**Manuscript Page 9, Line 197 (Results):**

*‘Supervised learning is performed in a manner similar to a linear probe, except that a non-linear multi-layer perceptron (MLP) prediction head is used. Linear probing is a standard methodology to compare different pretraining approaches and assess the quality of learned representations without any bias introduced by downstream optimisation (e.g., SimCLR^8^). In addition, representations learned by foundation models are often adapted to downstream applications using lightweight prediction heads rather than computationally expensive end-to-end fine-tuning. In our experiments, leveraging precomputed features substantially reduced training time, which was essential given the scale of the study.’*

**Manuscript Page 14, Line 310 (Results):**

*‘We further evaluated fine-tuned models (Supplementary Information), in which the CAPE feature-generating backbone was also updated during training. The performance deterioration observed for CAPE-X in external validation persisted after fine-tuning, whereas CAPE-Z demonstrated robust generalisation across cohorts.’*

**Manuscript Page 20, Line 445 (Discussion):**

*‘A similar pattern is also seen in fine-tuned models (Supplementary Table S5)’.*

**Supplementary Table S5:**

Table S5. **Performance comparison on fine-tuned foundation models pretrained using random-batch and in-distribution batch (IDB) strategies, evaluated using the AUC.** The fine-tuning is performed on BIDMC labels across three independent runs, with evaluation conducted on 10,000 random test samples from external cohorts. Performance metrics are reported as mean values with standard errors (SE) in parentheses; for example, a mean absolute error (MAE) of 7.41 with an SE of 0.004 is reported as 7.41(004), and an area under the curve (AUC) of 0.954 with an SE of 0.0002 as 0.954(002).

| No. | Model | BIDMC | CODE | SHZS | UKB | PTB-XL | mean |
| --- | --- | --- | --- | --- | --- | --- | --- |
| Age prediction (MAE) | | | | | | | |
| 1 | CAPE-X | **7.41(004)** | 30.94(*) | 12.15(*) | **5.77(016)** | 7.22(051) | 12.70 |
| 2 | CAPE-Z | 7.44(003) | **7.95(047)** | **7.42(022)** | 5.83(015) | **7.03(029)** | **7.13** |
| 3 | p-value^!^ | 0.919 | <0.0001 | <0.0001 | <0.0001 | 0.063 | - |
| Sex prediction (AUC) | | | | | | | |
| 4 | CAPE-X | 0.954(002) | 0.715(*) | 0.900(104) | 0.980(008) | 0.934(013) | 0.896 |
| 5 | CAPE-Z | **0.955(000)** | **0.931(000)** | **0.955(001)** | **0.983(000)** | **0.938(001)** | **0.952** |
| 6 | p-value^$^ | 0.038 | <0.0001 | <0.0001 | 0.0132 | 0.002 | **-** |

* Out of range with SE(MAE) ≥ 1.0 or SE(AUC) ≥ 0.1.

^!^ Wilcoxon signed-rank test on sample-wise MAE

^$^ DeLong test
