# Supplementary material for: Data distribution impacts the performance and generalisability of contrastive learning-based foundation models of electrocardiograms
Source: PLOS Digit Health. 2026 Sep 2;5(9):e0001623. doi: 10.1371/journal.pdig.0001623 (PMC13537565; doi:10.1371/journal.pdig.0001623)
Supplement: Peer review history

# Peer review: Decision Letter 0

**DOI:** 10.1371/journal.pdig.0001623.r001

**Authors:** Jie-Zhi Cheng, Zhenwei Shi

10 Mar 2026

Response to Reviewers Revised Manuscript with Track Changes Manuscript **Journal Requirements:**

1. Please send a completed 'Competing Interests' statement, including any COIs declared by your co-authors. Please declare all competing interests beginning with the statement “I have read the journal's policy and the authors of this manuscript have the following competing interests:”

For further assistance, you may go to: https://journals.plos.org/digitalhealth/s/competing-interests

2. We note that you have indicated that there are restrictions to data sharing for this study. For studies involving human research participant data or other sensitive data, we encourage authors to share de-identified or anonymized data. However, when data cannot be publicly shared for ethical reasons, we allow authors to make their data sets available upon request. For information on unacceptable data access restrictions, please see http://journals.plos.org/digitalhealth/s/data-availability#loc-unacceptable-data-access-restrictions.

Before we proceed with your manuscript, please address the following prompts:

a) If there are ethical or legal restrictions on sharing a de-identified data set, please explain them in detail (e.g., data contain potentially identifying or sensitive patient information, data are owned by a third-party organization, etc.) and who has imposed them (e.g., a Research Ethics Committee or Institutional Review Board, etc.). Please also provide contact information for a data access committee, ethics committee, or other institutional body to which data requests may be sent.

b) If there are no restrictions, please upload the minimal anonymized data set necessary to replicate your study findings to a stable, public repository and provide us with the relevant URLs, DOIs, or accession numbers. Please see http://www.bmj.com/content/340/bmj.c181.long for guidelines on how to de-identify and prepare clinical data for publication. For a list of recommended repositories, please see https://journals.plos.org/plosone/s/recommended-repositories. You also have the option of uploading the data as Supporting Information files, but we would recommend depositing data directly to a data repository if possible.

Please update your Data Availability statement in the submission form accordingly.

3. Please provide separate main figure files in .tif or .eps format only and ensure that all files are under our size limit of 10MB. You may leave the embedded figures in the manuscript.

For more information about how to convert your figure files please see our guidelines: https://journals.plos.org/digitalhealth/s/figures

4. If the reviewer comments include a recommendation to cite specific previously published works, please review and evaluate these publications to determine whether they are relevant and should be cited. There is no requirement to cite these works unless the editor has indicated otherwise.

**Additional Editor Comments (if provided):**

• Expand downstream evaluation to include clinically meaningful ECG tasks. (Reviewers 3, 4, 5)

• Assess the effect of backbone fine-tuning (or justify why frozen-feature evaluation is sufficient). (Reviewers 4, 5)

• Provide deeper analysis and explanation of the IDB strategy, including subgroup analysis and device-artifact considerations. (Reviewers 1, 4)

• Strengthen statistical evaluation, including full-cohort analyses where possible and clarification of multiple-comparison corrections. (Reviewers 1, 2)

• Clarify dataset definitions and cohort characteristics, particularly the definition of “healthy populations.” (Reviewer 1)

• Situate the work within broader foundation model research, including other pretraining strategies. (Reviewers 3, 5)

• Improve reproducibility and accessibility of the model or implementation details where possible. (Reviewer 1)

• Provide explicit fairness analyses if claims of clinical fairness are maintained. (Reviewer 1)

• Substantially improve figures, organization, and language quality throughout the manuscript. (Reviewers 1, 2)

**Reviewers' Comments:**

**Comments to the Author**

1. Does this manuscript meet PLOS Digital Health’s publication criteria?

Reviewer #1: Yes

Reviewer #2: Partly

Reviewer #3: Yes

Reviewer #4: Yes

Reviewer #5: Yes

**********

2. Has the statistical analysis been performed appropriately and rigorously?

Reviewer #1: Yes

Reviewer #2: Yes

Reviewer #3: Yes

Reviewer #4: Yes

Reviewer #5: Yes

**********

3. Have the authors made all data underlying the findings in their manuscript fully available (please refer to the Data Availability Statement at the start of the manuscript PDF file)?

The PLOS Data policy

Reviewer #1: Yes

Reviewer #2: Yes

Reviewer #3: Yes

Reviewer #4: Yes

Reviewer #5: Yes

**********

4. Is the manuscript presented in an intelligible fashion and written in standard English?

Reviewer #1: Yes

Reviewer #2: Yes

Reviewer #3: Yes

Reviewer #4: Yes

Reviewer #5: Yes

**********

Reviewer #1: The article demonstrates innovation and practicality in its methodological design, experimental scale, and research questions. However, despite its high overall quality, several shortcomings remain, which are recommended for improvement during the revision process:

1. Although the article used multiple international datasets for pre-training and downstream task validation, the explanation for the improved performance of the "CAPE-Z" model (using the IDB strategy) on external data remains somewhat general. Further exploration is needed to determine whether the IDB strategy is more effective in certain subgroups to enhance the transparency and interpretability of the mechanism.

2. The article repeatedly mentions "healthy populations," but the definition criteria for these groups are not clearly defined. Especially in the comparison between UKB and CODE, differences in health status may affect model performance. It is recommended to supplement the health screening criteria or relevant clinical indicators for these populations.

3. To ensure fairness in the comparison, performance evaluation metrics are derived from only a subset of 10,000 training samples and 2,000 test samples randomly selected from each cohort. Therefore, the reported performance results do not reflect the model's true performance on the full cohort dataset.

4. The t-SNE plot shows a significant clustering phenomenon in the features of the CAPE-X model, which the authors speculate is related to device type. However, quantitative analysis is lacking to support this conclusion.

5. While the IDB strategy performs well in practice, the article does not provide a theoretical analysis of why it improves generalization ability. For example, is it related to reducing intra-batch distribution confusion and decreasing the model's dependence on spurious correlation features?

6. The article mentions using the Kruskal-Wallis test and the Wilcoxon test for comparison, but does not explain the correction method for multiple comparisons (e.g., Bonferroni correction). Without correction in multiple comparisons, the Type I error rate may increase. It is recommended to supplement the explanation of whether multiple comparison correction was performed.

7. Although the authors provide small-scale sample code, overall reproducibility is limited because the core model and proprietary data cannot be publicly disclosed. It is recommended to disclose the model weights or provide an API interface, to the extent possible, to facilitate other researchers' validation and expansion of the research.

8. The figures in the article are of very poor quality. All figures are poorly readable. Many images in the paper are simply screenshots, and some even contain spelling errors.

9. The article title and abstract mention "clinically fair," but the text does not systematically evaluate the model's performance differences across different genders, races, or socioeconomic groups. It is recommended to supplement this with quantitative analysis of fairness, such as group AUC or MAE difference tests.

Reviewer #2: Some grammatical errors (e.g., "Deep learning models, often struggle with generalisation").

Some claims would be strengthened by citations (e.g., "This challenge is further compounded by significant heterogeneity across ECG cohorts, stemming from differences in age distribution, clinical profiles, recording devices, and institutional practices.").

"a retinal image–based foundational model trained on British patient data demonstrated poor transferability to Chinese cohorts, performing comparably to model pretrained on natural images."

- I might clarify this with, "Explorations performed on other medical data modalities...", otherwise it's jarring.

"Initially, CAPE models are pretrained to learn generic ECG features, which are then generated for each labeled cohort. This approach significantly reduces training time for downstream tasks by leveraging these precomputed features."

- This is essentially a linear probing strategy, which is often worse performance than full fine-tuning, and therefore more impressive if done well. You might consider addressing this? Especially if/since you're not receiving SOTA results, which I don't believe is your objective here.

- I do see that you address this in your limitations, but again, you might benefit from better framing here.

While I understand the constraints you're working with due to different label composition, age and sex tasks are hardly clinically salient. At the very least, I feel this should have been addressed more clearly/honestly.

- Coming back to this comment, I do now see that you've also done reduced LVEF and five-year mortality. If appropriate, I may have highlighted this earlier on, and also made this discussion bigger, because it lends clinical significance to your work!

AUROC is not always a robust metric when dealing with different label prevalence. I can't say whether you should include this metric in the paper, but I would make sure that Area Under the Precision-Recall-Gain curve (AUPRG) agrees with your AUROC-based conclusions (I've found this useful in my own work, at least).

"This design discourages the model from learning spurious cohort-distinguishing features and instead promotes the identification of clinically meaningful variability within each cohort."

- I might have put a line like this into your abstract. It's an intriguing design! Though I was curious if any prior works used anything like it in other contexts.

"CAPE models pretrained on secondary care cohorts outperform those pretrained on larger but healthier populations. A plausible explanation for this is that healthier populations tend to exhibit reduced variability, whereas secondary care cohorts encompass a diverse range of cardiac signal patterns."

- You could likely strengthen this claim with some basic statistical analysis of the label distributions

"This suggests that our random sampling from patient ECGs at the training time can successfully exploit the diversity in ECGs belonging to one patient."

- I would want to see more evidence behind this claim.

I would have talked about your reduced LVEF and five-year mortality tasks more in the discussion.

"A comparable work explores domain generalisation for ECG datasets from different sources"

- References are sometimes sprinkled in disruptively? The work could generally benefit from a little more informational organization.

"We anticipate that training the prediction head on the full cohort, followed by fine-tuning the model, could further improve performance."

- This is true all of ML. Consider removing.

"The ECGs for the same patient are treated as positive views while all other ECGs in the batch are negative views."

- Is this inspired by another paper? You don't seem to motivate this choice of contrastive method anywhere, and this study begs the question: Would your results hold across other forms of contrastive learning? E.g., same-sample pairs (temporal crops), or other options. Feels like this should be discussed more.

I found the supposed ethnicity clustering within Figure 4b confusing.

Typo in Figure 6.

Reviewer #3: I appreciate the scale of this work. The effort to systematically evaluate and explor how cohort composition impacts contrastive pretraining in ECG-based foundation models. The findings related to distribution shift and OOD performance are particularly interesting as well. A couple of suggestions are made to straighten the presentation.

Relatedly, the review/discussion would benefit from positioning this work within the rapidly evolving foundation model literature across other biomedical modalities. Recent studies have demonstrated that contrastive pretraining strategies and ensemble-based foundation models can meaningfully improve robustness to out-of-distribution samples (Zhao, J., et al. Uncertainty-aware ensemble of foundation models differentiates glioblastoma from its mimics. Nat Commun 16, 8341 (2025). https://doi.org/10.1038/s41467-025-64249-6), and contrastive learning has been applied to enhance fairness in AI systems (Lin, S. Y., et al. (2025). Contrastive learning enhances fairness in pathology artificial intelligence systems. Cell reports. Medicine, 6(12), 102527. https://doi.org/10.1016/j.xcrm.2025.102527). These works suggest that cohort-aware pretraining and contrastive strategies may have broader implications for multimodal clinical AI beyond signal-based applications. Similar conclusions are drawn, so they should be added to the introduction or discussion.

The current downstream evaluation relies predominantly on age and sex prediction tasks, which, while useful as technical benchmarks, are not strongly representative of clinically meaningful deployment scenarios. If the goal is to support claims regarding generalizability of medical foundation models, some additional downstream analyses would strengthen the translational relevance of the work, for example, incorporating broader clinical prediction tasks or fairness-sensitive outcomes beyond demographic inference.

Incorporating discussion of these recent developments would help contextualize the present findings and highlight how future multimodal or ensemble-based integration strategies could further improve robustness and fairness in foundation models for clinical deployment.

For these reasons, I would suggest a revision to include additional downstream analysis (clinically relevant tasks in ECG, e.g., pattern classifications) and to better situate the work within the broader landscape of recent foundation model research in biomedicine.

Reviewer #4: This manuscript presents a systematic investigation of how pretraining cohort composition influences the performance and out-of-distribution (OOD) generalisation of the CAPE contrastive learning framework for ECG foundation models. The study is conducted at impressive scale, utilizing over 5.2 million ECGs from five countries across four continents. The authors additionally propose an In-Distribution Batch (IDB) strategy to mitigate cohort-specific artifact encoding. The topic is timely and clinically relevant. However, several methodological concerns and gaps in experimental validation must be addressed before the manuscript is suitable for publication.

My major comments are:

1) The downstream evaluation is restricted to age regression and sex classification. They might be insufficient to demonstrate the clinical utility of the proposed IDB strategy. The authors can consider include at least one clinically meaningful prediction task.

2) Downstream evaluations rely on frozen features extracted from the pretrained backbone, with only the MLP prediction head trained for each task. The authors acknowledge this limitation in the limitations section, but its implications are substantial. Fine-tuning the backbone could potentially diminishing or reversing the observed OOD benefits of the IDB strategy.

3) A key mechanistic finding of the paper is that t-SNE clusters in CAPE-X representations correspond more strongly to ECG acquisition device differences than to patient ethnicity. This is an important and novel observation. However, the authors do not provide experiment that disentangles the contribution of device-level artifacts from the benefit of IDB. Specifically, it remains unclear whether standard signal harmonization or device-aware preprocessing could achieve similar or superior OOD improvements without requiring a modified training objective.

Reviewer #5: The paper's central claim that In Distribution Batch (IDB) training improves Out Of Distribution (OOD) generalization is interesting. However, it must be noted that this paper focuses on contrastive learning which is only one of many approaches for creating foundation models. I think there should be more commentary in the paper on other approaches for creating foundation models (liked masked autoencoders and generative pretraining) and whether these other approaches have similar challengs with respect to OOD generalization. Also, it would be useful to have a discussion on whether the IDB approach translates to these diffferent approaches for creating foundation models.

The paper choses to use only age and sex as downstream finetuning benchmarks, but it is not clear how well the results translate to downstream tasks like arrhythmia detection. More commentary around this would be useful.

The decision to use frozen features (and no fine-tuning of the feature-extracting backbone fine-tuning) limits the conclusions. It is unclear whether the observed OOD degradation is a property of the pretraining strategy itself or simply an artifact of not adapting the feature-extracting backbone to downstream tasks. The authors acknowledge this as a limitation, but it deserves more emphasis, since fine-tuning might largely resolve the OOD gap.

**********

what does this mean?). If published, this will include your full peer review and any attached files.

**Do you want your identity to be public for this peer review?**  If you choose “no”, your identity will remain anonymous but your review may still be made public.

For information about this choice, including consent withdrawal, please see our Privacy Policy

Reviewer #1: No

Reviewer #2: No

Reviewer #3: No

Reviewer #4: No

Reviewer #5: No

**********

**Figure resubmission:**

**Reproducibility:**

To enhance the reproducibility of your results, we recommend that authors of applicable studies deposit laboratory protocols in protocols.io, where a protocol can be assigned its own identifier (DOI) such that it can be cited independently in the future. Additionally, PLOS ONE offers an option to publish peer-reviewed clinical study protocols. Read more information on sharing protocols at https://plos.org/protocols?utm_medium=editorial-email&utm_source=authorletters&utm_campaign=protocols

# Author comment: Author response to Decision Letter 1

**DOI:** 10.1371/journal.pdig.0001623.r002

4 Jun 2026

Attachment Submitted filename: PLOS-DH Response Khattak.docx

# Peer review: Decision Letter 1

**DOI:** 10.1371/journal.pdig.0001623.r003

**Authors:** Jie-Zhi Cheng, Zhenwei Shi

14 Jul 2026

Data distribution impacts the performance and generalisability of contrastive learning-based foundation models of electrocardiograms

PDIG-D-26-00170R1

Dear Dr Khattak,

We are pleased to inform you that your manuscript 'Data distribution impacts the performance and generalisability of contrastive learning-based foundation models of electrocardiograms' has been provisionally accepted for publication in PLOS Digital Health.

Before your manuscript can be formally accepted you will need to complete some formatting changes, which you will receive in a follow-up email from a member of our team.

Please note that your manuscript will not be scheduled for publication until you have made the required changes, so a swift response is appreciated.

IMPORTANT: The editorial review process is now complete. PLOS will only permit corrections to spelling, formatting or significant scientific errors from this point onwards. Requests for major changes, or any which affect the scientific understanding of your work, will cause delays to the publication date of your manuscript.

If your institution or institutions have a press office, please notify them about your upcoming paper to help maximize its impact. If they'll be preparing press materials, please inform our press team as soon as possible -- no later than 48 hours after receiving the formal acceptance. Your manuscript will remain under strict press embargo until 2 pm Eastern Time on the date of publication. For more information, please contact digitalhealth@plos.org.

Thank you again for supporting Open Access publishing; we are looking forward to publishing your work in PLOS Digital Health.

Best regards,

Jie-Zhi Cheng

Academic Editor

PLOS Digital Health

***********************************************************

**Additional Editor Comments (if provided):**

Four reviewers reached to the same positive opinion. Most concerns have been well addressed by authors. Good job. Therefore, the acceptance of this is recommended.

**Reviewer Comments (if any, and for reference):**

Reviewer's Responses to Questions

**Comments to the Author**

Reviewer #2: All comments have been addressed

Reviewer #3: All comments have been addressed

Reviewer #4: All comments have been addressed

Reviewer #5: All comments have been addressed

**********

publication criteria?

Reviewer #2: Yes

Reviewer #3: Yes

Reviewer #4: Yes

Reviewer #5: Yes

**********

3. Has the statistical analysis been performed appropriately and rigorously?

Reviewer #2: Yes

Reviewer #3: Yes

Reviewer #4: Yes

Reviewer #5: Yes

**********

4. Have the authors made all data underlying the findings in their manuscript fully available (please refer to the Data Availability Statement at the start of the manuscript PDF file)?

The PLOS Data policy

Reviewer #2: Yes

Reviewer #3: Yes

Reviewer #4: Yes

Reviewer #5: (No Response)

**********

5. Is the manuscript presented in an intelligible fashion and written in standard English?

Reviewer #2: Yes

Reviewer #3: Yes

Reviewer #4: Yes

Reviewer #5: Yes

**********

**Reviewer #2:**  Splendid job on this edit! No real notes!

`In our study, methodological comparisons are always performed on the same data, ensuring a one-to-one pairing. For external validation, we further control this by evaluating all models on identical randomly sampled test sets using fixed seeds.` - This is great to hear in terms of cross-method comparison! Another interesting point is actually cross-label comparison (on the same model/cohort) to get a sense of which labels are more difficult for the model to predict. But this is more an interesting observation than a critique!

**Reviewer #3** : The revision is very complete. I'd love to suggest an acceptance upfront.

The authors can reorganize the presentation a bit and move some parts into the supplementary to highlight the key findings.

**Reviewer #4:**  Thanks for the authors' response. I have read the responses and have no further comments.

**Reviewer #5** : (No Response)

**********

what does this mean?). If published, this will include your full peer review and any attached files.

**Do you want your identity to be public for this peer review?**  If you choose “no”, your identity will remain anonymous but your review may still be made public.

For information about this choice, including consent withdrawal, please see our Privacy Policy

Reviewer #2: **Yes:**  Kaden McKeen

Reviewer #3: **Yes:**

Reviewer #4: No

Reviewer #5: No

**********
